# A systematic review of the determinants of job satisfaction in healthcare workers in health facilities in Gulf Cooperation Council countries

**DOI:** 10.1080/16549716.2025.2479910

**Published:** 2025-04-04

**Authors:** Mohannad Alkhateeb, Khaled Althabaiti, Sayem Ahmed, Solveig Lövestad, Jahangir Khan

**Affiliations:** a Department of Health Service and Hospital Administration, Faculty of Economics and Administration, King Abdul Aziz University, Jeddah, Saudi Arabia; b School of Public Health and Community Medicine, Institute of Medicine, University of Gothenburg, Gothenburg, Sweden; c Basic Nursing Sciences Department, College of Nursing, Taif University, Taif, Saudi Arabia; d Health Economics Research Group (HERG), Department of Health Sciences, Brunel University London, Uxbridge, London, UK; e The Västra Götaland Region Competence Centre on Intimate Partner Violence, Gothenburg, Sweden; f Department of Learning, Informatics, Management and Ethics, Karolinska Institute, Stockholm, Sweden; g Department of International Public Health, Liverpool School of Tropical Medicine, Liverpool, UK

**Keywords:** Healthcare workers, job satisfaction, Gulf Cooperation Council countries (GCC), systematic review, determinants

## Abstract

Job satisfaction among healthcare workers is essential for maintaining high-quality care. Previous research has shown different levels of job satisfaction, but there is no comprehensive list of determinants of job satisfaction among healthcare workers. This study aims to provide a comprehensive list of determinants of job satisfaction in Gulf Cooperation Council (GCC) countries (Saudi Arabia, UAE, Bahrain, Kuwait, Oman, and Qatar). A systematic review was conducted following PRISMA guidelines across five databases: PubMed, CINAHL, Web of Science, Cochrane, and Scopus. Two independent reviewers performed data extraction and review using the Critical Appraisal Skills Programme (CASP) quality assessment checklist. The review was undertaken between 1 January 2012 and 4 November 2022. Five hundred titles and abstracts were screened, yielding 73 eligible studies for inclusion in this review. Of the included studies, 60 were carried out in Saudi Arabia (82.2%), six in Oman (8.2%), three in Qatar (4.1%), two in the United Arab Emirates (2.7%), one in Kuwait (1.4%), and one in the Kingdom of Saudi Arabia and the United Arab Emirates (1.4%). The analysis identified 14 key determinants of job satisfaction among healthcare workers in GCC: pay, promotion, co-workers, supervision, fringe benefits, contingent rewards, operating conditions, nature of work, communication, workload, leadership style, relation with patients, demographic variables, and others, such as hospital type. Thus, our study expands on Spector’s nine determinants model of job satisfaction, hence providing a wider and more detail insight into job satisfaction in workplace.

## Paper Context

- **Main findings:** Job satisfaction among health workers is influenced by multiple factors, including pay, promotion, co-workers, supervision, fringe benefits, contingent rewards, operating conditions, nature of work, communication, workload, leadership style, relation with patients, demographic variables, and others, such as hospital type.
- **Added knowledge:** The study provides a comprehensive list of determinants influencing job satisfaction among healthcare workers. In addition to Spector’s nine established domains, it identified four additional determinants: workload, relationship with patients, leadership styles, and demographic variables.
- **Global health impact for policy and action:** Understanding the determinants of job satisfaction can help policymakers develop strategies to support and retain healthcare workers, ensuring continuous and high-quality patient care.

## Background

Job satisfaction among healthcare workers plays a crucial role in shaping productivity, quality, healthcare costs, and overall organizational effectiveness. High job satisfaction is positively linked to increased performance and negatively to turnover and absenteeism. Understanding healthcare workers’ job satisfaction can enhance patient care quality and contribute to the success of healthcare organizations [1].

Job satisfaction has been defined as ‘the pleasurable emotional state resulting from the appraisal of one’s job as achieving or facilitating the achievement of one’s job values’ (pg. 1342) [2] and ‘the extent to which people like (satisfaction) or dislike (dissatisfaction) their jobs’ (pg. 2) [3]. Many theories describe the conceptual framework of job satisfaction. In general, these theories cover the affective feeling of employees towards their job, which could be directed towards the job in general or their attitudes towards specific aspects of it, for example, working conditions, relationships with their colleagues, payment, and to what extent the outcome of the work meets or exceeds their expectations [4].

When employees feel involved in what they do and the running of an organization, they build confidence in their input. In empowering workers, management gives them the freedom to work and act independently. It supports their abilities by providing adequate resources, a favorable climate, and technical and behavioral training. Wu (2019) shared this view in his observation that motivating employees with excellent and favorable working environments increases their efficiency; the employees ought to be satisfied with what they do [5]. This satisfaction can be deciphered from the elements of management and performance, like remuneration, non-monetary benefits, human relationships at work, and working conditions. Failure to strike a proper balance in managing employee satisfaction and output expectations may harm an organization [6]. Job satisfaction has been considered as one of the main factors collectively shaping any organization’s quality and productivity. For this reason, healthcare workers are expected to have an optimal level of job satisfaction to achieve the utmost goals of health services. Several studies have investigated the determinants of job satisfaction among healthcare workers [7–9]. However, not all studies have covered all determinants of job satisfaction or all healthcare workers. For example, studies focused on the level of job satisfaction among doctors [9], nurses [7], and pharmacists [8].

The healthcare system typically includes rules and laws governing the responsibilities and performance of each professional, which are shaped at the macro level (higher authorities). In addition, micro-level factors are related to the broad spectrum of relationships among managers, colleagues, and patients, and their caregivers [10]. Previous research provided various determinants that substantially affect healthcare workers’ job satisfaction. Among these determinants, the relevant literature reflected the working environment, opportunity for professional growth and development, staff relationship, financial incentives, supportive supervision, work flexibility, work demands, balance between work life and extra work, and resources [10–13].

However, the priorities and effect size of those determinants differ significantly between countries and settings [14]. For example, studies in Ethiopia reported that the crucial determinants that negatively influence job satisfaction are limited resources, such as lack of advanced technologies and low compensation, including low salary, duty allowance, housing, and transportation allowance [14]. Leadership style came on top of the determinants that affect job satisfaction in Saudi Arabia [15]. Girma et al. (2021) emphasized the significant impact of the personal relationship between health workers on job satisfaction and that the health system could influence this relationship at the macro level [10]. Even the factors associated with the nature of the healthcare profession, such as stress and professional time among dentists, were found to be determinants of low satisfaction [16]. Another research study on the job satisfaction of nurses found a negative link between age and level of job satisfaction. A decrease of 3.7% in satisfaction scores was estimated for every one-year age increase [17].

Gulf Cooperation Council (GCC) member states, comprising Bahrain, Kuwait, Oman, Qatar, Saudi Arabia, and the United Arab Emirates (UAE) [18], share similarities in culture, language, geography, religion, society, economics, and legal systems [19]. Healthcare systems in the GCC face various challenges [18]. For instance, GCC countries depend significantly on expatriate health professionals to meet their healthcare needs [20]. In addition, healthcare financing depends mainly on oil revenue, which is susceptible to price changes [21]. They moved toward a sustainable system through privatization and private-sector participation to address these challenges and decrease the public funding burden [22]. This change eventually linked to changing the health workforce’s job contracting policy, which may affect job satisfaction [23].

Herzberg’s two-factor theory, the hygiene-motivation theory, is the most appropriate for addressing job satisfaction factors. It classifies the factors into intrinsic (motivation) and extrinsic (hygiene) to job satisfaction. Motivational factors related to job satisfaction include advancement, the work itself, the possibility of growth, responsibility, recognition, and achievement. Hygiene factors related to job dissatisfaction include interpersonal relations, salary, company policies and administration, relationships with supervisors, and working conditions [24,25].

Understanding the determinants of job satisfaction can provide valuable insights into how to create a positive work environment that will reflect the physical and mental health of healthcare workers, reduce turnover rate, and improve work engagement, job performance, and organizational commitment with the ultimate optimization of the quality of health services [26]. Providing a comprehensive list of the determinants affecting job satisfaction in healthcare workers is challenging due to the complexity of the healthcare system [10]. Therefore, the current study aims to provide a comprehensive list of determinants of job satisfaction among healthcare workers in GCC countries.

## Method

This paper followed the Preferred Reporting Items for Systematic Reviews and Meta-Analysis (PRISMA) standards. Articles were gathered between 10 October 2022 and 4 November 2022. This systematic review has been registered under the PROSPERO ID CRD 42,022,369,754.

### Database and search terms

Data were extracted from five scientific databases, PUBMED, CINAHL, SCOPUS, Web of Science, and Cochrane, to identify relevant English-language articles indexed between 2012 and 2022. We used an appropriate combination of medical subject heading (MeSH) terms and text words (ti, ab, kw) to search the databases to ensure a broad range of relevant studies. Identifying the articles to be included in the study involved specific keywords: healthcare workers, job satisfaction, and GCC countries (Table 1). These keywords were chosen because they align with the study’s aim. Synonyms and differences in spelling were accounted for as well. The exact search phrase used in PubMed is the following:(((‘Health Personnel’ [MeSH Terms] AND ‘Job Satisfaction’ [MeSH Terms]) OR ‘Health Workers’[Title/Abstract] OR ‘Job Satisfaction’[Title/Abstract]) AND (‘Saudi Arabia’[MeSH Terms] OR ‘Oman’ [MeSH Terms] OR ‘United Arab Emirates’ [MeSH Terms] OR ‘Qatar’ [MeSH Terms] OR ‘Kuwait’ [MeSH Terms] OR ‘Bahrain’ [MeSH Terms] OR ‘KSA’[tiab] OR ‘UAE’[tiab] OR ‘Emirate’[tiab])) AND (2012:2022[pdat]). (Supplemental 1).

**Table 1.** Search terms used in electronic database searches.

SEARCH TERMS	
Healthcare Workers “Health Personnel” [Mesh] 213,791 results AND	Personnel, Health Health Care Providers Health Care Provider Provider, Health Care Healthcare Providers Healthcare Provider Provider, Healthcare Healthcare Workers Healthcare Worker Health Care Professionals Health Care Professional Professional, Health Care
Job satisfaction “Job Satisfaction” [Mesh] 9,445 results AND	Job Satisfactions Satisfaction, Job Satisfactions, Job Work Satisfaction Satisfactions, Work Satisfaction, Work Work Satisfactions
GCC Countries (“Saudi Arabia” [MeSH Terms] OR “Oman” [MeSH Terms] OR “United Arab Emirates” [MeSH Terms] OR “Qatar” [MeSH Terms] OR “Kuwait” [MeSH Terms] OR “Bahrain” [MeSH Terms] OR “KSA” [tiab] OR “UAE” [tiab] OR “Emirate” [tiab])) AND (2012:2022 [pdat]) 17,193 results AND	Saudi Arabia Kuwait United Arab Emirates Qatar Bahrain Oman.
After narrowing the search key 127 results	(((“Health Personnel” [MeSH Terms] AND “Job Satisfaction” [MeSH Terms]) OR “Health Workers” [Title/Abstract] OR “Job Satisfaction” [Title/Abstract]) AND (“Saudi Arabia” [MeSH Terms] OR “Oman” [MeSH Terms] OR “United Arab Emirates” [MeSH Terms] OR “Qatar” [MeSH Terms] OR “Kuwait” [MeSH Terms] OR “Bahrain” [MeSH Terms] OR “KSA” [tiab] OR “UAE” [tiab] OR “Emirate” [tiab])) AND (2012:2022 [pdat])

### Eligibility criteria

The inclusion and exclusion criteria determined the guidelines for choosing the articles for the study. The study used the PICO question to establish the inclusion and exclusion criteria (Table 2).

**Table 2.** Inclusion and exclusion criteria.

PICO	Inclusion criteria	Exclusion criteria
Population	Healthcare workers in GCC countries	Healthcare workers from other countries
Intervention	Measuring the level of job satisfaction	———————-
Comparison	No comparison, determinants of job satisfaction.	———————-
Outcome	Job satisfaction among healthcare workers	———————
Language	English	Non-English
Time horizon	From 2012 until 2022	Before 2012

### Paper selection process

The articles used in the research were chosen systematically. The first step included searching for relevant articles using the keywords. The next step included focusing the search using the inclusion and exclusion criteria. Since abstracts summarize the articles’ content, reading them would be necessary to find the papers that may be used in the research [27]. For this purpose, Rayyan’s online application was used, and the articles retrieved based on the search strategy were uploaded. Rayyan is an online website that assists researchers in screening and reviewing articles for systematic reviews and is useful in collaborative work [28]. Two reviewers (MA and KA) independently reviewed the title and abstract uploaded to decide which articles should be included in the study. The two reviewers checked for consensus and discussed it for their approval in case of disagreement. Conflicting views were discussed with other authors (JK, SL, SA). The full texts of the articles that were finally included were then prepared for data extraction.

### Data extraction/synthesis and data analysis

An Excel sheet was produced for data extraction. The two reviewers independently extracted the data. Each author/reviewer extracted the data on general information and detailed study characteristics. General information included the researcher performing data extraction, Data extraction date, Study ID number, Article title, Citation, publication type, Publication year, Country of origin, and Source of funding. Detailed study characteristics contained information on the aims/objectives and the study’s country, type of health center (Primary health center, general hospital, tertiary healthcare, etc.), inclusion/exclusion criteria, primary findings, and additional findings (Supplemental 2).

### Assessment of quality of studies

Ensuring a review provides the best evidence available requires evaluating a study’s quality. Critical Appraisal Skills Program (CASP) checklists were utilized to evaluate study quality [29]. Multiple checklists were specified for each study design. As all included studies were cross-sectional, qualitative, and systematic reviews, the checklists were used to appraise them critically. In general, the checklists helped assess the articles’ fundamental construct and content, including appropriateness of the reporting, external validity, study power, and bias (Supplemental 3).

## Results

**Figure 1.**
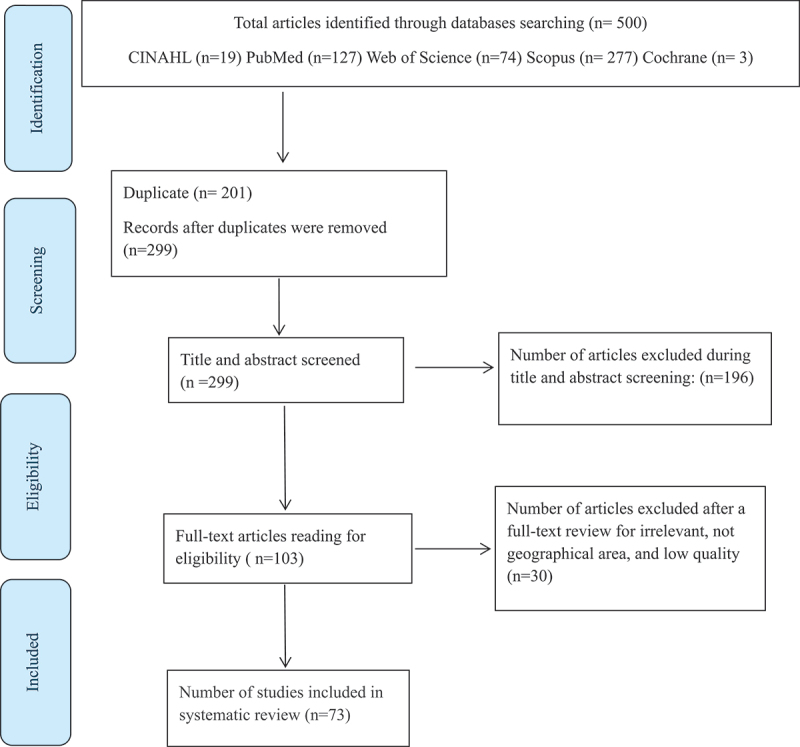
Flowchart showing the search results.

Five hundred studies were identified from five electronic databases published between 2012 and 2022 (Figure 1). After removing duplicates, 299 titles and abstracts were screened for eligibility against inclusion and exclusion criteria, which excluded 196 studies. The full text of the remaining 103 studies was retrieved and screened for eligibility, and 30 were excluded for being irrelevant, not the appropriate geographical area, and low quality. In total, 73 studies were included in the systematic review and met the inclusion criteria.

Of the 73 papers identified for the review, most studies were conducted in the Kingdom of

Saudi Arabia (*n* = 60), followed by Oman (*n* = 6), Qatar (*n* = 3), UAE (*n* = 2), Kuwait (*n* = 1), and KSA and UAE (*n* = 1). Sixty-eight studies were cross-sectional, three were qualitative, and two were systematic reviews.

The reviewed studies were conducted in different healthcare facilities: 67 were performed in hospitals, five in Primary Health Care (PHC), and one in a medical call center (on-call remote physicians). The hospital studies included 23 general hospitals, 11 tertiary hospitals, 9 teaching hospitals, and 24 multi-center hospitals. Forty-six studies were conducted in the governmental sector, three in the private sector, and 24 in mixed sectors (governmental and private).

The population of the studies covered a broad spectrum of healthcare workers; most studies (*n* = 30) focused on nurses, followed by studies of all healthcare workers (*n* = 12), allied healthcare professionals (*n* = 12), physicians (*n* = 10), pharmacists (*n* = 5), and dentists (*n* = 4).

A diversity of questionnaires measured job satisfaction in this systematic review. The most commonly used instruments were the Job Satisfaction Survey (15 studies), the Minnesota Satisfaction Questionnaire (5 studies), the Warr-Cook-Wall scale (4 studies), the McCloskey/Mueller Satisfaction Scale (4 studies), the Job Descriptive Index (4 studies), the Measure Job Satisfaction (3 studies), the Job Satisfaction Scale (2 studies), single item (6 studies), the Nursing Workplace Satisfaction Questionnaire (1 study), Minnesota Satisfaction Questionnaire & Warr-Cook-Wall (1 study). In addition, 28 studies used genuine tools developed by the authors.

The reviewed studies adopted many definitions of job satisfaction. For example, Hoppock (1935) defined it as ‘a combination of psychological, physiological and environmental circumstances that causes a person to say: I am satisfied with my job’ [30]. Spector (1985) stated that job satisfaction is ‘how people feel about their jobs and different aspects of their jobs. It is the extent to which people like (are satisfied with) or dislike (are dissatisfied with) their jobs’ [31]. In addition, Cumbey and Alexander (1998) defined job satisfaction as ‘an affective feeling that depends on the interaction of employees, their personal characteristics, values, and expectations with the work environment and the organization’ [32]. Lately, Nelson and Quick (2013) defined it as ‘a pleasurable or positive emotional state resulting from the appraisal of one’s job or job experiences’ [33].

### Determinants of job satisfaction (Figure 2)

#### Pay (40/73 studies)

Of the 73 reviewed studies, 40 stated that pay was one important determinant affecting health workers’ job satisfaction. Pay was mentioned in different terms with the same meaning. For instance, the majority of studies used the word ‘pay’ (17 studies) [8,9,12,15,34–46], ‘salary’ (12 studies) [47–58], and ‘income’ (5 studies) [59–63]. Pay was reported as a determinant in most of the studies on physicians (7/10) [9,12,42,54,55,61,64], allied healthcare professionals (9/12) [15,40,45,47,56,57,63,65,66], nurses (12/30) [34–39,46,48,51,52,67,68], pharmacists (3/5) [8,49,58], dentists (3/4) [59,60,69], and all healthcare workers (6/12) [41,43,44,50,53,62].

**Figure 2.**
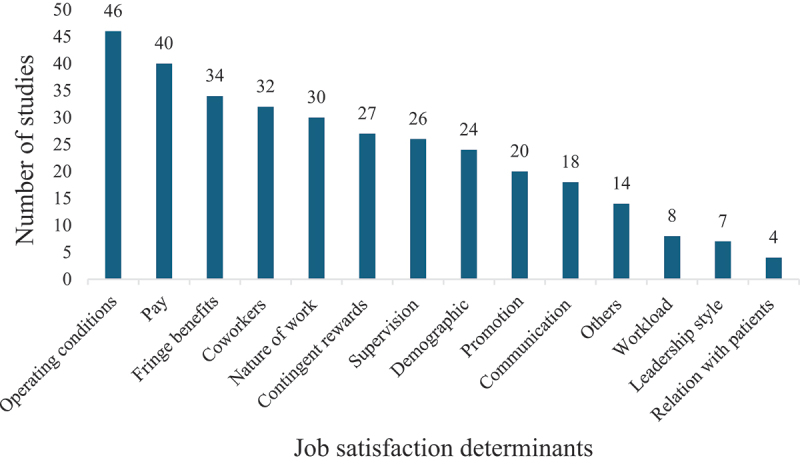
Frequency of job satisfaction determinants reported in reviewed studies.

Pay: pay and remuneration; promotion: promotion opportunities; co-workers: people you work with; supervision: immediate supervisor, fringe benefits: monetary and non-monetary fringe benefits; contingent rewards: appreciation, recognition, and rewards for good work; operating conditions: operating policies and procedures; nature of work: job tasks themselves; communication: communication within the organization; demographic: age, gender, etc.; leadership style: transformational and transactional leadership styles; relation with patients: ‘respect received from the patients,’ attitude and adherence; workload: too much work; others: e.g. effort-reward imbalance, low back pain.

#### Promotion (20/73 studies)

Of the 73 screened studies, 20 studies [8,9,12,15,34–43,45,46,49,56–58] included promotion as a job satisfaction determinant that increases job satisfaction among healthcare workers. No other word was found referring to promotion in the studies reviewed. Promotion was included in studies related to nurses (7/30 studies) [34–39,46], followed by allied health care professionals (5/12 studies) [15,40,45,56,57], physicians (3/10 studies) [9,12,42], pharmacists (3/5 studies) [8,49,58] and all healthcare workers (2/12) [41,43].

#### Coworkers (32/73 studies)

Co-workers refer to ‘people with whom one works,’ defined in different terms with the same meaning. It was identified as a job satisfaction determinant in 32 studies. For instance, most studies used the word co-workers (22 studies) [9,12,15,34,35,37,39–43,45–47,49,56,57,65,66,70–72], then colleagues (7 studies) [8,38,48,59,60,73,74] and teamwork (3 studies) [7,51,75]. Co-workers was used in conjunction with studying job satisfaction among nurses (13/30 studies) [7,34,35,37–39,46,48,51,70,71,73,75], allied healthcare professionals (8/12 studies) [15,40,45,47,56,57,65,66], physicians (3/10 studies) [9,12,42], dentists (3/4 studies) [59,60,74], all healthcare workers (3/12 studies) [41,43,72], and pharmacists (2/5 studies) [8,49].

#### Supervision (26/73 studies)

Twenty-six studies included supervision as a determinant of job satisfaction, which may affect healthcare workers positively or negatively depending on their relationship. Most studies used the term ‘supervision’ (21 studies) [9,12,15,34,35,37–43,45,46,50,65,66,71,72,76,77]; other studies used the ‘manager’ or ‘leadership’ to indicate supervision [8,49,56,57,73]. Eight studies examined supervision in articles related to nurses [34,35,37–39,46,71,73], allied healthcare professionals (*n* = 8) [15,40,45,56,57,65,66,77], physicians (*n* = 4) [9,12,42,76], all healthcare workers (*n* = 4) [41,43,50,72], and pharmacists (*n* = 2) [8,49].

#### Fringe benefits (34/73 studies)

Of the 73 reviewed studies, 34 mentioned fringe benefits in different terms or content. However, the majority of studies used the term ‘fringe benefits’ (10 studies) [12,15,34,35,37,38,40,41,43,46]. Employers or organizations provide fringe benefits to employees or workers in the same organization, including many things, such as insurance, bonuses, job security, or retirement. Many studies articulated fringe benefits as a determinant of job satisfaction in healthcare. For example, it was articulated in studies related to nurses (*n* = 12) [34–38,46,48,51,70,78–80], then allied healthcare professionals (*n* = 9) [15,40,47,56,57,63,65,66,77], all healthcare workers (*n* = 6) [41,43,50,53,62,72], physicians (*n* = 3) [12,54,64], pharmacists (*n* = 3) [8,49,58], and dentists (*n* = 1) [74].

#### Contingent rewards (27/73 studies)

Twenty-seven studies examined contingent rewards as a determinant of job satisfaction using many meanings, such as freedom to choose their work method, accomplishment of work, independence, motivational talks, creativity, autonomy, recognition, and opportunity to use ability in the workplace. Of the 27 studies, eight studies were related to nurses [34–38,46,52,70], then allied healthcare professionals (*n* = 8) [15,40,47,56,57,63,65,66], all healthcare workers (*n* = 4) [41,43,53,62], physicians (*n* = 3) [12,54,76], pharmacists (*n* = 3) [8,49,58], and dentists (*n* = 1) [59].

#### Operating conditions (46/73 studies)

The most commonly reported determinant of job satisfaction was the ‘operating conditions,’ which appeared in 46 studies. This term has many meanings, such as work environment, organizational policies, health and safety, working hours, resources, facilities, and quality of services. However, most studies defined ‘operating conditions’ using the same words. Three studies mentioned stress under ‘operating conditions’ as negatively linked with job satisfaction [56,69,81].

Eighteen studies covered operating conditions as a job satisfaction determinant in nurses [7,34,35,37–39,46,48,51,52,68,73,79,82–86], followed by all healthcare workers (*n* = 8) [41,43,44,50,62,72,87,88], allied healthcare professionals (*n* = 8) [15,40,56,57,63,65,66,77], physicians (*n* = 4) [12,54,64,89], dentists (*n* = 4) [59,60,69,74], and pharmacists (*n* = 4) [8,49,58,81].

#### Nature of work (30/73 studies)

Nature of work refers to ‘job tasks and type of work performed.’ It emerged as a key determinant of job satisfaction in 30 studies. Five studies specifically focused on the variety of jobs [8,59,65,66,89], and one considered health workers serving during the Hajj season [89], an annual pilgrimage of Muslims from all over the world to Saudi Arabia. One study explored the unique context of teaching activity as a part of the nature of work within a tertiary and research center [64]. The nature of work was a substantial determinant of job satisfaction among all healthcare workers particularly for nurses (9/30) [34,35,37–39,46,52,71,79], physicians (6/10) [9,12,42,64,76,89], allied healthcare professionals (6/12) [15,40,45,47,65,66], dentists (2/4) [59,60], pharmacists (2/5) [8,90], and all healthcare workers (5/12) [41,43,72,87,91].

One study addressed role ambiguity, conflict, and skill underutilization [91], and another mentioned the perception of favoritism as a nature of work characteristic negatively associated with job satisfaction [79].

#### Communication (18/73 studies)

Eighteen studies included ‘communication’ as one determinant of job satisfaction among healthcare workers, which referred to all sorts of communication between healthcare staff and patients. The word ‘communication’ was mentioned in most studies (*n* = 15) [12,15,34,35,37,38,40,41,43,46,51,68,71,74,80]. However, three used different words, such as HR support [49] and interpersonal relationships [50,92]. Communication was a salient determinant of job satisfaction in Nurses (10/30) [34,35,37,38,46,51,68,71,80,92], allied healthcare professionals (2/12) [15,40], physicians (1/10) [12], pharmacists (1/5) [49], dentists (1/4) [74], and all healthcare workers (3/12) [41,43,50].

#### Demographics (24/73)

Of the 73 studies reviewed, 24 pointed to demographic characteristics as a job satisfaction determinant, which could positively or negatively affect the job satisfaction of healthcare workers such as physicians, nurses, and allied healthcare professionals. The results showed an inconsistent relationship between job satisfaction and age, gender, and nationality. For example, in some studies, the middle-aged [77] were more satisfied with their job, while others showed that older workers were more satisfied [9]. Males were reported in some studies to be more satisfied with their jobs [52], while other studies showed that females were more satisfied [15]. Also, some studies reported that Saudis were more satisfied with their jobs [12], while others showed that non-Saudis were more satisfied with their jobs [52].

Eleven studies reported demographic variables as job satisfaction determinants in articles related to nurses [39,51,52,68,70,73,79,80,82,92,93], followed by allied healthcare professionals (5/12) [15,63,65,66,77], physicians (2/10) [9,64], pharmacists (2/5) [8,81], dentists (1/4) [60], and all healthcare workers (3/12) [62,72,87].

#### Leadership style (7/73)

Only seven studies [15,83,93–97] explicitly examined leadership style as a determinant of job satisfaction, with four finding a positive association between transformational leadership and employee satisfaction [83,93–95]. Two other studies suggested a broader impact of transformational and transactional leadership [15,96]. One study investigated multiple leadership styles with less specific results [97].

#### Relation with patients (4/73)

Four studies [44,54,60,74] identified ‘relation with patients’ as a job satisfaction determinant, focusing on ‘respect received from the patient,’ attitude, and adherence as potentiating for job satisfaction. Two studies [60,74] were related to dentists, one study [54] was related to physicians, and one study [44] was related to all healthcare workers.

#### Workload (8/73)

‘Workload’ was linked with job dissatisfaction in most reviewed studies, including eight [36,44,51,54,56,57,71,91]. Only one term, ‘workload,’ was used. Most studies were related to nurses (3/30) [36,51,71], then allied healthcare professionals (2/12) [56,57], all healthcare workers (2/12) [44,91], and physicians (1/10) [54].

#### Others (14/73)

Other job satisfaction determinants were found sporadically in the reviewed studies, including effort-reward imbalance, utilization of electronic medical records, administrative duties and paperwork, hospital and department type, low back pain, and moral values. Moral values were used in two studies [65,66], followed by administration (*n* = 2) [50,60] and effort-reward imbalance (*n* = 2) [98,99].

Five studies included a variety of job satisfaction determinants related to nurses [39,71,80,82,100], followed by allied healthcare professionals (*n* = 4) [65,66,98,99], physicians (*n* = 2) [54,101], all healthcare workers (*n* = 1) [50], pharmacists (*n* = 1) [81], and dentists (*n* = 1) [60].

## Discussion

This systematic review explored the determinants of health workers’ job satisfaction in the Gulf Cooperating Council countries (GCC) from 2012 to 2022. Seventy-three studies were reviewed, most conducted in Saudi Arabia (*n* = 60) and on nurses. Sixty-eight studies used cross-sectional methods, three were qualitative, and two were systematic reviews.

Eight tools were used to assess health workers’ job satisfaction. Fourteen determinants were identified as independent predictors of job satisfaction: pay, promotion, co-workers, supervision, fringe benefits, contingent rewards, operating conditions, nature of work, communication, demographics, leadership style, relation with patients, workload, and other determinants, such as low back pain and moral value.

‘Pay’ is a crucial factor influencing job satisfaction among healthcare workers, consistent with Herzberg’s theory [102,103]. However, pay appears to have a dual effect when healthcare workers perceive their salary as attractive [50], fair [67], and balanced with their qualifications and profession, meaning that the pay is positively linked with job satisfaction [52]. Conversely, it is negatively linked with job satisfaction when perceived as unbalanced with work effort [47] and inadequate compensation, particularly compared to colleagues in other sectors [59].

Promotion was one of the strongest determinants of job satisfaction, aligning with Herzberg’s two-factor theory under extrinsic motivational factor [103]. Promotion is a sign of career advancement [58], and in most instances, it is conditioned by the achievement of organizational goals [104]. A study in four hospitals in Saudi Arabia revealed that promotion was the second strongest motivator of job satisfaction after salary. However, it showed that pharmacists were uncertain about effective promotion opportunities; this uncertainty was attributed to the unavailability of a clear promotion policy and challenges in budgeting [58]. In Oman, promotion was dissatisfactory, based on an unfair and unclear system, ignoring years of experience and individual work efforts [56,57]. However, another study in Saudi Arabia shows that promotion could be a source of stress for healthcare workers, as promotion demands increased employees’ efforts and responsibilities [46].

Job satisfaction was influenced by the quality of relationships under Herzberg’s two-factor theory, which asserts that a harmonious relationship is a positive motivator for the worker [103,105]. Healthcare workers are more satisfied with perceived good interpersonal relations and friendship with their colleagues [70], working in teams [72], and receiving needed support from peers [42] to accomplish complex tasks. Effective communication and the ability to rely on colleagues are crucial for job satisfaction among nurses. This aspect cannot be ignored because team interaction and care efficacy are closely linked [38]. An Omani study on medical laboratory professionals argued that co-worker relationships were a highly positive motivating factor that improved workers’ job satisfaction [57]. The results are consistent with a study conducted in Canada, which found that good relationships between nurses and physicians significantly enhance job satisfaction, particularly in the perioperative setting, where nurses interact closely with physicians and other team members for a long time [106].

Job satisfaction was linked with supervision based on Herzberg’s two-factor theory under hygiene factors [103]. Healthcare workers appear more satisfied when dealing with active rather than passive/avoidant supervisors [15]. Conversely, they are dissatisfied with less than optimum supervision and low respect [56]. A Saudi Arabian study found that supervision was a significant and positive predictor of job satisfaction among nurses [71]. These results aligned with a study conducted in Pakistan among healthcare workers in teaching hospitals, which showed that they were satisfied with supervisors, especially regarding their skills and capabilities and the critical role they play in mentoring [107], the mutual respect, recognition, and equity of workload distribution [108]. In comparison, frequent punishment and negative feedback were important predictors of dissatisfaction [109,110].

Fringe benefits encompass ‘monetary and non-monetary benefits (e.g. sick pay, health insurance, annual leave, continuing professional development opportunities)’ [40]. Fringe benefits play a critical role in job satisfaction among healthcare workers, and their presence has been shown to impact job satisfaction positively. For instance, the availability of benefits like professional development and training in Oman [57] and Kuwait [72], adequate vacation time, and health coverage for the employee and family in Saudi Arabia [64] were linked to higher job satisfaction. Conversely, the lack of these benefits can lead to dissatisfaction, such as when there is a shortage of educational and training opportunities [47,54,79], a lack of job security for expatriates [51], and professional support [53].

Contingent rewards are described as ‘rewards, appreciation, and recognition given for adequate work (e.g. attending international symposiums, granting flexibility and autonomy in daily tasks)’ [40]. The study revealed that having access to contingent rewards that depend on performance can positively impact job satisfaction among healthcare workers. This is demonstrated by dentists in the private sector in Saudi Arabia, who showed that the freedom of work positively affected job satisfaction as it indirectly increased their income [59]. Also, workers in private settings who received more recognition and appreciation for their efforts and achievements were more satisfied [53]. Nevertheless, the study showed that failure to provide various contingent rewards on performance can result in dissatisfaction. This result was observed in primary health care workers in Saudi Arabia who were dissatisfied with contingent rewards due to a lack of mechanism of work incentives based on performance [12]. Moreover, the lack of autonomy in Oman has been viewed as a source of dissatisfaction [56].

‘Operating conditions’ was the most frequent determinant among healthcare workers. Action regulation theory provides a theoretical framework for understanding the factors influencing operating conditions. Two key components, the process of action and associated regulations, shape operating conditions [111]. The concept of ‘process’ showed itself in the reviews of literature in various aspects, such as ‘service type’ [51] and ‘ability utilization’ [66], while ‘regulations’ were defined in ‘sick leave policy’ [64] and ‘working in a day shift’ [39].

The reviewed literature showed a paradoxical impact of operational conditions on job satisfaction, depending on the availability of resources. For example, while operating conditions were negatively linked with physiotherapists’ job satisfaction in Saudi Arabia [15], similar findings reported in Jordan [112] were partly explained by the ambiguity in the regulation of governing contingent rewards and operating procedures [113]. On the other hand, in Saudi Arabia, findings revealed a positive association between job satisfaction and regular work hours (regulation), suggesting that predictable schedules contribute to a healthy work–life balance [64]. Similar results were found in Turkey, where positive job satisfaction among health professionals in an emergency department was linked to the freedom of choice of the shift type [114]. Moreover, a lack of safety culture was reported as a source of dissatisfaction among medical laboratory technologists (MLTs) in university hospital in Oman [56].

By definition, the nature of work includes ‘job tasks and type of work performed’ [40]. The study showed that the nature of work positively impacts job satisfaction among healthcare workers [43]. For instance, in Saudi Arabia, physicians showed higher satisfaction in academic and research involvement due to prestige and confidence [64]. Physicians working onsite were more satisfied than those working remotely in medical call centers, which was attributed to the opportunity to exchange experiences with senior colleagues [76]; moreover, working in mass gatherings, such as Hajj period in Saudi Arabia, was more satisfactory for surgeons due to the variety of clinical cases [89]. In Kuwait, health professionals were satisfied regarding the understanding of work procedures and implementation [72], and in Qatar, satisfaction was correlated with personal accomplishment among psychiatrists [42]. On the other hand, in Qatar, dissatisfaction was derived from conflict and ambiguity of roles and responsibilities, besides skill underutilization among all healthcare workers in public hospitals [91]. Favoritism, lack of justice, and transparency in working were related to dissatisfaction among nurses in Saudi Arabia [79].

Communication involves transparent and effective communication between healthcare providers, who must be capable of accurately sharing patients’ information, discussing treatment plans, and ensuring clarity of roles and responsibilities for all involved [115]. The reviewed literature showed that communication positively predicted nurses’ job satisfaction during Hajj (the annual gathering of global pilgrims) in Saudi Arabia [71]. Another study carried out in the UAE among dental practitioners found that the high level of satisfaction in the communication between dentists and staff was attributed to the comfort in relationships within the working environment [74]. However, a qualitative study conducted in Saudi Arabia between nurses of different nationalities showed their dissatisfaction with communication due to the language barriers as a fundamental determinant influencing job satisfaction [68]. Abuse from patients and their families was a source of dissatisfaction among expatriate nurses in the ICU that was attributed to discrimination in Saudi Arabia [92].

The review showed that nationality, age, gender, years of experience, and education level influence job satisfaction inconsistently. Several studies observed that several demographic factors of employees influence job satisfaction. For example, studies showed that citizenship influences job satisfaction, where expatriate nurses showed higher job satisfaction than citizen nurses, which is attributed to the higher job expectations among citizen nurses, which might not be fulfilled [52,82].

Moreover, older and more experienced nurses were more satisfied than younger nurses in Oman [82], and a similar relationship was found among the doctors in Saudi Arabia [9]. Older healthcare professionals exhibit higher levels of job satisfaction due to solid commitment to the organizations than younger professionals [9].

Most studies in Saudi Arabia revealed that female healthcare professionals were more satisfied than males due to different perceptions and expectations of job satisfaction in public and private hospitals [15,65,77]. The reason why women generally tend to be happier at work than their male counterparts [77] might be due to lower expectations attributed to cultural reasons [116].

A positive association was found between job satisfaction and years of experience. Studies conducted in Oman [82] and Saudi Arabia [39,73] showed more job satisfaction for more years of experience. This association between increased years of experience and higher job satisfaction can be attributed to the expertise and skills gained over time in the position [39].

Factors like wages influence the relationship between education level and nurse job satisfaction [117]. Although higher education is frequently associated with increased job satisfaction, this is not always the case. When salaries are equal, nurses with lower education are more satisfied than those with higher education [82].

The leadership style of hospital managers plays a significant role in shaping job satisfaction among employees, as they are responsible for creating a work environment where they feel appreciated and motivated; different styles can either enhance or hinder job satisfaction [15,83]. For instance, transformational leadership has been found to be positively linked with job satisfaction among healthcare workers in Saudi Arabia, while transactional leadership has a negative impact [94]. That finding could be attributed to the hypothetical differences between the two styles; the transformational style focuses on inspiring and motivating followers to achieve their full potential through intrinsic factors like shared vision and personal growth, while transactional leadership styles concentrate on achieving goals through extrinsic factors, such as rewards and punishment [118]. One argument is that transactional leadership can be effective for routine tasks, while transformational leadership is ideal for creative and complex organizations, such as healthcare. Interestingly, the leader’s gender has been documented to influence job satisfaction, with more preference for male leaders [93].

Relationships with patients have been found to be essential in job satisfaction among healthcare workers; for example, in Saudi Arabia, orthodontists were satisfied with the respect and attitudes they received from their patients [60]. However, relations with patients could also be a source of stress; for example, patients may occasionally have unrealistic expectations about the treatment outcomes [60] and demand unnecessary procedures, hindering doctors’ ability to provide optimal care and lowering their satisfaction levels [54]. In addition, unreliable patients who are consistently delayed in keeping their appointments could disrupt the flow of work and cause stress for healthcare professionals [60].

There was a link between workload and job dissatisfaction based on Herzberg’s two-factor theory under hygiene factors [57,103]. Heavy workloads can harm medical work, potentially resulting in improper treatment and increased patient risks; both are unsatisfactory for healthcare workers [119]. A study conducted in Oman found that heavy workload influenced job satisfaction negatively among medical laboratory professionals, which was explained by the relative staff shortage [56] and exacerbated by unplanned leave [57]. In Saudi Arabia, qualified resident doctors who worked in the emergency department indicated that workload was one of the themes that negatively influenced job satisfaction, mainly due to overload by non-urgent patients [54]. These findings are consistent with a South African study recommending that workload can be minimized by properly addressing staff shortages and planning duty schedules [120].

Other determinants influence the job satisfaction of the health workforce. For instance, the type of healthcare facilities significantly affects job satisfaction among healthcare professionals. A study in a university hospital in Oman suggested that nurses experience higher satisfaction due to the work environment (collegial nurse–physician relation) [82]. Conversely, pharmacists working in hospitals in Saudi Arabia reported lower satisfaction than community pharmacists because of the heavy workload and performance pressure [81]. Meanwhile, rehabilitation professionals working in non-profit organizations were more satisfied than those in teaching and profit hospitals, attributed to differences in workload and Effort-Rewards Imbalance (ERI) [98].

### Study limitations

Most of the reviewed studies were conducted in Saudi Arabia (*n* = 60), with only one study from Kuwait, two studies from the United Arab Emirates, and three studies from Qatar. This limits the comparison between the GCC countries due to the unbalanced weight in the number of studies. In addition, no study from Bahrain was included.

## Conclusion

This review identified 73 studies about determinants of job satisfaction among healthcare workers in Gulf Cooperation Countries. According to Herzberg’s theory, the determinants can affect the level of job satisfaction of healthcare workers positively or negatively. The determinants are classified into hygiene factors: pay, promotion, contingent rewards, operating conditions, workload, leadership styles, and motivation factors, which are co-workers, nature of work, supervision, fringe benefits, and communication. Factors that were negatively linked to job dissatisfaction were unbalanced pay compared to effort, unfair promotion, poor chances for development and training, lack of autonomy, low incentives, inadequate safety culture, unclear process and procedure, favoritism, abuse from patients and their families, discrimination, and workload with relative shortage of staff. It is recommended that policymakers should review the financial and non-financial incentives, including pay scale, promotion rules, and regulations, fringe benefits, such as lack of funding for training and courses, and contingent rewards like recognition and autonomy, in addition to the nature of work, such as ensuring transparency and preventing conflict, discrimination, and favoritism.

## Supplementary Material

Supplemental Material

## References

[1] Karaferis D, Aletras V, Niakas D. Determining dimensions of job satisfaction in healthcare using factor analysis. BMC Psychol. 2022;10:240. doi: 10.1186/s40359-022-00941-2 36303222 PMC9610349

[2] Locke EA. The nature and causes of job satisfaction. In: Dunnette M, editor. Handbook of industrial and organizational psychology. Chicago, IL: Rand McNally; 1976. p. 1297–15.

[3] Spector PE. Job satisfaction: application, assessment, causes, and consequences. Thousand Oaks, CA: SAGE; 1997.

[4] Lu H, While AE, Barriball KL. Job satisfaction among nurses: a literature review. Int J Nurs Stud. 2005;42:211–227. doi: 10.1016/j.ijnurstu.2004.09.003 15680619

[5] Wu C-H. Implications for employee proactivity research. In: Wu CH, editor. Employee proactivity in organizations: an attachment perspective. Briston (UK): Bristol University Press; 2019. p. 83–103.

[6] Singh Y. Employee engagement as a contemporary issue in HRM. Management techniques for employee engagement in contemporary organizations. Hershey (PA): IGI Global; 2019. p. 20–45.

[7] Labrague LJ, Al Sabei S, Al Rawajfah O, et al. Interprofessional collaboration as a mediator in the relationship between nurse work environment, patient safety outcomes and job satisfaction among nurses. J Nurs Manag. 2022;30:268–278. doi: 10.1111/jonm.13491 34601772

[8] Al-Omar HA, Khurshid F, Sayed SK, et al. Job motivation and satisfaction among female pharmacists working in private pharmacy professional sectors in Saudi Arabia. Risk Manag Healthc Policy. 2022;15:1383–1394. doi: 10.2147/RMHP.S369084 35903180 PMC9314753

[9] Almarashi AM, Al Wadei S, Alajaj AS, et al. Job satisfaction and organizational commitment of doctors: a case study of Saudi Arabia. Jp J Biostat. 2022;19:15–35. doi: 10.17654/0973514322002

[10] Girma B, Nigussie J, Molla A, et al. Health professional’s job satisfaction and its determinants in Ethiopia: a systematic review and meta-analysis. Archiv Public Health. 2021;79:1–11. doi: 10.1186/s13690-021-00664-7 PMC8340440 34353375

[11] Jang Y, Lee AA, Zadrozny M, et al. Determinants of job satisfaction and turnover intent in home health workers: the role of job demands and resources. J Appl Gerontol. 2017;36:56–70. doi: 10.1177/0733464815586059 25956445

[12] Allebdi AA, Ibrahim HM. Level and determinants of job satisfaction among Saudi physicians working in primary health-care facilities in Western Region, KSA. J Family Med Prim Care. 2020;9:4656–4661. doi: 10.4103/jfmpc.jfmpc_428_20 33209779 PMC7652182

[13] Sousa-Uva M, Sousa-Uva A, E Sampayo MM, et al. Telework during the COVID-19 epidemic in Portugal and determinants of job satisfaction: a cross-sectional study. BMC Public Health. 2021;21:1–11. doi: 10.1186/s12889-021-12295-2 34865641 PMC8645416

[14] Abate HK, Mekonnen CK. Job satisfaction and associated factors among health care professionals working in public health facilities in Ethiopia: a systematic review. J Multidiscip Healthc. 2021;Volume 14:821–830. doi: 10.2147/JMDH.S300118 PMC8053506 33880031

[15] Alkassabi OY, Al-Sobayel H, Al-Eisa ES, et al. Job satisfaction among physiotherapists in Saudi Arabia: does the leadership style matter? BMC Health Serv Res. 2018;18:1–9. doi: 10.1186/s12913-018-3184-9 29880040 PMC5992670

[16] Dang M-H, Kim J-G, Yang Y-M, et al. Dentist job satisfaction: a systematic review and meta-analysis. Int Dent J. 2021;71:369–377. doi: 10.1016/j.identj.2020.12.018 PubMed Central PMCID: PMC3877454. 33612262 PMC9275337

[17] Amiresmaili M, Moosazadeh M. Determining job satisfaction of nurses working in hospitals of Iran: a systematic review and meta-analysis. Iran J Nurs Midwifery Res. 2013;18:343–348. PubMed PMID: 24403934. 24403934 PMC3877454

[18] Alsubahi N, Pavlova M, Alzahrani AA, et al., editors. Healthcare quality from the perspective of patients in Gulf cooperation council countries: a systematic literature review. Healthcare. 2024;12:315. MDPI. doi: 10.3390/healthcare12030315 38338200 PMC10855039

[19] Khan S, Qasem A. Are the firms’ capital structure and performance related? Evidence from GCC economies. Cogent Bus & Manag. 2024;11:2344749. doi: 10.1080/23311975.2024.2344749

[20] Sheikh JI, Cheema S, Chaabna K, et al. Capacity building in health care professions within the gulf cooperation council countries: paving the way forward. BMC Med Educ. 2019;19:1–10. doi: 10.1186/s12909-019-1513-2 30871521 PMC6417223

[21] Alkhamis A, Hassan A, Cosgrove P. Financing healthcare in gulf cooperation council countries: a focus on Saudi Arabia. Int J Health Plann Manage. 2014;29:e64–e82. doi: 10.1002/hpm.2213 23996348 PMC4260721

[22] Arab Health The Official Magazine. Privatisation of public healthcare services in the GCC – opportunities, challenges and success factors. Arab Health; 2017 [cited 2025 Jan 27]. Available from: https://www.arabhealthonline.com/magazine/en/latest-issue/3/privatisation-of-public-healthcare-services-in-the-GCC-opportunities-challenges-and-success-factors.html

[23] Ghalibi KM, Ibrahim Omer HM, Al Mamun M. Awareness and readiness of healthcare workers regarding national transformation program in the health sector in Tabuk Region, Saudi Arabia. Saudi J Health Sys Res. 2024;4:45–53. doi: 10.1159/000534781

[24] Herzberg F. Work and the nature of man. Cleveland (OH): World Publishing Company; 1966.

[25] Alshmemri M, Shahwan-Akl L, Maude P. Herzberg’s two-factor theory. Life Sci J. 2017;14:12–16.

[26] Hudays A, Gary F, Voss JG, et al., editors. Factors influencing job satisfaction among mental health nurses: a systematic review. In: Healthcare. Basel (Switzerland): MDPI; 2024. p. 2040. 10.3390/healthcare12202040 PMC11507421 39451455

[27] Lane HC, Zvacek S, Uhomoibhi J. Computer supported education. In: 12th International Conference, CSEDU 2020, Virtual Event, May 2–4, 2020, Revised Selected Papers. Springer Nature; 2021.

[28] Ouzzani M, Hammady H, Fedorowicz Z, et al. Rayyan—A web and mobile app for systematic reviews. Syst Rev. 2016;5:1–10. doi: 10.1186/s13643-016-0384-4 27919275 PMC5139140

[29] Critical Appraisal Skills Programme. Critical appraisal checklists. 2018 [cited 2020 Mar 8]. Available from: https://casp-uk net/casp-tools-checklists/

[30] Hoppock R. Job satisfaction. New York, NY: Harper; 1935.

[31] Spector PE. Measurement of human service staff satisfaction: development of the job satisfaction survey. Am J Community Psychol. 1985;13:693–713. doi: 10.1007/bf00929796 PubMed PMID: 4083275. 4083275

[32] Cumbey DA, Alexander JW. The relationship of job satisfaction with organizational variables in public health nursing. J Nurs Admin. 1998;28:39–46. doi: 10.1097/00005110-199805000-00007 9601492

[33] Nelson DL, Quick JC. Organizational behavior: science, the real world, and you. 8th ed. Boston (MA): Cengage Learning; 2013.

[34] Mari M, Alloubani A, Alzaatreh M, et al. International nursing: job satisfaction among critical care nurses in a governmental hospital in Saudi Arabia. Nurs Adm Q. 2018;42:E1–E9. doi: 10.1097/NAQ.0000000000000304 29870496

[35] Al-Dossary R, Vail J, MacFarlane F. Job satisfaction of nurses in a Saudi Arabian university teaching hospital: a cross-sectional study. Int Nurs Rev. 2012;59:424–430. doi: 10.1111/j.1466-7657.2012.00978.x 22897196

[36] Alasmari HA, Douglas C. Job satisfaction and intention to leave among critical care nurses in Saudi Arabia. Middle East J Nurs. 2012;6:3–12.

[37] Alharbi J, Wilson R, Woods C, et al. The factors influencing burnout and job satisfaction among critical care nurses: a study of Saudi critical care nurses. J Nurs Manag. 2016;24:708–717. doi: 10.1111/jonm.12386 27189515

[38] Muhawish H, Salem OA, Baker OG. Job related stressors and job satisfaction among multicultural nursing workforce. Middle East J Nurs. 2019;13:3–16. doi: 10.5742/mejn.2019.93635

[39] Ibrahim NK, Alzahrani NA, Batwie AA, et al. Quality of life, job satisfaction and their related factors among nurses working in king Abdulaziz. Jeddah, Saudi Arabia: University Hospital; 2016. p. 486–498. 10.1080/10376178.2016.1224123 27586128

[40] Alfuraih AM, Alsaadi MJ, Aldhebaib AM. Job satisfaction of radiographers in Saudi Arabia. Radiol Technol. 2022;93:268–277. PubMed PMID: 35017270. 35017270

[41] Alqarni T, Alghamdi A, Alzahrani A, et al. Prevalence of stress, burnout, and job satisfaction among mental healthcare professionals in Jeddah, Saudi Arabia. PLOS ONE. 2022;17:e0267578. doi: 10.1371/journal.pone.0267578 35476815 PMC9045659

[42] Kader N, Elhusein B, Elhassan NM, et al. Burnout and job satisfaction among psychiatrists in the mental health service, Hamad medical corporation, Qatar. Asian J Psychiatr. 2021;58:102619. doi: 10.1016/j.ajp.2021.102619 33657445

[43] AlJumail E, Rabbani U. Job satisfaction among primary health care workers in Buraidah, Qassim, Saudi Arabia. World Fam Med. 2021;19:27–33. doi: 10.5742/mewfm.2021.94173

[44] Halawani LA, Halawani MA, Beyari GM. Job satisfaction among Saudi healthcare workers and its impact on the quality of health services. J Fam Med Prim Care. 2021;10:1873–1881. doi: 10.4103/jfmpc.jfmpc_2236_20 PMC8208218 34195119

[45] Alghamdi NG, Khan K. Job satisfaction and organizational commitment of paramedics - a case study of Saudi Arabia. J Comput Theor Nanosci. 2018;15:1283–1290. doi: 10.1166/jctn.2018.7304

[46] Baker OG, Alshehri BD. The relationship between job stress and job satisfaction among Saudi nurses: a cross-sectional study. Nurse Media J Nurs. 2021;10:292–305. doi: 10.14710/NMJN.V10I3.32767

[47] Tariah HA, Nafai S, Alanazi AA, et al. Job satisfaction among occupational therapists working in Riyadh, Saudi Arabia. Work. 2022;72:315–322. doi: 10.3233/WOR-210034 35431212

[48] Al Maqbali MA. Job satisfaction of nurses in a regional hospital in Oman: a cross-sectional survey. J Nurs Res. 2015;23:206–216. doi: 10.1097/jnr.0000000000000081 25875103

[49] Benslimane N, Khalifa M. Evaluating pharmacists’ motivation and job satisfaction factors in Saudi hospitals. Jeddah, Saudi Arabia: King Faisal Specialist Hospital and Research Center; 2016. p. 201–204. 27350504

[50] Yasin YM, Al-Hamad A, Bélanger CH, et al. Expatriate health professionals in the Saudi Arabia private sector. Br J Health Care Manag. 2017;23:176–185. doi: 10.12968/bjhc.2017.23.4.176

[51] Billah SMB, Saquib N, Zaghloul MS, et al. Unique expatriate factors associated with job dissatisfaction among nurses. Int Nurs Rev. 2021;68:358–364. doi: 10.1111/inr.12643 33165919

[52] Al-Haroon HI, Al-Qahtani MF. The demographic predictors of job satisfaction among the nurses of a major public hospital in KSA. J Taibah Univ Med Sci. 2020;15:32–38. doi: 10.1016/j.jtumed.2019.11.003 32110180 PMC7033398

[53] Parveen M, Maimani K, Kassim NM. A comparative study on job satisfaction between registered nurses and other qualified healthcare professionals. Int J Healthc Manag. 2017;10:238–242. doi: 10.1080/20479700.2016.1265781

[54] Almansour H. Factors influencing job satisfaction among recently qualified resident doctors: a qualitative study. Asia Pac J Health Manag. 2021;16:62–69. doi: 10.24083/apjhm.v16i4.689

[55] Baghdadi LR, Baghdadi RR, Kamal RS, et al. Physicians’ job satisfaction, ethics and burnout in Makkah, Saudi Arabia. J Pak Med Assoc. 2020;70:2383–2389. doi: 10.47391/JPMA.401 33475548

[56] Alrawahi S, Sellgren SF, Alwahaibi N, et al. Factors affecting job satisfaction among medical laboratory technologists in University Hospital, Oman: an exploratory study. Int J Health Plann Manage. 2019;34:e763–e75. doi: 10.1002/hpm.2689 30378717

[57] Alrawahi S, Sellgren SF, Altouby S, et al. The application of Herzberg’s two-factor theory of motivation to job satisfaction in clinical laboratories in Omani hospitals. Heliyon. 2020;6:e04829. doi: 10.1016/j.heliyon.2020.e04829 32954029 PMC7486437

[58] Slimane NSB. Motivation and job satisfaction of pharmacists in four hospitals in Saudi Arabia. J Health Manag. 2017;19:39–72. doi: 10.1177/0972063416682559

[59] Assiry AA, Alnemari A, Adil AH, et al. Extensive evaluation of the overall workplace experience and job satisfaction of dentists in Saudi Arabia. Biomed Res Int. 2022;2022:4968489. doi: 10.1155/2022/4968489 35036434 PMC8759891

[60] Alqahtani ND, Alshehry K, Alateeq S, et al. An assessment of job satisfaction: a cross-sectional study among orthodontists of Saudi Arabia. J Orthod Sci. 2018;7:23–30. doi: 10.4103/jos.JOS_77_17 29765916 PMC5952236

[61] Aldrees T, Al-Eissa S, Badri M, et al. Physician job satisfaction in Saudi Arabia: insights from a tertiary hospital survey. Ann Saudi Med. 2015;35:210–213. doi: 10.5144/0256-4947.2015.210 26409795 PMC6074462

[62] Hamasha AA, Alturki A, Alghofaili N, et al. Predictors and level of job satisfaction among the dental workforce in national guard health affairs. J Int Soc Prev Community Dent. 2019;9:89–93. doi: 10.4103/jispcd.JISPCD_418_18 30923700 PMC6402258

[63] Al Jazairy YH, Halawany HS, Al Hussainan N, et al. Factors affecting job satisfaction and their correlation with educational standards among dental assistants. Ind Health. 2014;52:324–333. doi: 10.2486/indhealth.2014-0005 24747371 PMC4243018

[64] Bahnassy AA, Saeed AA, Al Kadhi Y, et al. Physicians’ job satisfaction and its correlates in a tertiary medical care center, Riyadh, Saudi Arabia. Saudi J Med Med Sci. 2016;4:112–117. doi: 10.4103/1658-631x.178343 Epub 20160309. PubMed PMID: 30787709. 30787709 PMC6298327

[65] Alelyani M, Alqahtani M, Khalid Y, et al. Job satisfaction among radiologic technologists at hospitals in Saudi Arabia’s southern region: a cross-sectional study. Biosci Res. 2020;17:1659–1666.

[66] Shubayr N, Faraj H, Hurbush M, et al. Assessment of job satisfaction, lifestyle behaviors, and occupational burnout symptoms during the COVID-19 pandemic among radiologic technologists in Saudi Arabia. Radiography. 2022;28:1087–1092. doi: 10.1016/j.radi.2022.07.015 36030598 PMC9414262

[67] Suleiman S, Adam S. Job satisfaction among nurses working at primary health center in Ras Al Khaimah, United Arab Emirates. Int J Nurs Edu. 2020;12:65–67. doi: 10.5958/0974-9357.2020.00014.8

[68] Almansour H, Gobbi M, Prichard J. Home and expatriate nurses’ perceptions of job satisfaction: qualitative findings. Int Nurs Rev. 2022;69:125–131. doi: 10.1111/inr.12699 34043818

[69] Anzar W, Qureshi A, Afaq A, et al. Analysis of occupational stress, burnout, and job satisfaction among dental practitioners. Work. 2022;72:323–331. doi: 10.3233/WOR-210555 35431216

[70] Almansour H, Gobbi M, Prichard J, et al. The association between nationality and nurse job satisfaction in Saudi Arabian hospitals. Int Nurs Rev. 2020;67:420–426. doi: 10.1111/inr.12613 32700371

[71] Banaser M, Ghulman‬ F, Almakhalas H, et al. Nurses’ job satisfaction during the mass gathering of the Hajj 2018 in Saudi Arabia. Int Nurs Rev. 2020;67:372–379. doi: 10.1111/inr.12590 32441322

[72] Al-Ghareeb HY, Al-Wateyan RA. Job satisfaction in PHC Kuwait. World Fam Med. 2019;17:4–15. doi: 10.5742/mewfm.2019.93640

[73] Alharbi AA, Dahinten VS, MacPhee M. The relationships between nurses’ work environments and emotional exhaustion, job satisfaction, and intent to leave among nurses in Saudi Arabia. J Adv Nurs. 2020;76:3026–3038. doi: 10.1111/jan.14512 32924146

[74] Al-Buainain FS, Alzarouni AA, Alshamsi HA, et al. Job satisfaction of UA.E. dental practitioners. Eur J Dent. 2019;13:354–360. doi: 10.1055/s-0039-1700186 31795001 PMC6890485

[75] Al Sabei SD, Labrague LJ, Al-Rawajfah O, et al. Relationship between interprofessional teamwork and nurses’ intent to leave work: the mediating role of job satisfaction and burnout. Nurs Forum. 2022;57:568–576. doi: 10.1111/nuf.12706 35152423

[76] Alfaleh A, Alkattan A, Alageel A, et al. Onsite versus remote working: the impact on satisfaction, productivity, and performance of medical call center workers. Inquiry. 2021;58. doi: 10.1177/00469580211056041 PMC8640291 34825844

[77] AlEisa E, Tse C, Alkassabi O, et al. Predictors of global job satisfaction among Saudi physiotherapists: a descriptive study. Ann Saudi Med. 2015;35:46–50. doi: 10.5144/0256-4947.2015.46 26142938 PMC6152550

[78] Falatah R, Almuqati J, Almuqati H, et al. Linking nurses’ job security to job satisfaction and turnover intention during reform and privatization: a cross-sectional survey. J Nurs Manag. 2021;29:1578–1586. doi: 10.1111/jonm.13279 33502052

[79] Alotaibi J, Paliadelis PS, Valenzuela FR. Factors that affect the job satisfaction of Saudi Arabian nurses. J Nurs Manag. 2016;24:275–282. doi: 10.1111/jonm.12327 26260125

[80] Brant JM, Fink RM, Thompson C, et al. Global survey of the roles, satisfaction, and barriers of home health care nurses on the provision of palliative care. J Palliat Med. 2019;22:945–960. doi: 10.1089/jpm.2018.0566 31380727

[81] Aldaiji L, Al-Jedai A, Alamri A, et al. Effect of occupational stress on pharmacists’ job satisfaction in Saudi Arabia. Healthcare. 2022;10. doi: 10.3390/healthcare10081441 PMC9408447 36011097

[82] Albashayreh A, Al Sabei SD, Al-Rawajfah OM, et al. Healthy work environments are critical for nurse job satisfaction: implications for Oman. Int Nurs Rev. 2019;66:389–395. doi: 10.1111/inr.12529 31206654

[83] Alshahrani FMM, Baig LA. Effect of leadership styles on job satisfaction among critical care nurses in Aseer, Saudi Arabia. J Coll Physicians Surg Pak. 2016;26:366–370. PubMed PMID: 27225139. 27225139

[84] Al Sabei SD, Labrague LJ, Miner Ross A, et al. Nursing work environment, turnover intention, job burnout, and quality of care: the moderating role of job satisfaction. J Nurs Scholarsh. 2020;52:95–104. doi: 10.1111/jnu.12528 31692251

[85] Falatah R, Conway E. Linking relational coordination to nurses’ job satisfaction, affective commitment and turnover intention in Saudi Arabia. J Nurs Manag. 2019;27:715–721. doi: 10.1111/jonm.12735 30449053

[86] Alenazy FS, Dettrick Z, Keogh S. The relationship between practice environment, job satisfaction and intention to leave in critical care nurses. Nurs Criti Care. 2023;28:167–176. doi: 10.1111/nicc.12737 34882918

[87] Parveen M, Maimani K, Kassim NM. Quality of work life: the determinants of job satisfaction and job retention among RNs and OHPs. Int J Qual Res. 2017;11:173–194. doi: 10.18421/IJQR11.01-11

[88] Amer YS, Al Nemri A, Osman ME, et al. Perception, attitude, and satisfaction of paediatric physicians and nurses towards clinical practice guidelines at a university teaching hospital. J Eval Clin Pract. 2019;25:543–549. doi: 10.1111/jep.12923 29611621

[89] Mirza AA, Badrek-Amoudi AH, Farooq MU, et al. Job satisfaction amongst surgical healthcare professionals during Hajj and non-Hajj periods: an analytical multi-center cross-sectional study in the holy city of Makkah, Saudi Arabia. JPMA J Pak Med Assoc. 2020;70:1371–1375. doi: 10.5455/JPMA.28809 32794488

[90] Al-Muallem N, Al-Surimi KM. Job satisfaction, work commitment and intention to leave among pharmacists: a cross-sectional study. BMJ Open. 2019;9:e024448. doi: 10.1136/bmjopen-2018-024448 PMC6773423 31558448

[91] Yehya A, Sankaranarayanan A, Alkhal A, et al. Job satisfaction and stress among healthcare workers in public hospitals in Qatar. Arch Environ Occup Health. 2020;75:10–17. doi: 10.1080/19338244.2018.1531817 30449263

[92] Alzailai N, Barriball L, Xyrichis A. Burnout and job satisfaction among critical care nurses in Saudi Arabia and their contributing factors: a scoping review. Nurs Open. 2021;8:2331–2344. doi: 10.1002/nop2.843 33760366 PMC8363385

[93] Alghamdi MG, Topp R, AlYami MS. The effect of gender on transformational leadership and job satisfaction among Saudi nurses. J Adv Nurs. 2018;74:119–127. doi: 10.1111/jan.13385 28714146

[94] Abualrub RF, Alghamdi MG. The impact of leadership styles on nurses’ satisfaction and intention to stay among Saudi nurses. J Nurs Manag. 2012;20:668–678. doi: 10.1111/j.1365-2834.2011.01320.x 22823223

[95] Hussain MK, Khayat RAM. The impact of transformational leadership on job satisfaction and organisational commitment among hospital staff: a systematic review. J Health Manag. 2021;23:614–630. doi: 10.1177/09720634211050463

[96] Alqahtani AMA, Nahar S, Almosa K, et al. Leadership styles and job satisfaction among healthcare providers in primary health care centers. World Fam Med. 2021;19:102–112. doi: 10.5742/mewfm.2021.94013

[97] Saleh U, O’Connor T, Al-Subhi H, et al. The impact of nurse managers’ leadership styles on ward staff. Br J Nurs. 2018;27:197–203. doi: 10.12968/bjon.2018.27.4.197 29457941

[98] Devreux I, Jacquerye A, Kittel F, et al. Determinants of rehabilitation services staffs’ job satisfaction (by effort reward imbalance) and variations in teaching, profit making and non profit hospitals. Res J Med Sci. 2012;6:154–158. doi: 10.3923/rjmsci.2012.154.158

[99] Devreux I, Jacquerye A, Kittel F, et al. Measurement of rehabilitation services staffs’ job satisfaction using the effort reward imbalance model in Saudi Arabia. Res J Med Sci. 2012;6:87–92. doi: 10.3923/rjmsci.2012.87.92

[100] Jradi H, Alanazi H, Mohammad Y. Psychosocial and occupational factors associated with low back pain among nurses in Saudi Arabia. J Occup Health. 2020;62:e12126. doi: 10.1002/1348-9585.12126 32515887 PMC7229531

[101] Alharthi H, Youssef A, Radwan S, et al. Physician satisfaction with electronic medical records in a major Saudi government hospital. J Taibah Univ Med Sci. 2014;9:213–218. doi: 10.1016/j.jtumed.2014.01.004

[102] Alfayad Z, Arif LSM. Employee voice and job satisfaction: an application of Herzberg two-factor theory. Int Rev Manag Market. 2017;7:150–156.

[103] Herzberg F, Mausner B, Snyderman BB. The motivation to work. (NB), N.J. U.S.A: Transaction Publishers; 1993.

[104] Miljković S. Motivation of employees and behavior modification in health care organizations. Acta Medica Medianae. 2007;46:53–62.

[105] Fugar FD. Frederick Herzberg’s motivation-hygiene theory revisited: the concept and its applicability to clergy (A study of fulltime stipendiary clergy of the global evangelical church, Ghana. J Sci Technol (Ghana). 2007;27:119–130. doi: 10.4314/just.v27i1.33031

[106] Lee SE, MacPhee M, Dahinten VS. Factors related to perioperative nurses’ job satisfaction and intention to leave. Jpn J Nurs Sci. 2020;17:e12263. doi: 10.1111/jjns.12263 PubMed PMID: 31161733. 31161733

[107] Tasneem S, Cagatan AS, Avci MZ, et al. Job satisfaction of health service providers working in a public tertiary care hospital of Pakistan. Open Public Health J. 2018;11:17–27. doi: 10.2174/1874944501811010017

[108] Eker L, Tüzün EH, Daskapan A, et al. Predictors of job satisfaction among physiotherapists in Turkey. J Occup Health. 2004;46:500–505. doi: 10.1539/joh.46.500 15613776

[109] McAuliffe E, Daly M, Kamwendo F, et al. The critical role of supervision in retaining staff in obstetric services: a three country study. PLOS ONE. 2013;8:e58415. doi: 10.1371/journal.pone.0058415 23555581 PMC3605440

[110] Martinko MJ, Gardner WL. Learned helplessness: an alternative explanation for performance deficits. Acad Manage Rev. 1982;7:195–204. doi: 10.2307/257297

[111] Jones ML. Action regulation theory. In: Seel N, editor. Encyclopedia of the sciences of learning. Boston, MA: Springer US; 2012. p. 66–69.

[112] Alakhras M, Al-Mousa D, Lewis S. Assessment and correlation between job satisfaction and burnout among radiographers. Radiography. 2022;28:283–287. doi: 10.1016/j.radi.2021.11.003 34838438

[113] Vanckaviciene A, Navickiene R, Viliusiene I, et al., editors. Radiographers’ job satisfaction: cross-sectional survey in Lithuania 2018. European Congress of Radiology-ECR; 2018. doi:10.1594/ecr2018/C-2223

[114] Tarcan M, Hikmet N, Schooley B, et al. An analysis of the relationship between burnout, socio-demographic and workplace factors and job satisfaction among emergency department health professionals. Appl Nurs Res. 2017;34:40–47. doi: 10.1016/j.apnr.2017.02.011 28342622

[115] Vermeir P, Degroote S, Vandijck D, et al. Job satisfaction in relation to communication in health care among nurses: a narrative review and practical recommendations. Sage Open. 2017;7:2158244017711486. doi: 10.1177/2158244017711486

[116] Kifle T, Kler P, Shankar S. Are women really that happy at work? Australian evidence on the ‘contented female’. Appl Econ. 2014;46:686–697. doi: 10.1080/00036846.2013.851781

[117] Coomber B, Barriball KL. Impact of job satisfaction components on intent to leave and turnover for hospital-based nurses: a review of the research literature. J Nurs Stud. 2007;44:297–314. doi: 10.1016/j.ijnurstu.2006.02.004 16631760

[118] Bass BM, Avolio BJ, Jung DI, et al. Predicting unit performance by assessing transformational and transactional leadership. J Appl Psychol. 2003;88:207. doi: 10.1037/0021-9010.88.2.207 12731705

[119] Guan X, Ni B, Zhang J, et al. Association between physicians’ workload and prescribing quality in one tertiary hospital in China. J Patient Saf. 2021;17:e1860–e5. doi: 10.1097/PTS.0000000000000753 PubMed PMID: 32773646. 32773646

[120] Mere RA, Simbeni TV, Mathibe M, et al. Job satisfaction among health professionals in a district of North West province, South Africa. Health SA. 2023;28. doi: 10.4102/hsag.v28i0.2234 PubMed PMID: 37292237; PubMed Central PMCID: PMC10244876. PMC10244876 37292237

