# Supplementary material for: A systematic review of the determinants of job satisfaction in healthcare workers in health facilities in Gulf Cooperation Council countries
Source: Glob Health Action. 2025 Apr 4;18(1):2479910. doi: 10.1080/16549716.2025.2479910 (PMC11980202; doi:10.1080/16549716.2025.2479910)
Supplement: Supplemental Material [file ZGHA_A_2479910_SM6402.docx]

**Appendix 1 (page 3 line 205 for your help)**

Search terms in different databases

| Database | Search term |
| --- | --- |
| Web of Science | (ALL=((health workers) OR (health personnel)) AND ALL=(job satisfaction)) AND (PY==("2012" OR "2013" OR "2014" OR "2015" OR "2016" OR "2017" OR "2018" OR "2019" OR "2020" OR "2021" OR "2022") AND LA==("ENGLISH") AND CU==("SAUDI ARABIA" OR "QATAR" OR "OMAN" OR "U ARAB EMIRATES" OR "KUWAIT" OR "BAHRAIN")) |
| Scopus | (TITLE-ABS-KEY(job AND satisfaction) AND TITLE-ABS-KEY(health workers) OR TITLE-ABS-KEY(health personnel) OR ALL(healthcare AND workers OR nurses OR medical AND workers OR healthcare AND professionals OR doctors)) AND ( LIMIT-TO ( LANGUAGE,"English" ) ) AND ( LIMIT-TO ( PUBYEAR,2022) OR LIMIT-TO ( PUBYEAR,2021) OR LIMIT-TO ( PUBYEAR,2020) OR LIMIT-TO ( PUBYEAR,2019) OR LIMIT-TO ( PUBYEAR,2018) OR LIMIT-TO ( PUBYEAR,2017) OR LIMIT-TO ( PUBYEAR,2016) OR LIMIT-TO ( PUBYEAR,2015) OR LIMIT-TO ( PUBYEAR,2014) OR LIMIT-TO ( PUBYEAR,2013) OR LIMIT-TO ( PUBYEAR,2012) ) AND ( LIMIT-TO ( AFFILCOUNTRY,"Saudi Arabia" ) OR LIMIT-TO ( AFFILCOUNTRY,"United Arab Emirates" ) OR LIMIT-TO ( AFFILCOUNTRY,"Oman" ) OR LIMIT-TO ( AFFILCOUNTRY,"Qatar" ) OR LIMIT-TO ( AFFILCOUNTRY,"Bahrain" ) OR LIMIT-TO ( AFFILCOUNTRY,"Kuwait" ) ) |
| CINAHL | ( job satisfaction or work satisfaction or employee satisfaction ) AND ( healthcare workers or nurses or medical workers or healthcare professionals or doctors ) AND ( saudi arabia or kingdom of saudi arabia or ksa or uae or united arab emirate or qatar or bahrain or kuwait or oman ). Date adjusted manually to be between 2012-2022. |
| Cochrane | (health personnel):ti,ab,kw AND (job satisfaction):ti,ab,kw AND ("Saudi Arabia" OR "Qatar" OR "Kuwait" OR "Emirate" OR "Bahrain" OR "Oman"):ti,ab,kw" with Publication Year from 2012 to 2022 |

**Appendix 2 (page 4 line 248 for your help)**

| **Ser** | **Author(s), Citation** | **Study design** | **Aim of the study** | **Study setting** | | **Study period** | **Population of the study** | **Sampling** | | | **Data collection** | | | **Study findings** | | **Conclusion of the study** | |
| --- | --- | --- | --- | --- | --- | --- | --- | --- | --- | --- | --- | --- | --- | --- | --- | --- | --- |
|  |  |  |  | **Country** | **Place** |  |  | **Sample size** | **Sampling technique** | **Tool of the study** | | **Data collection technique** |  | |  | |  |
| 1 | Mari M, Alloubani A, Alzaatreh M, Abunab H, Gonzales A, Almatari M. International urnsing: job satisfaction among critical care nurses in a governmental hospital in Saudi Arabia. Nurs Adm Q. 2018;42(3):E1-E9.. | Cross-sectional, quantitative | To measure factors that affect job satisfaction among critical care nurses at King Khalid Hospital in Saudi Arabia. | Saudi Arabia | Main government hospital | May-July 2015 | ICU nurses | 190 | convenient sampling | job satisfaction survey (JSS, Spector 1997) | | Not reported | The staff nurses were neither satisfied nor dissatisfied with their work. The majority of participating nurses were moderately satisfied with their relationships with coworkers (mean = 4.24, standard deviation [SD] = 1.66) and the nature of their jobs (mean = 4.13, SD = 0.74). Their lowest satisfaction scores were related to communication (mean = 3.42, SD = 0.82), followed by pay (mean = 3.7, SD = 0.65) and supervision (mean = 3.75, SD = 0.55). | | Job satisfaction of staff nurses is marginal. The study found an ambivalent level of satisfaction among the nurses, which calls for adopting new strategies to increase nurses' level of job satisfaction and work engagement. | |  |
| 2 | Tariah HA, Nafai S, Alanazi AA, Alobathani H, Alotaibi LM, Almutairi SS. Job satisfaction among occupational therapists working in Riyadh, Saudi Arabia. Work. 2022;72(1):315-22. | Cross-sectional, descriptive study | To identify the level of job satisfaction of occupational therapists. | Saudi Arabia | Medical cities | Not mentioned | occupational therapists | 48 | Non-probability purposive sampling | A validated job satisfaction questionnaire. The Measure of Job Satisfaction "MJS" questionnaire. (Tayron & Wade, 1993). For content validation, the MJS measures five work factors: personal satisfaction, workload, professional support, salary, and prospects and training. [30] (44 items) | | an electronic version using Google forms | Most occupational therapists reported a high overall satisfaction level. They showed satisfaction with coworkers, care provided to clients, and autonomy. However, they showed dissatisfaction with salary compared to the work efforts, lack of reimbursement for continuing education, and lack of opportunities for career advancement. | | The overall level of satisfaction of the occupational therapists was high. | |  |
| 3 | Bahnassy AA, Saeed AA, Al Kadhi Y, Al-Harbi J. Physicians' Job Satisfaction and its Correlates in a Tertiary Medical Care Center, Riyadh, Saudi Arabia. Saudi J Med Med Sci. 2016;4(2):112-7. | Cross-sectional study | To measure the degree of job satisfaction among physicians working in a Tertiary Care Hospital and to identify background and work environment characteristics that affect overall and differential job satisfaction. | Saudi Arabia | Tertiary Care Hospital | Not mentioned | physicians | 217 | A stratified random sample | A self-administered questionnaire. It contained demographic variables, job rank, and 40 statements on a 5-point Likert scale to measure physician satisfaction. (unique) | | Interview | The study results showed a significant relationship between the physicians’ satisfaction scores and work environment policies, such as duration of vacation leave, sick leave policy, health coverage to the participant and his family, retirement plan benefits (for Saudis), disability benefits, and the overall benefits package. The physicians’ stipend was significantly related to the level of job satisfaction. | | The working environment and policies play essential roles in physician satisfaction of its physicians. Boosting physician satisfaction is vital for a Tertiary Care Centre’s success and high-quality patient services. | |  |
| 4 | Al-Dossary R, Vail J, MacFarlane F. Job satisfaction of nurses in a Saudi Arabian university teaching hospital: A cross-sectional study. Int Nurs Rev. 2012;59(3):424-30. | Cross-sectional, quantitative | To measure nurses' job satisfaction in Saudi Arabia in a university teaching hospital and determine the influencing factors | Saudi Arabia | University Teaching Hospital | Mar-May 2008 | nurses | 189 | systematic sample | A self-administered questionnaire. The job satisfaction survey (JSS; Spector 1997) was used in this research. | | Interview | Nurses were neither satisfied nor dissatisfied with their jobs. They are satisfied with supervision, coworkers, and the nature of work. However, they were unsatisfied with subscale things such as pay, fringe benefits, contingent rewards, and operating conditions. | | There is a need to increase nurses' salaries and bonuses for extra duties. More training programs and further education also should be encouraged for all nurses. | |  |
| 5 | Alharbi AA, Dahinten VS, MacPhee M. The relationships between nurses' work environments and emotional exhaustion, job satisfaction, and intent to leave among nurses in Saudi Arabia. J Adv Nurs. 2020;76(11):3026-38 | A descriptive correlational study with cross-sectional data. | To examine relationships between components of nurses' work environments and emotional exhaustion, job satisfaction, and intent to leave among nurses in Saudi Arabia. | Saudi Arabia | Tertiary Hospital | 7w-1 Aug2017 | Registered Nurses | 496 | convenience sample | RN4CAST survey, BC Nurses' Workload Impact Study, PES-NWI, Emotional Exhaustion subscale of the Maslach Burnout Inventory Human Service Scale, job satisfaction, Nurses' intent to leave, and Nurses' Demographic Characteristics. Job Satisfaction was measured with a single item, asking nurses to identify their overall satisfaction with their current jobs. | | Online survey | Nurse participation in hospital affairs was uniquely associated with all three nurse outcomes. In contrast, staffing and resource adequacy were associated with emotional exhaustion and job satisfaction but not intent to leave. These two variables were also the components of the nursing practice environment that received the lowest ratings. Nurse manager ability, leadership and support of nurses, and nurse-physician relationships were associated with job satisfaction only. A nursing foundation for quality of care was not uniquely associated with any of the three outcomes. Finally, nurses’ emotional exhaustion and job satisfaction fully mediated the relationship between nurses’ participation in hospital affairs and their intent to leave. | | Magnet-like work environments in Saudi Arabia are critical to recruiting and retaining nurses in a country with critical nursing shortages. | |  |
| 6 | Assiry AA, Alnemari A, Adil AH, Karobari MI, Sayed FR, Marya A, et al. Extensive evaluation of the overall workplace experience and job satisfaction of dentists in Saudi Arabia. 2022. Biomed Res Int. 2022;4968489 | Cross-sectional | To determine the factors influencing the job satisfaction level among dentists and evaluate how personal and professional characteristics influence overall job satisfaction. | Saudi Arabia | Faculty of Dentistry, Najran University | One Jan-30 Apr2021 | dentists | 155 | convenience sample | A structured self-administered questionnaire: information on personal characteristics and job satisfaction. The German-validated questionnaire version has a 10-point Warr-Cook-Wall (WCW) scale developed by Warr et al. in 1979. | | Interview | Dentists were more satisfied with colleagues and fellow workers. The relationship between the years of practice was highly significant. However, they were dissatisfied with their income. | | The fellow and colleague workers’ dimensions showed a higher percentage of satisfaction. Income was the aspect with which the dentists showed extreme dissatisfaction. | |  |
| 7 | Albashayreh A, Al Sabei SD, Al-Rawajfah OM, Al-Awaisi H. Healthy work environments are critical for nurse job satisfaction: implications for Oman. Int Nurs Rev. 2019;66(3):389-95. | Cross-sectional descriptive design | To examine the level and variability of nurses’ work environment and job satisfaction and explore how their characteristics and work environment influence nurses’ job satisfaction. | Oman | Tertiary Hospital | March- Nov 2016 | nurses | 454 | convenient sample | A self-administered questionnaire includes background variables, nurse work environment (NEW), and nurse job satisfaction (NJS). Job Satisfaction Scale (Lynch et al. 2005) 6 items | | electronic invitation letter | The work environment was favorable, and it positively correlated with job satisfaction. Nationality, education, hospital type, staffing, resources, and participation in hospital affairs were significant predictors of nurse job satisfaction. | | Enhancing nurse participation in hospital affairs and providing adequate staffing and resources are central means of establishing healthy work environments, which is an auspicious, cost-effective strategy for satisfying and retaining nurses. | |  |
| 8 | Al Maqbali MA. Job satisfaction of nurses in a regional hospital in Oman: A cross-sectional survey. J Nurs Res. 2015;23(3):206-16. | Cross-sectional | To measure the job satisfaction of nurses working at a regional hospital in Oman and determine the factors that most significantly influence this satisfaction. | Oman | Regional Hospital | May, 2013 | nurses | 155 | stratified random sampling | A self-administered questionnaire with close-ended questions. McCloskey/Mueller Satisfaction Scale (MMSS; Mueller & McCloskey, 1990) 31 items (8subscales) | | Interview | participants had a moderate level of job satisfaction. “Interaction with coworkers" and "extrinsic reward" earned the highest and lowest job satisfaction ratings on the McCloskey/Muller Satisfaction Scale subscale. The results showed that age, work shift, and nationality significantly affected overall job satisfaction. | | Professional opportunity and extrinsic rewards are two important factors that affect the job satisfaction of nurses in Oman. | |  |
| 9 | Alfaleh A, Alkattan A, Alageel A, Salah M, Almutairi M, Sagor K, et al. Onsite versus remote working: the impact on satisfaction, productivity, and performance of medical call center workers. Inquiry. 2021;58:469580211056041 | Cross-sectional | To determine the role of remote call center working on agents ' satisfaction compared to onsite working. | Saudi Arabia | Government medical call center | Dec 2020-Apr2021 | Physicians | 124 | All agents working in a governmental medical call center (purposive sample) | This questionnaire comprised six sections: socio-demographic data, data about physician’s satisfaction (job nature, supervisor support, job autonomy, and job productivity and performance), and an open-ended question regarding the physicians' suggestions for improving their satisfaction and performance (unique). | | Distributed by hand and online | Onsite agents were more satisfied with the nature of their jobs, supervisor support, productivity, and performance than remote agents. However, onsite agents were less satisfied regarding job autonomy than remote physicians. | | Onsite agents were significantly more satisfied in most investigated aspects. Besides, part-time agents were satisfied more than full-time agents, and the difference between them was significant. | |  |
| 10 | Yehya A, Sankaranarayanan A, Alkhal A, Alnoimi H, Almeer N, Khan A, et al. Job satisfaction and stress among healthcare workers in public hospitals in Qatar. Arch Environ Occup Health. 2020;75(1):10-7. | Cross-sectional | To assess the prevalence and predictor of job dissatisfaction among our diverse employees, most of whom were full-time employees | Qatar | Hamad Medical Corporation, public healthcare system. | two months | physician, nurses, senior administrative staff, both medical and healthcare managers. (Unique) | 1260 | All staff in the corporation | sociodemographic data followed by the National Institute for Occupational Safety and Health (NIOSH) generic Job Stress questionnaire. (Unique) | | The link was sent by email | Role ambiguity, conflict, skill underutilization, and workload were associated with job dissatisfaction. Role and job future ambiguity were significantly associated with depression. | | The study showed that nearly 42% of the health workers who responded to the survey were dissatisfied with their jobs and that the prevalence of depression among healthcare workers was higher than that reported in the general population. | |  |
|  | Alqahtani ND, Alshehry K, Alateeq S, Alturki H, Albarakati S, Asiry MA, et al. An assessment of job satisfaction: A cross-sectional study among orthodontists of Saudi Arabia. J Orthod Sci. 2018;7(1):23-30. | Cross-sectional | To investigate job satisfaction among professional orthodontists concerning some significant intrinsic and extrinsic factors that generally affect their performance. | Saudi Arabia | College of Dentistry | Not mentioned | orthodontists |  | All orthodontists registered | The job satisfaction survey comprised 20 questions, including intrinsic and extrinsic factors such as age, length of experience, respect, professional time ….......etc. (Unique) | | Survey Monkey link via email | Most orthodontists (80.7%) were satisfied with orthodontics as their profession, irrespective of gender. Only 4.4% showed dissatisfaction, whereas the remaining 14.9% were moderately satisfied with orthodontics as a profession. An in-depth analysis revealed that most respondents (56.2%) showed reservations over having ample time for their family life. 52.8% of the respondents strongly agreed that they were assigned significant paperwork. Likewise, 66.5% of the orthodontists showed moderate dissatisfaction over time adherence by the patients. | | Orthodontics is a field where professionals have passion and ambitions for their professional growth and development. Furthermore, orthodontists rank this field as highly prestigious and high paying. | |  |
| 12 | Anzar W, Qureshi A, Afaq A, Alkahtany MF, Almadi KH, Ben Gassem AA, et al. Analysis of occupational stress, burnout, and job satisfaction among dental practitioners. Work. 2022;72(1):323-31. | An analytical cross-sectional | To investigate dimensions of occupational stress among dental practitioners leading to burnout and to model its causality with job satisfaction. | Saudi Arabia | dental practitioners working in clinical and non-clinical departments of public and private sector universities, as well as those doing private general practice. | Nov2019,5month | dental practitioners | 302 | consecutive sample | A self-administered structured questionnaire based on a work stress questionnaire. The Work Stress Questionnaire (WSQ) included four parts: Burnout Inventory (BI) and Job satisfaction, measured using the Warr-Cook Wall job satisfaction scale. (WCW) | | SPSS | The prevalence of overall occupational stress was high among dental practitioners (83.4%). Occupational stress was greatest in females (55.8%), dental practitioners in the private sector (55.7%), and general dental practitioners (GDPs) (47%). The overall prevalence of job satisfaction among dental professionals was 30%. Occupational stress was significantly associated with burnout and job dissatisfaction in dentistry. Stress levels and working hours were statistically and positively associated, whereas the association between stress levels and job satisfaction was inversely and significantly associated. | | The prevalence of occupational stress among dental practitioners was high, and job satisfaction was moderate. | |  |
| 13 | Alzailai N, Barriball L, Xyrichis A. Burnout and job satisfaction among critical care nurses in Saudi Arabia and their contributing factors: A scoping review. Nursing Open. 2021;8(5):2331-44. | Systematic review | To assess the degree of well-being of nurses working in critical care settings in Saudi Arabia by evaluating their burnout and job satisfaction levels and the factors contributing to them. | Saudi Arabia | Seven databases were searched for published research that examined the levels and factors of burnout and job satisfaction in ICU nurses. | January-Dec2020 | ICU nurses | All available evidence (11 studies) | Seven databases were searched for published research that examined the levels and factors of burnout and job satisfaction in ICU nurses | literature review (PRISMA) (Unique) | | Systematic Review and Meta-Analysis | Intensive care unit nurses in Saudi Arabia are suffering from moderate to high levels of burnout while experiencing only moderate levels of job satisfaction. Three categories of factors were associated with burnout and nursing job satisfaction: intrapersonal, interpersonal, and extra-personal. | | The studies identified a high level of burnout among Saudi ICU nurses, which ranged from moderate to high, with only an average level of job satisfaction. | |  |
| 14 | Alasmari HA, Douglas C. Job Satisfaction and Intention to leave among critical care nurses in Saudi Arabia. Middle East J Nurs. 2012;6(4):3-12. | Cross-sectional | To examine the relationship between registered nurses’(RN)job satisfaction and their intention to leave critical care nursing in Saudi Arabia. | Saudi Arabia | University Hospital | Not mentioned, it said Participants  completed the questionnaires and  returned them in sealed envelopes,  one to three weeks later, to survey  collection boxes in each unit | ICU nurses | 182 | convenience sample | self-administered survey including demographic items and validated measures of job satisfaction and intention to leave. Measure of Job Satisfaction (MJS) is a 38-item scale (Traynor & Wade, 1993). | | SPSS | Demographic variables, including age, parental status, and length of ICU experience, and three job satisfaction subscales, including perceived workload, professional support pay, and prospects for promotion, were significantly associated with the outcome variables. | | This study adds to the existing literature on the relationship between job satisfaction and intention to leave critical care areas among RNs working in Saudi Arabia. It needs management and policy interventions targeting nurses' workloads, professional support, pay, and promotion to improve nurse retention. | |  |
| 15 | Alharbi J, Wilson R, Woods C, Usher K. The factors influencing burnout and job satisfaction among critical care nurses: a study of Saudi critical care nurses. J Nurs Manag. 2016;24(6):708-17 | Cross-sectional | explore the prevalence of burnout and job satisfaction among Saudi national critical care nurses. | Saudi Arabia | Three government Hospitals | 15 Oct-20 Dec 2014 (over 10 weeks) | ICU nurses | 150 | convenience sample | A self-report questionnaire that incorporated demographic variables, the Maslach burnout inventory (MBI), and the job satisfaction survey (JSS) (Spector 1997) 36 items. | | Descriptive/SPSS | Saudi national critical care registered nurses reported moderate to high burnout in emotional exhaustion and depersonalization. Participants also reported a feeling of ambivalence and dissatisfaction with their jobs but were satisfied with the nature of their work. | | Saudi national critical care nurses experience moderate to high burnout and low job satisfaction. Burnout is a predictor of job satisfaction for Saudi national critical care nurses. | |  |
| 16 | Alshahrani FMM, Baig LA. Effect of leadership styles on job satisfaction among critical care nurses in Aseer, Saudi Arabia. J C Physicians Surg Pak. 2016;26(5):366-70.  . | Cross-sectional | To evaluate the effect of transformational and transactional leadership styles of head nurses on the job satisfaction of staff nurses in critical care units (CCU) of a tertiary care hospital. | Saudi Arabia | Tertiary care hospital | July-Dec 2012 | ICU nurses | 89 | convenience sample | multifactor leadership questionnaire and job satisfaction survey with demographics were used. JSS (Spector 1985) | | SPSS | All nurse leaders demonstrated a combination of transactional (TA) and transformational (TF) leadership styles. Nurses working under leaders with a TF style demonstrated significantly higher job satisfaction. The operating conditions were related negatively to the style of leadership. Pay, fringe benefits, and work nature were unrelated to the leadership style. The nurses were moderately satisfied with their work, and 23% of the variation in nurses' job satisfaction could be explained by the head nurse's six leadership facets with the positive effect of professional support, intellectual motivation, management by correction, and their laissez-faire style. | | The study emphasized the importance of the transformational style of head nurses in increasing job satisfaction among nurses’ staff. | |  |
| 17 | Muhawish H, Salem OA, Baker OG. Job related stressors and job satisfaction among multicultural nursing workforce. Middle East J Nurs. 2019;13(2):3-16. | Cross-sectional | To identify the different job-related stressors in hospitals that affect nurses' job satisfaction from a multicultural perspective and examine how these two variables correlated. | Saudi Arabia | University Hospital | Over 6 months | nurses | 150 | convenience sample | Expanded Nursing Stress Scale and the Job Satisfaction Scale (JSS) (Spector, 1985). | | Interview | The most stressful factors included criticism and conflicts, negatively affecting job satisfaction. Studies showed that environmental stressors, like unclear and ambiguous job roles and unequal treatment of nurses, are as significant as internal stress factors, including personality types. Nurses are exposed to inadequate work conditions where they are not appreciated and receive low pay and no benefits. The participants' job satisfaction is relatively low (M = 3.8; SD = 0.43). The results showed that the respondents were particularly dissatisfied with their pay (M = 1.46; SD = 0.63) and fringe benefits (M = 2.14; SD = 0.95). Low job satisfaction results from high stress among respondents and the character of stressors. There is a positive correlation between disagreement in the treatment of patients and demands of work (p = 0.017; p < 0.05); demands of work are positively correlated with decisions under pressure (p = 0.014; < 0.05). | | High stress and workload are disproportionate to the payment provided to nurses. | |  |
| 18 | Aldaiji L, Al-jedai A, Alamri A, Alshehri AM, Alqazlan N, Almogbel Y. Effect of occupational stress on pharmacists’ job satisfaction in Saudi Arabia. Healthcare. 2022;10(8):1441. | cross-sectional | To assess the impact of work stress on job satisfaction among pharmacists in Saudi Arabia. | Saudi Arabia | Academic institutions, hospitals, the drug industry, and conferences. | Aug 2019 to Oct 2020 | Pharmacists | 284 | convenient sample | The surveys were paper-based and self-administered, including four sections: sociodemographic, occupational stress, an expectation assessment, and job satisfaction. ERI scale (16 items) Unique | | Interview | The results of this study indicated that occupational stress and satisfaction negatively influenced pharmacists, whereas confirmation was associated with better satisfaction. Also, there was a positive relationship between pharmacists' job satisfaction and age, male gender, marriage, and higher level of education. Moreover, hospital pharmacists had lower job satisfaction. | | Hospital pharmacists had lower job satisfaction. Pharmacists' job satisfaction may help improve medication safety and ensure an adequate pharmacist workforce. | |  |
| 19 | Benslimane N, Khalifa M. Evaluating pharmacists' motivation and job satisfaction factors in Saudi Hospitals. King Faisal Specialist Hospital and Research Center, Jeddah, Saudi Arabia. Stud Health Technol inform. 2016.226;201-4. | Cross-sectional | To assess the level of job satisfaction and factors that motivate pharmacists in four different hospitals in Saudi Arabia | Saudi Arabia | Four different hospitals | Not mentioned | Pharmacists | 19 pharmacy managers and 71 pharmacists | Not mentioned | Minnesota Satisfaction Questionnaire. | | Interview | Managers must revise their plans and focus on implementing effective motivation and retention strategies. Motivation acts like a psychological power that determines direction and behavior. 62% of pharmacists’ job satisfaction significantly changed from last year. 70% are motivated by a combination of both financial and non-financial incentives. 76% of pharmacists believe financial incentives are essential in motivating and significantly influencing their performance. | | These results show that managers must revise their plans and focus on implementing effective motivation and retention strategies. Identifying the correct motivation factors can lead to continuous improvement and higher competitive advantage. One rapidly growing area is automation, especially in pharmacy technology, such as automated drug dispensing systems. | |  |
| 20 | Yasin YM, Al-Hamad A, Bélanger CH, Boucher A, AbuRubeiha MA. Expatriate health professionals in the Saudi Arabia private sector. Brit J of Healthcare Manag. 2017;23(4):176-85. | Descriptive, exploratory, cross-sectional,  and correlational design | To identify the stressors that affect job satisfaction among different EHPs and the relationship between job satisfaction and turnover intention among EHPs in Saudi private hospitals. | Saudi Arabia | Private Sector in four urban regions | Not mentioned | Expatriate Health Professionals, doctors, nurses, and pharmacists | 204 | convenience sample | Unique (Herzberg) | | Interview | The results showed that EHPs have moderate job satisfaction. | | The results of this study are consistent with Herzberg’s theory; the findings provide no empirical evidence to support the relationship between job satisfaction and turnover intention among EHPs. All the hygiene factors examined in our study were significantly correlated with job satisfaction. | |  |
| 21 | Abualrub RF, Alghamdi MG. The impact of leadership styles on nurses' satisfaction and intention to stay among Saudi nurses. J Nurs Manag. 2012;20(5):668-78.  . | Descriptive correlational design- cross-sectional | To examine the impact of nurse managers' leadership styles on Saudi nurses' job satisfaction and their intention to stay at work. | Saudi Arabia | Six Public Hospitals | June-August 2009 | Saudi registered nurses | 308 | convenience sample | Multifactor leadership questionnaire, job satisfaction survey, McCain's Intent to stay Scale, and the demographic form. Job Satisfaction Survey (Spector 1985) | | Interview | Saud nurses were moderately satisfied with their jobs. In addition, nurses were more satisfied with leaders who demonstrated transformational leadership styles, and those who were more satisfied with their jobs intended to stay at work. The background variables, the transformational and transactional leadership styles, explained 32% of the variation in job satisfaction. | | The result of the study emphasized the importance of transformational leadership, which indicates the need for further attention to training and the development of effective leadership behaviors. | |  |
| 22 | Al-Buainain FS, Alzarouni AA, Alshamsi HA, Arab AH, Bader F, Awad M. Job satisfaction of UAE dental practitioners. Eur J Dent. 2019;13(3):354-60. | Cross-sectional | To assess the overall job satisfaction among working dentists in the UAE and the effect of sociodemographic factors and work environment factors on their level of satisfaction. | United Arab Emirates | private and public sectors | November 2016 - April 2017 | dentists | 197 | Multiple lists of licensed dentists were obtained from various authorities. | Unique | | Interview | The overall job satisfaction of dentists in the UAE is higher than in other countries. The highest satisfaction was related to patient, colleague, and staff relationships. On the other hand, the least satisfaction was linked to the opportunity for part-time work and a benefits package. There were no significant differences between male and female participants regarding all work-related factors apart from autonomy. However, private-sector dentists were more satisfied than the public sector in many work-related factors. | | Various dimensions collectively influence the level of overall job satisfaction. Differences existing between the levels of job satisfaction among private and public sector dentists and between male and female dentists must be addressed to increase job satisfaction among UAE dentists and thus improve the dental care system. | |  |
| 23 | Devreux I, Jacquerye A, Kittel F, Mamdouh K, Al-Awa B. Determinants of rehabilitation services staffs' job satisfaction (by effort reward imbalance) and variations in teaching, profit making and non profit hospitals. Res J Med Sci. 2012;6(4):154-8. | Descriptive statistics and correlations analysis- cross-sectional | To identify the components of job satisfaction based on the effort and reward imbalance model and compare the job satisfaction level of employees working in the different types of healthcare facilities in the Jeddah area. | Saudi Arabia | public and private healthcare settings, ten hospitals | Not mentioned | Therapists and assistants | 166 | random sample | Minnesota Satisfaction questionnaire (Weiss et al., 1967) | | Interview | There is a significant difference in the level of job satisfaction measured by effort and reward imbalance between the therapists and assistant therapists working in teaching, private, and non-profit healthcare facilities. | | Rehabilitation services staff working in teaching and profit-making hospitals generally had a higher effort-reward imbalance ratio with work stress and low satisfaction than nonprofit hospital facilities. Job satisfaction of rehabilitation staff varies per hospital type and the specific mission of the health care facilities. | |  |
| 24 | Devreux I, Jacquerye A, Kittel F, Almazrooa A, Al-Awa B. Measurement of rehabilitation services staffs' job satisfaction using the effort reward imbalance model in Saudi Arabia. Res J Medi Sci. 2012;6(3):87-92. | Cross-sectional | To evaluate the level of work satisfaction of staff working in rehabilitation services based on the Effort-Reward Imbalance Model. | Saudi Arabia | public and private healthcare settings (10 hospitals) | Not mentioned | Therapists and assistants | 166 | random sample | JSS (Spector, 1997) | | Interview | The results indicated a significant correlation between job satisfaction measured by the effort-reward imbalance ratio with the variables of age, nationality, work specialty, educational levels, caseload types, and the number of patients treated daily per therapist. On average, decreased job satisfaction with a significantly higher effort-reward-imbalance ratio in work is expressed by foreign nationals, respiratory therapists, night schedule workers, and therapists with a high workload or adult, geriatric, or inpatient caseloads. | | The effort or workload, the need for professional growth, and financial benefits appear to be essential determinants of job satisfaction and must be considered in the human resources management of rehabilitation services staff. | |  |
| 25 | Labrague LJ, Al Sabei S, Al Rawajfah O, AbuAlRub R, Burney I. Interprofessional collaboration as a mediator in the relationship between nurse work environment, patient safety outcomes and job satisfaction among nurses. J Nurs Manag. 2022;30(1):268-78. | Cross-sectional, descriptive study | To assess the contribution of the work environment to patient safety outcomes and job satisfaction, with interprofessional collaboration as a mediating factor. | Oman | Teaching Hospitals | Not mentioned | clinical nurses | 881 | Not mentioned | . Job satisfaction was measured by a single item (overall, how satisfied are you with your job?) | | Interview | A more favorable nurse work environment enhanced interprofessional collaborations, which increased job satisfaction and nurse-reported care quality and reduced reporting of adverse events. | | The result suggests the importance of enhancing the work environment as a tangible strategy to foster interprofessional collaboration, improve nurses' job satisfaction, and enhance patient safety outcomes. | |  |
| 26 | Al Sabei SD, Labrague LJ, Miner Ross A, Karkada S, Albashayreh A, Al Masroori F, et al. Nursing work environment, turnover intention, job burnout, and quality of care: the moderating role of job satisfaction. J Nurs Scholarsh. 2020;52(1):95-104. | Cross-sectional descriptive study | assess predictors of turnover intention, burnout, and perceived quality of care among nurses working in Oman and examine the potential moderating role of job satisfaction on the relationship between work environment and nurse turnover intention. | Oman | Public Hospital (regional referral hospital) | Not mentioned | nurses | 207 | convenience sample | Job Satisfaction Scale (6 items) (Lynch, Plant, &Ryan, 2005) | | electronic survey | Participation in hospital affairs, a foundation for quality of care, and staffing adequacy were predictors of burnout among nurses and perceived quality of care. Logistic regression analysis revealed that working in a favorable environment was associated with less turnover intention, but only when job satisfaction was high. | | Improving job satisfaction among nurses from different nationalities and educational backgrounds should be part of the quality monitoring strategy. The findings showed that job satisfaction significantly enhances quality-of-care delivery and creates a favorable work environment in healthcare institutions. | |  |
| 27 | Al-Omar HA, Khurshid F, Sayed SK, Alotaibi WH, Almutairi RM, Arafah AM, et al. Job motivation and satisfaction among female pharmacists working in private pharmacy professional sectors in Saudi Arabia. Risk Manag Healthc Policy. 2022;15:1383-94. | Cross-sectional study | To investigate the perceived motivational factors and assess the level of job satisfaction of female pharmacists working in private pharmaceutical sectors. | Saudi Arabia | Private settings | Not mentioned | female pharmacists | 232 | convenience sample | Warr-Cook-Wall (WCW) scale measures overall job satisfaction. (16 items) | | Google Forms | The participants in this study were shown to have a moderate job satisfaction level. Our findings indicate different levels of job satisfaction among female pharmacists of different nationalities, employment status, and promotion expectations. | | This study revealed that the non-Saudi, part-time pharmacists who never expected a promotion were less satisfied than the Saudi, full-time employees who expected a promotion within a year. | |  |
| 28 | suleiman S, Adam S. Job satisfaction among nurses working at primary health center in Ras Al Khaimah, United States Emirates. Int J Nurs Edu. 2020;12(1):65-7. | Cross-sectional, quantitative | To identify the level of job satisfaction among nurses and factors influencing the satisfaction levels among nurses in a primary health center in Ras Al Khaimah. | Unites States Emirates | 18 primary health centers | Not mentioned 30 minutes to collect the data from the sample | nurses | 112 | convenience sample | Unique | | Interview | The intrinsic factors associated with the nurse's satisfaction were regular promotions (0.45,95%), professional autonomy, and advancement opportunities. Intrinsic factors associated with decreased job satisfaction were workload disruption in social life. Extrinsic factors that cause job satisfaction are group cohesion, respect from the management, and regular feedback (0.52, 95%); however, not being paid fairly was the major reason for dissatisfaction. | | The research may help policymakers and practice leaders set retention policies and increase nurse job satisfaction. | |  |
| 29 | Alkassabi OY, Al-Sobayel H, Al-Eisa ES, Buragadda S, Alghadir AH, Iqbal A. Job satisfaction among physiotherapists in Saudi Arabia: does the leadership style matter? BMC Health Serv Res. 2018;18:1-9.  . | Cross-sectional | To examine job satisfaction and influential factors among physiotherapists working in private and government hospitals in Saudi Arabia, focusing on leadership style. | Saudi Arabia | 11 hospitals 6public/government 5 private | Not mentioned | physiotherapist | 69 | Not mentioned | Spector developed the JSS English version to assess the level of job satisfaction among physiotherapists (Spector, 1985) | | email | Job satisfaction was scored as ambivalent, with job satisfaction significantly associated with gender and job subspecialty. | | All the physiotherapists working in government or private hospitals were neither fully satisfied nor fully dissatisfied with their jobs. Female physiotherapists from a musculoskeletal subspecialty of physiotherapy were more satisfied than male physiotherapists from other subspecialties of physiotherapy. Of course, leadership style matters in job satisfaction among physiotherapists in Saudi Arabia. | |  |
| 30 | AlEisa E, Tse C, Alkassabi O, Buragadda S, Melam GR. Predictors of global job satisfaction among Saudi physiotherapists: a descriptive study. Ann Saudi Med. 2015;35(1):46-50. | Cross-sectional observational study | To investigate the level of job satisfaction and identify predictors of job satisfaction among Saudi PTs. | Saudi Arabia | 11 Health facilities and university hospitals | 2013-2014 | physiotherapist | 183 | stratified sample | Unique | | emails | The global job satisfaction seen in our sample was 37%. The highest levels of job satisfaction were seen in professional development and teamwork, and the lowest levels were seen in supervisory/management relationships and working environments. | | Saudi PTs were moderately satisfied with their jobs. Gender, age, relationship with supervisors/managers, work environment, and professional development were the chief determinants of job satisfaction. | |  |
| 31 | Alelyani M, Alqahtani M, Khalid Y, Alamri S, Alghamdi A, Alqahtani E, et al. Job satisfaction among radiologic technologists at hospitals in Saudi Arabia's Southern Region: a cross-sectional study. Biosci Res. 2020;17(3):1659-66. | Cross-sectional | To determine how satisfied the radiology technologists in the hospitals of Aseer province are with their work. | Saudi Arabia | Three main hospitals in Aseer (Public hospitals) | April-Sep 2019 | Radiologic Technologists (radiographers) | 133 | All Radiologic technologists | Minnesota Satisfaction Questionnaire (MSQ) (Weiss et al., 1967) | | emails | Overall, the job satisfaction of radiographers in Aseer hospitals is high (77%). This study established a high degree of feelings of work contentment in our participating radiographers. It also showed that the level of job satisfaction differed between men and women radiographers; men radiographers were much less comfortable (90.3%) with their jobs than women employees (96.7%). Advanced modalities branch employees were the most-satisfied group among all participating individuals in Aseer region hospitals. | | Overall, the job satisfaction of radiographers in Aseer hospitals is high (77%). Men radiological technologists had a lower level of job satisfaction than women staff. | |  |
| 32 | Billah SMB, Saquib N, Zaghloul MS, Rajab AM, Aljundi SMT, Almazrou A, et al. Unique expatriate factors associated with job dissatisfaction among nurses. Intern Nurs Rev. 2021;68(3):358-64. | Cross-sectional | To assess the expatriate nurses' overall job dissatisfaction and identify its significant correlations. | Saudi Arabia | Government sector | Jan-Jun 2017 | expatriate nurses | 977 | All government nurses | They asked the nurses to rate their overall job satisfaction with a single-item question. | | Google forms electronic survey | The mean age of the nurses was 32 years, and 19% reported overall job dissatisfaction. The unique expatriate factors as a group contributed significantly to the model. Job insecurity, patient communication problems, and shorter job duration were significantly associated with higher overall job dissatisfaction. | | Job insecurity, duration, and patient communication significantly correlate with overall job dissatisfaction among expatriate nurses. | |  |
| 33 | Alghamdi MG, Topp R, AlYami MS. The effect of gender on transformational leadership and job satisfaction among Saudi nurses. J Adv Nurs. 2018;74(1):119-27.  . | A secondary analysis of cross-sectional data was used to address the study hypotheses. | To compare nurses' job satisfaction and perceptions of the transformational leadership style of their manager among four different nurse/manager gender dyads in Saudi Arabia. | Saudi Arabia | six general public hospitals in 3 cities | 2011- over 2 months period | Saudi nurses | 308 | convenience sample | Job Satisfaction Survey (JSS) by Spector (1985) and Multifactor Leadership Questionnaire (MLQ) | | Interview | Nurses, regardless of gender, reported higher job satisfaction and perceived transformational leadership style of their manager when their manager was male. | | These findings contrast with what other researchers have reported, which is that nurse job satisfaction and perceived leadership characteristics of their manager are independent of the manager's gender. These perceptions of Saudi nurses may be a result of “sex-role spillover” in a male-dominated, gender-segregated society. | |  |
| 34 | Al-Haroon HI, Al-Qahtani MF. The demographic predictors of job satisfaction among the nurses of a major public hospital in KSA. J Taibah Univ Med Sci. 2020;15(1):32-8. | A quantitative cross-sectional descriptive study | To ascertain the level of job satisfaction and the impact of key demographic factors on job satisfaction among Saudi and non-Saudi nurses working in a public hospital in Dammam. | Saudi Arabia | Public Hospital | April-May 2019 | Nurses | 337 | systematic random sampling | Short version of the previously validated 20-item Minnesota Satisfaction Questionnaire (MSQ) | | Interview | The participants were more satisfied with intrinsic than extrinsic factors in a job. 48% of participants were satisfied, and only 22% were dissatisfied with their jobs. Older nurses tended to report greater job satisfaction and value intrinsic rather than extrinsic factors. | | Most nurses reported moderate levels of overall job satisfaction. Greater job satisfaction was related to demographic factors such as sex (i.e., male), nationality (i.e., Saudi), and age (i.e., older). Job satisfaction was negatively correlated with higher salary. Age and salary predicted the outcome variable. | |  |
| 35 | Ibrahim NK, Alzahrani NA, Batwie AA, Abushal RA, Almogati GG, Sattam MA, et al. Quality of life, job satisfaction and their related factors among nurses working in king Abdulaziz University Hospital, Jeddah, Saudi Arabia. Comtemp Nurs. 2016;51(40):486-98. | Cross-sectional | To assess Quality of Life, job satisfaction and their related factors among nurses working in King Abdulaziz University Hospital, Jeddah, Saudi Arabia. | Saudi Arabia | University Hospital (KAU) | 2014/2015 | Nurses | 268 | stratified random sampling | Job Descriptive Index/Job in General (JDI/JIG) scale to measure short-term satisfaction | | Interview | Most nurses (83.5%) perceived their general QOL as very good and good. Age, marital status, having children, nationality, income, education, residence, working experience, department, and shift time were associated with QOL domains (p < 0.05). Similarly, working experience, income, shifts, and working in inpatient and surgical departments were associated with job satisfaction. Positive correlations were found between job satisfaction and QOL domains, as well as between JDI/JIG subscales. | | Most nurses in the current study perceived their general QOL as very good and good. Improvement of the modifiable factors, such as nurses' income and shift time, is needed for better QOL and job satisfaction. | |  |
| 36 | Aldrees T, Al-Eissa S, Badri M, Aljuhayman A, Zamakhshary M. Physician job satisfaction in Saudi Arabia: insights from a tertiary hospital survey. Ann Saudi Med. 2015;35(3):210-3. | Cross-sectional | To determine the level of job satisfaction of Saudi and non-Saudi physicians of different specialties working in Riyadh, Saudi Arabia, and to explore factors associated with job dissatisfaction. | Saudi Arabia | major tertiary hospital | Oct - Nov 2010 | Physicians | 344 | Not mentioned | Unique | | Interview | 104 (30%) respondents were dissatisfied with their jobs. Intensive care physicians were the most dissatisfied physicians (50%), followed by internists (34%), and the least dissatisfied respondents were emergency medicine specialists (8%). In univariate analyses, dissatisfaction-related factors were income satisfaction, family life affected by the specialization, and positive depression symptoms on the screening test. In the multiple logistic regression model, income satisfaction was the only factor independently associated with dissatisfaction. | | This study explored job satisfaction across different specializations and identified factors that affect satisfaction. Strategic planning should address these factors to improve the healthcare system and patient care. | |  |
| 37 | Allebdi AA, Ibrahim HM. Level and determinants of job satisfaction among Saudi physicians working in primary health-care facilities in Western Region, KSA. J Fam Med Prim Care. 2020;9(9):4656-61.  . | an analytical cross-sectional study | To assess the level of job satisfaction and factors contributing to the dissatisfaction of Saudi physicians in Jeddah's primary health care (PHC) centers. | Saudi Arabia | 45 PHC centers | From 1/3/2016 to 31/9/2016 | Saudi physicians | 119 | all physicians who have contact information in the database of the health directorate | A self-administered informed consent, demographic data, and job satisfaction survey (JSS) questionnaire. JSS (Spector, 1994) | | WhatsApp application and SMS (web-based survey) | 63% of the physicians were satisfied with the nature of work in the primary health care centers, while 25.2% were satisfied with the payment. Many respondents were dissatisfied with the contingent rewards and fringe benefits (83.2% and 76.5 %, respectively). None of the specialists were satisfied with the work compared to 10.6% of the general practitioners. | | Slightly less than half of the physicians are satisfied. Financial incentives (contingent rewards and fringe benefits) negatively impact job satisfaction for primary care physicians. The nature of work has had the most positive impact on job satisfaction. Specialists were found to have less satisfaction. | |  |
| 38 | Jradi H, Alanazi H, Mohammad Y. Psychosocial and occupational factors associated with low back pain among nurses in Saudi Arabia. J Occup Health. 2020;62(1):e12126.  . | Cross-sectional | To assess psychosocial and occupational factors associated with LBP among nurses as a step to take preventive action to alter these modifiable risk factors for better health is a fundamental right for all. | Saudi Arabia | 16 hospitals | 3 months | Nurses | 427 | convenience sample | Unique | | Interview | The prevalence of reported LBP was 80%. Factors associated with LBP in univariate analysis were frequent lifting (OR = 2.32; 95%CI: 1.34-4.01), work-related stress (OR = 5.81; 95%CI: 3,37-9,62), lack of job satisfaction (OR = 2.08; 95%CI: 1.13-3.85), work-related problems (OR = 2.40; 95%CI: 1.44-4.02), and financial problems (OR = 2.08; 95%CI: 1.26-3.38), while factors that remained significantly associated with LBP in the final multivariate analyses were frequent lifting (OR = 2.04; 95%CI:1.09-3.81), work-related stress (OR = 4.22; 95%CI: 2.34-7.48), and lack of job satisfaction (OR = 1.87; 95%CI: 1.24-3.58). | | The prevalence of LBP is high in this healthcare workforce sector. Ergonomic and psychosocial factors may be considered contributing factors for low back pain. Special attention to stress reduction, counseling, and policies to improve job satisfaction are recommended to reduce LBP and improve the health and safety of nurses in Saudi Arabia. | |  |
| 39 | Shubayr N, Faraj H, Hurbush M, Khormi M, Alyami A, Majrashi N, et al. Assessment of job satisfaction, lifestyle behaviors, and occupational burnout symptoms during the COVID-19 pandemic among radiologic technologists in Saudi Arabia. Radiography. 2022;28(4):1087-92.  . | Cross-sectional | To assess the extent of job satisfaction, burnout symptoms, lifestyle behaviors, and associated factors among RTs in Saudi Arabia. | Saudi Arabia | Public and private hospitals | Jan2021-Feb2022 | Radiologic Technologists | 261 | convenience sample | Minnesota Satisfaction Questionnaire (MSQ), a previously validated questionnaire. | | A web-based survey utilizing an electronic Google Form (Google, Mountain View, USA) questionnaire. The questionnaire link was distributed to RTs by their supervisors via email and through social media platforms, WhatsApp (LLC, California, USA) | The overall mean score for job satisfaction was 3.77 (of 5), indicating moderate job satisfaction, with two items showing low satisfaction— compensation (3.33) and advancement (3.28). The overall mean score for lifestyle behaviors was 2.00 (of 3), indicating moderate lifestyle behaviors, with the lowest scores reported in sleep quality (1.92), healthy diet (1.85), and relaxing and unwinding (1.86). The overall mean score for burnout symptoms was 2.30, indicating moderate burnout level, with the highest score reported in experiencing physical symptoms (2.72). There were significant relationships between job satisfaction, lifestyle behaviors, and burnout symptoms. A positive and moderate relationship was found between job satisfaction and healthy lifestyle behaviors, r = 0.53 (p < 0.05). The burnout relationships with job satisfaction, r = −0.615 (p < 0.05), and healthy lifestyle behaviors, r = −0.524 (p < 0.05), were negative and moderate relationships | | The interrelationships between job satisfaction, lifestyle behaviors, and occupational burnout symptoms suggest that improving lifestyle behaviors and managing burnout symptoms could contribute to higher job satisfaction. | |  |
| 40 | Alfuraih AM, Alsaadi MJ, Aldhebaib AM. Job satisfaction of radiographers in Saudi Arabia. Radiolo Techno. 2022;93(3):268-77. | Cross-sectional | To evaluate job satisfaction among radiographers in Saudi Arabia and investigate factors contributing to their scores. | Saudi Arabia | Kingdom of Saudi Arabia | June-July 2020 | Radiographers | 412 | snowball sampling | (JSS) Job Satisfaction Survey by Spector, 1997 | | electronic questionnaire through multiple channels, including specialized social media groups for radiologic technologists in Saudi Arabia, such as Twitter and Telegram. Using a snowball sampling method, participants were encouraged to share the survey with their peers. | The main finding was that radiographers in Saudi Arabia expressed neutral feelings toward their job satisfaction, with most leaning toward higher satisfaction than dissatisfaction. Specifically, they were more satisfied with the nature of work, coworkers, and contingent rewards. However, they were less satisfied with promotions, pay, operating conditions, and benefits. | | Radiographers in Saudi Arabia expressed moderate to high job satisfaction, relatively higher than their international peers and national colleagues in physical therapy and nursing. It also was higher than for MR technologists in the United States and radiographers in the United Kingdom and Lithuania. The highest job satisfaction was associated with the nature of work, contingent rewards, and coworkers. | |  |
| 41 | Almansour H, Gobbi M, Prichard J. Home and expatriate nurses' perceptions of job satisfaction: qualitative findings. Int Nurs Rev. 2022;69(2):125-31.  . | A descriptive qualitative approach- cross-sectional | To increase understanding of the factors influencing job satisfaction of overseas nurses working in Saudi Arabia by comparing the perceptions of home and expatriate nurses. | Saudi Arabia | Three government Hospitals | May 2014 to February 2015 | Nurses | 26 | purposive sampling | Unique | | depth semi-structured interviews (face to face) | Five themes were identified that differentiated the perceptions of expatriates regarding their job satisfaction from those of the home nurses: separation from family, language, and communication, fairness of remuneration, moving into the future, and professionalism. | | The findings identify difficulties for the expatriate group, which negatively impact their job satisfaction and have implications for their intention to stay. Focusing on enhancing job satisfaction experienced by local and expatriate nurses can result in a healthier work environment and greater retention of nurses. | |  |
| 42 | Al Sabei SD, Labrague LJ, Al-Rawajfah O, AbuAlRub R, Burney IA, Jayapal SK. Relationship between interprofessional teamwork and nurses' intent to leave work: The mediating role of job satisfaction and burnout. Nur Forum. 2022;57(4):568-76.  . | Descriptive cross-sectional design | To examine the direct and indirect effects of interprofessional teamwork on nurses' intentions to leave their jobs via the intermediary roles of job satisfaction and burnout. | Oman | 21 Hospitals | June 2019 to February 2020 | Nurses | 2113 | convenience sample | Job satisfaction was measured using a single-item question: "How satisfied are you with your current job?" | | Interview | Interprofessional teamwork was directly associated with nurses' intentions to leave. Both job satisfaction and job burnout indirectly mediated the influence of teamwork on the intention to leave. | | Our finding also highlights the importance of reducing job burnout among nurses, which was significantly and positively associated with turnover intention. | |  |
| 43 | Almansour H, Gobbi M, Prichard J, Ewings S. The association between nationality and nurse job satisfaction in Saudi Arabian hospitals. Int Nurs Rev 2020;67(3):420-6. | A quantitative cross-sectional design | To examine whether there is an association between nationality and nurse job satisfaction. | Saudi Arabia | Three major government hospitals | May 2014 to February 2015 | Nurses | 743 | non-probability sampling | Job satisfaction was measured using the McCloskey/Mueller Satisfaction Scale (Mueller and McCloskey, 1990). The scale consists of 31 items within eight subscales. | | Interview | Compared with Saudi nurses, expatriate nurses had overall lower job satisfaction after controlling for other predictors. While expatriates were less satisfied than Saudi nurses with extrinsic rewards and family–work balance. Saudi nurses were less satisfied with their professional opportunities, praise and recognition, and co-worker relationships. | | For some subscales, Saudi nurses were more satisfied than expatriate nurses, while the opposite was true for other subscales. Nationality should be included in job satisfaction studies in countries with migrant workforces, as nationality-based differences may have been present but masked in earlier international studies by aggregating satisfaction across national groups. | |  |
| 44 | Falatah R, Conway E. Linking relational coordination to nurses’ job satisfaction, affective commitment and turnover intention in Saudi Arabia. J Nurs Manag. 2019;27(4):715-21. | Cross-sectional online survey | To examine the association between relational coordination, job satisfaction, affective commitment and turnover intention. | Saudi Arabia | nurses in Saudi Arabia | N/A | Nurses | 180 | convenience sample | Job satisfaction was measured using the McCloskey/Mueller Satisfaction Scale (MMSS) (Mueller & McCloskey, 1990). | | Online survey (link was posted on six social media pages) | Our findings show that perceptions of relational coordination influence nurse turnover intentions via their effects on job satisfaction and affective commitment. The findings suggest that high levels of relational coordination- achieved through re-configured job designs, performance management, and frequent cross-disciplinary meetings- can give rise to various outcomes of relevance to nurses, allied health professionals, and patients. | | High levels of relational coordination- achieved through re-configured job designs, performance management, and frequent cross-disciplinary meetings- can give rise to various outcomes of relevance to nurses, allied health professionals, and patients. | |  |
| 45 | Falatah R, Almuqati J, Almuqati H, Altunbakti K. Linking nurses' job security to job satisfaction and turnover intention during reform and privatization: a cross-sectional survey. J Nurs Manag. 2021;29(6):1578-86. | A cross-sectional descriptive design | To examine the link between job security, job satisfaction, and turnover intention during the reform and privatization of a health care system. | Saudi Arabia | Seven different tertiary hospitals (MOH) across three cities | N/A | Nurses | 423 | convenience sample | As 'overall, I am satisfied with my job'. Nagy (2002) pointed out that a single-item global satisfaction scale is superior to multiple items. | | Interview | Although job satisfaction partially mediated the association between job security and organizational turnover during health care reform, it completely mediated the association between job security and professional turnover intention. | | Further research is needed to examine this finding. | |  |
| 46 | Parveen M, Maimani K, Kassim NM. A comparative study on job satisfaction between registered nurses and other qualified healthcare professionals. Int J Healthc Manag. 2017;10(4):238-42. | Cross-sectional | To investigate the level of job satisfaction among RNs and OHPs and to explore the relationship of their nationalities and types of healthcare settings with job satisfaction. | Saudi Arabia | Public and private hospitals | N/A | RNs and OHPs | 360 | conveniently distributed | A self-administered questionnaire included two parts: job satisfaction and demographics. The questionnaire was divided into two parts. The first part consisted of 12 items measuring overall job satisfaction. These items were modified and adapted from Traynor and Wade and were equally measured among the three dimensions: personal growth, salary package, and professional support. Unique | | Interview | Results suggested that QHPs were more contented and satisfied with their salary package than RNs. However, both professions were dissatisfied with the personal growth and professional support they received from their employers. | | This study sheds some light on the issues of overall job satisfaction among healthcare professionals in Saudi Arabia. | |  |
| 47 | Alotaibi J, Paliadelis PS, Valenzuela FR. Factors that affect the job satisfaction of Saudi Arabian nurses. J Nurs Manag. 2016;24(3):275-82. | A qualitative- cross-sectional | To determine the levels of job satisfaction and to collect information about the factors that affect the job satisfaction of Saudi nurses. | Saudi Arabia | All Saudi Arabian nurses working at seven hospital sites | Jan-April 2011 | All Saudi nurses | 533 | All Saudis working at seven hospital sites | An existing validated survey (Mueller & McCloskey 1990) (27 items) | | Interview | Four main themes emerged from the data: lack of educational opportunities and support, poor image of the nursing profession, perceptions of favoritism, high workloads, and stressful work environment, and the effect of religion on job satisfaction. | | Saudi nurses would be more satisfied with their jobs if they had greater access to educational opportunities and a reduced workload, and the perceived favoritism in the workplace were addressed. Religion was also found to play a significant role in supporting job satisfaction. | |  |
| 48 | Alharthi H, Youssef A, Radwan S, Al-Muallim S, Zainab A-T. Physician satisfaction with electronic medical records in a major Saudi Government hospital. J Taibah Univ Med Sci. 2014;9(3):213-8. | Cross-sectional analytical observational study | To measure physician satisfaction with a recently introduced electronic medical record (EMR) system and determine which attributes of EMR were related to physician satisfaction. | Saudi Arabia | Governmental hospital in the Eastern province | 30 March-25 May 2010 | Physicians | 115 | N/A | Unique | | Interview | Only 40% were satisfied with the system overall. The best predictors of overall satisfaction were performance in the form of speed, integration with workflow, and patient information, such as accuracy, completeness, and timeliness. | | Physicians were generally not satisfied with the system. Continued evaluation of such systems and user feedback should guide future selection and implementation. | |  |
| 49 | Alqarni T, Alghamdi A, Alzahrani A, Abumelha K, Alqurashi Z, Alsaleh A. Prevalence of stress, burnout, and job satisfaction among mental healthcare professionals in Jeddah, Saudi Arabia. PLoS ONE. 2022;17(4). | A cross-sectional study | To measure the prevalence of stress and burnout and assess job satisfaction among mental healthcare professionals in Jeddah City, Saudi Arabia. | Saudi Arabia | Five psychiatry departments in general hospitals, one hospital for addiction, and one mental health hospital | Jan2017-Oct 2018 | All psychiatrists, psychiatric residents, psychologists, social workers, and psychiatric nurses. | 107 | All psychiatrists, psychiatric residents, psychologists, social workers, and psychiatric nurses. | Job Satisfaction Scale (JSS) (Spector, 1985) | | Interview | Prevalence of stress was 56.1%. High levels of emotional exhaustion and depersonalization were present among 41 (38.3%) and 26 (24.3%) of the respondents, respectively, while high scores of low personal accomplishments were present among 61 (57%) respondents. Regarding job satisfaction, 25 (23.4%) were satisfied, and 74 (69.2%) were indecisive. Male participants’ emotional exhaustion score (27±12) was significantly higher than that of females (22 ±10) (t (105) = 1.99, p-value = 0.049). Also, participants with a monthly income above SR 20,000 had significantly higher total job satisfaction (p-value = 0.041). | | The findings suggest rates of stress and burnout among mental health professionals that warrant attention, with less than one-quarter of the participants being satisfied with their jobs. | |  |
| 50 | Almansour H. Factors influencing job satisfaction among recently qualified resident doctors: a qualitative study. Asia Pac J Health Manag. 2021;16(4).  . | Qualitative approach- cross-sectional | To explore the most crucial factors influencing job satisfaction among recently qualified resident doctors in Saudi Arabia. | Saudi Arabia | 3 Government Hospitals | Dec2019-Jan2020 | resident doctors | 25 | purposive sampling | Unique | | Interview | This study emphasized the importance of improving the satisfaction levels of recently qualified resident doctors concerning various job-related factors. The following seven themes emerged from the data: education and development, recognition and respect, professionalism, workload, healthcare facilities, patient adherence, and salary. | | An increased investment in medical education is recommended to improve educational outcomes. The recently qualified resident doctors' satisfaction with their salaries should be monitored continuously, and further evidence is needed to determine whether modifications are required. Improving the satisfaction levels of the resident doctors in the seven specific areas of their jobs can result in greater retention. | |  |
| 51 | Baghdadi LR, Baghdadi RR, Kamal RS, Obaid EF, Aloqalaa MF, Rambo TW, et al. Physicians' job satisfaction, ethics and burnout in Makkah, Saudi Arabia. J Pak Med Assoc. 2020;70(12):2383-9. | A cross-sectional study | To determine the rates of physicians' job satisfaction, commitment to professional morals, and burnout at Makkah, Saudi Arabia's general hospitals. | Saudi Arabia | 6 Public Hospitals (government) | Apr-May 2016 | Physicians | 136 | random sample | Unique | | Interview | There was no significant difference in job satisfaction among physicians by gender (p-value 0.99). However, a high level of burnout was statistically significant among physicians (51%, p-value <0.001), and the level of burnout was significantly higher among physicians willing to change their specialty compared to those willing to repeat it (50% vs. 24%, p-value 0.02). Physicians dissatisfied with their salary had double the ethics scores compared to satisfied physicians. Female physicians were better at resolving ethical dilemmas. | | This study provided the first evidence of the high frequency of burnout and career choice regret among physicians working in Makkah, Saudi Arabia. Preventing physician burnout not only improves the quality of healthcare but also ensures patient safety. This study mirrors the medical community in S.A., where two-thirds of the physicians are male and work in internal medicine and surgery. Female physicians work primarily in obstetrics and gynecology. | |  |
| 52 | Kader N, Elhusein B, Elhassan NM, Alabdulla M, Hammoudeh S, Hussein N-R. Burnout and job satisfaction among psychiatrists in the Mental Health Service, Hamad Medical Corporation, Qatar. Asian J Psychiatr. 2021;58;102619. | A cross-sectional survey | To determine the prevalence of burnout and job satisfaction among psychiatrists in the Mental Health Service, Hamad Medical Corporation, Qatar, and examine correlations among socio-demographic variables, burnout, and job satisfaction. | Qatar | Hamad Medical Corporation (HMC) | N/A | Psychiatrists | 73 | All psychiatrists | (JDI) was used to measure job satisfaction and comprises five facets: satisfaction with co-workers, the work itself, pay, promotion opportunities, and supervision. It also includes jobs in general (JIG), which is considered a global measure of job satisfaction. | | Interview | One-third of psychiatrists reported high levels of emotional exhaustion, with a similar proportion describing low levels of personal accomplishment. Less than 20 % demonstrated high levels of depersonalization. Trainees were burned out more than senior psychiatrists. Opportunities for promotion were the only factor with which most psychiatrists were not satisfied. | | The prevalence of high burnout in psychiatrists remains lower in Qatar than in other countries. Lower levels of satisfaction with co-workers, work, supervision, opportunities for promotion, and the job in general increased emotional exhaustion and depersonalization. Interestingly, satisfaction with salary did not have a significant effect on the burnout. | |  |
| 53 | Banaser M, Ghulman‬ F, Almakhalas H, Alghamdi M. Nurses’ job satisfaction during the mass gathering of the Hajj 2018 in Saudi Arabia. Int Nurs Rev. 2020;67(3):372-9. | A cross-sectional survey | To explore nurses' job satisfaction when providing health care during the pilgrimage of Hajj in Saudi Arabia | Saudi Arabia | 12 healthcare settings along the Hajj pilgrimage route | 18 to 28 Aug 2018 | Nurses | 198 | convenience sample | Unique | | email | Nurses reported a high level of job satisfaction. All six domains were strong predictors of job satisfaction. Nurses reported the lowest level of job satisfaction when assessed against the multiple policies and procedures perceived as complicating nursing work,’ ‘incompetence of other people they work with,’ and ‘too much burden at work.’ | | Job satisfaction levels for nurses during Hajj 2018 appeared higher than during previous Hajj pilgrimages, probably influenced by the additional healthcare measures put in place during Hajj 2018, such as improved technology. | |  |
| 54 | Mirza AA, Badrek-Amoudi AH, Farooq MU, Senan HA, Aun RH, Mirza AA, et al. Job satisfaction amongst surgical healthcare professionals during Hajj and Non-Hajj periods: An analytical multi-center cross-sectional study in the holy city of Makkah, Saudi Arabia. J Pak Med Assoc. 2020;70(8):1371-5. | A multicenter cross-sectional study | To assess the job satisfaction of surgical healthcare professionals working during Hajj and determine the potential predictors of overall job satisfaction in Hajj and non-Hajj periods. | Saudi Arabia | 3 Public Hospitals (government) | 27Aug-5Sept2018 | Surgical Healthcare professional | 146 | All surgeons who were employed during Hajj | a short version of the Warr-Cook-Wall (WCW) job satisfaction scale. | | Interview | Overall job satisfaction scores in the Hajj period were 5.53 ±1.19 compared to 5.40±1.33 in the non-Hajj period (p>0.05). During the Hajj period, the participants were significantly more satisfied in terms of the "amount of variety in work" (p<0.05), while they were significantly dissatisfied concerning "physical work in g condition," "hours of work" and "attention paid to suggestions" (p<0.05). | | The variety of cases encountered by surgeons during Hajj contributed significantly to job satisfaction. However, lack of attention to physicians' suggestions, extended work hours, and physical working conditions during Hajj were potential factors causing less Job satisfaction during Hajj duty. | |  |
| 55 | AlJumail E, Rabbani U. Job satisfaction among primary health care workers in Buraidah, Qassim, Saudi Arabia. World Family Medicine. 2021;19(12):27-33. | A cross-sectional study | To assess the job satisfaction among primary health care (PHC) workers in Buraidah, Saudi Arabia. | Saudia Arabia | 20 PHC centers | Nov 2019 to the beginning of 2021 | All workers: physician, dentist, nurse, pharmacist, and laboratory and radiology technicians | 230 | random sample | Job Satisfactions Survey (JSS) (Spector, 1985). | | Interview | Nineteen (8.3%) of them expressed that they were dissatisfied, almost half of them (55.7%) were ambivalent, and the rest (36.1%) were satisfied with their job. Among the nine facets of the JSS, Nature of work, Co-workers, and Supervision had mean scores in the satisfaction category, while five facets were in the range of ambivalent: Pay, Promotion, Benefits. Contingent rewards and Communication. Only one facet fell under the dissatisfaction range, which was Operating Conditions. None of the socio-demographic variables had a significant association with job satisfaction. | | Almost two-thirds of the workers were not satisfied with their jobs, which may have affected their performance and quality of care. Operating conditions were found to be the factor with the lowest satisfaction. On the other hand, co-workers and the nature of work positively impact their satisfaction. | |  |
| 56 | Alrawahi S, Sellgren SF, Alwahaibi N, Altouby S, Brommels M. Factors affecting job satisfaction among medical laboratory technologists in university hospital, Oman: an exploratory study. Intern J Health Plan Manag. 2019;3g4(1):e763-e75. | A case study, Focus Group Discussions. (Qualitative nature)- cross-sectional | This study explores the factors that impact job satisfaction among medical laboratory technologists at University Hospital, Oman. | Oman | University Hospital | Dec2015-Jan2016 | senior and junior medical laboratory technologists | 77 | All laboratory technologists | Unique | | Interview (FGD) | Factors that caused major dissatisfaction at work were identified as follows: workload, promotion, health and safety in the laboratory, relationship with the leaders, professional status (recognition and appreciation), and hospital policies such as appraisal. Stress was the outcome of these factors' presence/absence/quality. Other important factors that emerged were autonomy and professional development. The satisfaction factors were identified as salary, co-worker relationships, and job security for non-Omanis. | | These findings call for evaluating the promotion system, improving health and safety within the laboratories, evaluating the technologists’ careers within their laboratories, and improving professional training programs for career enhancement. | |  |
| 57 | Amer YS, Al Nemri A, Osman ME, Saeed E, Assiri AM, Mohamed S. Perception, attitude, and satisfaction of paediatric physicians and nurses towards clinical practice guidelines at a university teaching hospital. J Eval in Clin Pract. 2019;25(4):543-9.  . | A cross-sectional survey | To explore the perception, attitude, and satisfaction of pediatric physicians, residents, interns, and nurses towards clinical practice guidelines (CPGs) in general plus towards the implemented diabetic ketoacidosis CPG (DKA-CPG) in particular at the Pediatrics Department at King Khalid University Hospital (KKUH). | Saudi Arabia | University teaching hospital | N/A | Doctors and nurses | 248 | convenience sample | Single question | | on-site | The response rate was 95.4%. The respondents had a positive perception and attitude towards general CPGs and specifically for the DKA-CPG; 98.7% thought CPGs were useful sources of advice, improved safety, decreased risk, and reduced variation in practice. 99.2% thought CPGs were good clinical tools, 98.3% were satisfied with them, had confidence in well-developed CPGs, and would recommend them to their colleagues for use. 94.6% agreed they were cost-effective. The preferred format for CPGs was paper (46.6%) and electronic (42.9%). The DKA-CPG helped manage patients; respondents were satisfied and confident (100%). The rationale and objectives of the DKA-CPG were clear for 99.25%; 98.5% thought the layout was clear, well-organized, and user-friendly (96.2%). Compared with nurses, physicians had a higher perception towards CPGs in general (P <.05) and the DKA-CPG (P <.05). | | Pediatric doctors and nurses have a great perception of satisfaction and a positive attitude towards CPGs in general, towards pediatric diabetic ketoacidosis CPG in particular, which positively impacted the acceptability and implementation of CPGs. These findings could help sustain a safe and high-quality healthcare environment by implementing evidence-based CPGs. | |  |
| 58 | Halawani LA, Halawani MA, Beyari GM. Job satisfaction among Saudi healthcare workers and its impact on the quality of health services. J Fam Med Prim Care. 2021;10(5):1873-81.  . | A cross-sectional questionnaire-based study | To assess the impact of job satisfaction on the quality of health services | Saudi Arabia | Public and private hospitals | N/A | Saudi citizens' healthcare workers | 226 | random sample | Unique | | Online well-constructed questionnaire was sent electronically | Of 26 participants, 73.9% were females, 26.1% were males, and the majority (n = 108, 47.8%) were aged between 31 to 40 years old. Half of the participants (50%) were married, 37.2% were physicians, 70.8% worked at a public organization, and 61.9% had more than 3 years of work experience. Stress management and patient satisfaction significantly impacted the participant's job satisfaction (P = 0.001 and P = 0.021, respectively). Poor management was the highest factor that affected the quality of the care provided in the hospitals (40%), but there was no significant difference (P-value = 0.210). The participants showed neutral satisfaction with their work (38.1%), and most had low satisfaction (n = 119, 52.7%). | | Job satisfaction and the quality of care provided are strongly associated. The overall level of job satisfaction among health care providers in Saudi Arabia was low. | |  |
| 59 | Hamasha AA, Alturki A, Alghofaili N, Alhomaied A, Alsanee F, Aljaghwani F, et al. Predictors and level of job satisfaction among the dental workforce in national guard health affairs. J Int Soc Prev Community Dent. 2019;9(1):89-93.  . | A cross-sectional study | To assess the level of job satisfaction among the dental workforce in the National Guard Health Affairs (NGHA) in Saudi Arabia and to explore any predictors that impact the level of satisfaction. | Saudi Arabia | National Guard Health Affairs and clinics in Riyadh | These centers were visited several times over 1 month starting from February 2018 (1 month) | Dental health-care workers: dentists, dental assistants, dental hygienists, and dental lab technicians | 198 | A cluster random sampling | Unique | | Interview | The response rate was 55.5%. The mean satisfaction score was 65.7 out of 112 (2.9 out of 5). Of the four domains about job satisfaction, the highest mean score was obtained for quality of service (4.2 out of 5), followed by prestige and self-perception (2.8 out of 5), professional and personal life (2.8 out of 5), and income and job security (2.6 out of 5). Non-Saudis were found to be significantly more satisfied in their professional and personal life; however, Saudi dentists were more satisfied with income, job security, and quality of services. | | In general, the dental workforce practicing in NGHA experience low job satisfaction. Professional and personal life, income and job security, and quality of services are all critical factors affecting job satisfaction. | |  |
| 60 | Alghamdi NG, Khan K. Job satisfaction and organizational commitment of paramedics - a case study of Saudi Arabia. J Comput Theor Nanosc. 2018;15(4):1283-90.  . | A cross-sectional study | To look further into the association among job satisfaction (JS) dimensions and organizational commitment (OC) vis-a-vis paramedical staff in Saudi Arabia. | Saudi Arabia | Four private hospitals | N/A | Paramedics | 110 | convenience sampling | The Job Descriptive Index (JDI) was developed by (Smith et al., 1969) [12] to measure job satisfaction. | | Interview | The results showed that Job Satisfaction explained 20 percent of the variation in Organizational Commitment. The five dimensions explain 24 percent of the variation in Organizational Commitment. The ones with the highest impact are co-workers. The findings of the current research are significant for several reasons. First, it has a fundamental practical implication for hospital managers to look where it matters most. Second, by looking into the dimensions of JS and their relationship with OC, one can quickly determine what needs to be focused on to enhance JS and OC simultaneously. | | To not further aggravate the current shortage of paramedical personnel, the results show that hospital administrators should adopt policies that can enhance job satisfaction and, resultantly, organizational commitment of the paramedics, which is the second most prominent workforce element in a healthcare facility. | |  |
| 61 | Alrawahi S, Sellgren SF, Altouby S, Alwahaibi N, Brommels M. The application of Herzberg's two-factor theory of motivation to job satisfaction in clinical laboratories in Omani hospitals. Heliyon. 2020;6(9):e04829 | A mixed-method approach- cross-sectional | To explore the motivational elements associated with job satisfaction among medical laboratory professionals (MLPs) in Oman. | Oman | Three main MOH hospitals | Feb - Jun 2017 | Medical laboratory professionals (MLPs) | 101 | All staff | Unique | | Interview (FGD) | The following job dissatisfaction factors (hygiene) were identified: health and safety, heavy workload, salary, promotion, recognition, and organizational policies. The satisfaction (motivators) were relationships with co-workers, relationships with leaders, and professional development. | | The job dissatisfaction reported resulted from the absence of hygiene factors and some motivators per Hertzberg's theory. As defined by Hertzberg, hospital managers need to address these factors to improve motivation and job satisfaction. | |  |
| 62 | Al Jazairy YH, Halawany HS, Al Hussainan N, Al Maflehi N, Abraham NB, Jacob V. Factors affecting job satisfaction and their correlation with educational standards among dental assistants. Ind Health. 2014;52(4):324-33. | A cross-sectional survey | To assess the level and distribution of job satisfaction among dental assistants working in various private and public hospitals, clinics, polyclinics, and dental schools in the Kingdom of Saudi Arabia | Saudi Arabia | Eight public and six private hospitals | 5 months from Feb 2013 | Dental assistants | 498 | All dental assistants working in public and private institutions | Unique | | Interview | The overall response rate was 72.1%. Factor analysis suggested that five underlying factors were related to job satisfaction. The mean score for overall job satisfaction was 3.86 (satisfied) out of 5. Among the work environment factors, the highest mean score, 4.26 (satisfied), was obtained for quality of service, and the lowest mean score, 2.78 (neutral), was obtained for the perception of income. The income and general prospects of the profession were significantly associated with overall job satisfaction. | | This study suggests that for dental assistants, professional and personal life, quality of service, perception of income and prestige, and self-respect are essential for job satisfaction. Despite differences in professional formation standards, the study participants generally were considerably satisfied with their jobs. | |  |
| 63 | Alenazy FS, Dettrick Z, Keogh S. The relationship between practice environment, job satisfaction and intention to leave in critical care nurses. Nurs Critic Care. 2023;28(2):167-76. | Cross-sectional correlational (observational) design | To examine the relationship between perception of nursing practice environment (NPE), job satisfaction, and intention to leave (ITL) among critical care nurses working in the state of Ha'il in KSA. | Saudi Arabia | King Khalid Hospital (KKH) | 27July -27Aug 2020 | ICU nurses | 152 | All nurses working within the CCU | Nursing Workplace Satisfaction Questionnaire (NWSQ) | | electronic online survey | The NPE was largely favorable (M = 2.89, SD = 0.44); however, nurse participation in hospital affairs (M = 2.83, SD = 0.47) and staffing and resource adequacy (M = 2.88, SD = 0.47) scored lowest. NPE was found to be significantly correlated with job satisfaction (Rs =.287, P <.01). A significant negative relationship was found between NPE and ITL (Rs = −0.277**, P <.01). However, job satisfaction was associated with ITL (Rs = −.007, P =.930). | | Maintaining a healthy work environment and job satisfaction levels in critical care units is key to improving nursing staff recruitment and retention. | |  |
| 64 | Al-Muallem N, Al-Surimi KM. Job satisfaction, work commitment and intention to leave among pharmacists: a cross-sectional study. BMJ Open. 2019;9(9):e024448. | A cross-sectional study | To assess job satisfaction, work commitment, and intention to leave among pharmacists working in different healthcare settings in Saudi Arabia. | Saudi Arabia | Public and private hospitals, community chain pharmacies, community independent pharmacies, primary care center pharmacies, industrial pharmacies, and academic pharmacies. | N/A | Pharmacists | 325 | Pharmacists licensed by the Saudi Commission for Health Specialties and working in Riyadh. | Unique | | Online survey via email | Although most of the pharmacists were satisfied (satisfied and slightly satisfied) with their current job (39.1% and 24.6%, respectively), about two-thirds (61.9%) had the intention to leave. Multiple logistic regression analysis showed that the most important predictors of pharmacists' intentions to leave were related to job satisfaction and work commitment (OR=0.923; 95% CI 0.899 to 0.947; p<0.001 and OR=1.044; 95% CI 1.014 to 1.08; p=0.004, respectively), whereas respondents' demographic characteristics had no effect. | | Although the pharmacists surveyed were satisfied and committed to their current job, they had the intention of leaving. Further research is recommended to clarify why pharmacists in Saudi Arabia have the intention to leave their pharmacy practice job. | |  |
| 65 | Baker OG, Alshehri BD. The relationship between job stress and job satisfaction among Saudi nurses: a cross-sectional study. Nurse Media J Nurs. 2021;10(3):292-305.  . | A cross-sectional design | To examine the relationship between work-related stress and job satisfaction among Saudi nurses at a public hospital. | Saudi Arabia | specified public hospital | July-Dec 2017 | Saudi nurses | 297 | convenient sampling | Job Satisfaction Scale (JSS) original version. (Spector, 1985) | | Interview | The results indicated that the nurses at the hospital where the study occurred experienced low stress levels with a mean value of 2.1995. Besides, the nature of work indicated maximum job satisfaction with a mean value of 15.666, whereas minimum job satisfaction levels (11.569) were related to benefits provided to nurses. A positive correlation was found between stress and satisfaction, with a p-value of 0.041. | | The stress factors were highly correlated with job satisfaction. Identifying stress factors is important as they may negatively impact patients' care and well-being. | |  |
| 66 | Hussain MK, Khayat RAM. The impact of transformational leadership on job satisfaction and organisational commitment among hospital staff: a systematic review. J Health Manag. 2021;23(4):614-30. | A systematic review involves four electronic databases | To examine the impact of transformational leadership on job satisfaction and organizational commitment among hospital staff. | Saudi Arabia | four electronic databases | 1985- Aug 2016 | Hospital staff | 26 studies included in this review | Systematic review | Unique | | Systematic Review | Three hundred sixty-seven titles and abstracts were screened, yielding 26 studies included in this review. The collected variables are analyzed to determine the effect of transformational leadership style on job satisfaction and organizational commitment. A total of 19 studies examining the relationship between transformational leadership and job satisfaction (n = 15) were found to have a positive relationship, while 13 studies examining the relationship between transformational leadership and organizational commitment (n = 11) were found to have a positive relationship. | | The results of the review provide evidence that transformational leadership has a significant effect on hospital staff and the hospital environment. The transformational leadership style should be universally used in hospitals to increase medical staff job satisfaction and organizational commitment. This may lead to increased productivity, quality of healthcare services, and patients’ safety. | |  |
| 67 | Almarashi AM, Al Wadei S, Alajaj AS, Alshoqiqe AZM, Altherwi MN, Almubarak SH, et al. Job satisfaction and organizational commitment of doctors: a case study of Saudi Arabia. Jp J Biostat. 2022;19(1):15-35. | A cross-sectional study, quantitative and correlation | The present study focuses on job satisfaction (JS) and organizational commitment (OC) vis-a-vis doctors in Saudi Arabia. | Saudi Arabia | Public hospitals | N/A | Doctors | 170 | Convenience sampling | Job Descriptive Index (JDI) | | Interview | The results showed that promotion has the highest impact on JS and OC. Also, JS is a highly significant predictor of OC. | | The present study affords new support to previous research about the importance of JS and OC for enhancing organizational effectiveness and performance. | |  |
| 68 | Parveen M, Maimani K, Kassim NM. Quality of work life: the determinants of job satisfaction and job retention among RNs and OHPs. Int J Quality Res. 2017;11(1):173-94. | Cross-sectional | To examine the effects of demographic characteristics on the QWL dimensions and satisfaction and the relationships between the QWL dimensions and satisfaction and between satisfaction and retention among RNs and OHPs. | Saudi Arabia | Public and private hospitals | N/A | Registered nurses and other health care professionals | 360 | Not mentioned | Satisfaction (12 items) from Traynor and Wade (1993). (MJS) | | Interview | The results show a significant difference between demographic and QWL dimensions and satisfaction. Satisfaction with personal growth and salary package significantly impacted overall retention. The paper provides a greater understanding of QWL, satisfaction, and retention, as well as their relationships with each other among the RNs and OHPs in public and private healthcare organizations in Saudi Arabia. | | The current study is cast as a foundation stone that strongly supports our understanding of the quality of work life, job satisfaction, and retention of RNs and OHPs in public and private healthcare organizations. | |  |
| 69 | Alqahtani AMA, Nahar S, Almosa K, Almusa AA, Al-Shahrani BF, Asiri AA, et al. Leadership styles and job satisfaction among healthcare providers in primary health care centers. World Family Medicine. 2021;19(3):102-12. | A cross-sectional design | To explore the relationship between managers' leadership styles and job satisfaction among healthcare workers in primary healthcare centers (PHCCs) in Aseer Region, Saudi Arabia. | Saudi Arabia | 25 PHCCs in Abha and Khamis Mushait cities | N/A | 25 PHCC managers and 300 PHC providers | 325 | Cluster sample technique and systematic random sampling technique | "Job Satisfaction Survey Questionnaire" JSS (Spector, 1985) | | Interview | Only 52% of PHCC managers attended training on leadership, 68% highly followed the transformational leadership facet "idealized influence," and 64% followed "management-by-exception," while only 28% highly followed the "Laissez-faire" leadership style. About one-third of healthcare providers were satisfied, 9.3% were dissatisfied, and 51.3% were ambivalent. Laissez-faire leadership style correlates significantly with most healthcare providers' job satisfaction, followed by transactional and transformational leadership. | | Job satisfaction is significantly lower among pharmacists, those with insufficient income, or those with less experience in PHC. Laissez-faire leadership significantly correlates with most job satisfaction items, followed by transactional and transformational leadership. It is necessary to improve PHC providers' job satisfaction by applying transformational and transactional leadership styles better. | |  |
| 70 | Al-Ghareeb HY, Al-Wateyan RA. Job satisfaction in PHC Kuwait. World Family Medicine. 2019;17(5):4-15. | Comparative cross-sectional study | To assess employee satisfaction regarding their job satisfaction determinants and quality and safety, determine the view and preferences of the healthcare personnel regarding the current health system, and the recent introduction of an accreditation program to a better understanding of its impact on quality of care as perceived by BHC staff members and directors to identify possible strategies to improve implementation of accreditation in PHC and identify barriers and problems the employee faces in the PHC centers in Kuwait. | Kuwait | 60 PHC centers in five health regions | one year (September 2016 to September 2017) | (physicians, nurses, pharmacists, and assistant pharmacists, lab technicians, x-ray technicians, administrative) | 3969 | All employees who were available at the time | Unique | | Interview | The response rate for all staff was 55%; the highest respondent rate was for nurses (74%), and the lowest respondent rate was for administrative staff (34%) and assistant nurses (34%). The results contribute to understanding factors influencing satisfaction levels between primary healthcare staff and interventions that need to be implemented to improve job satisfaction among healthcare professionals. Implementing accreditation is an essential first step toward improving the quality and safety of PHC centers. | | The resultsf contribute to understanding factors influencing satisfaction levels between primary healthcare staff and interventions that need to be implemented to improve job satisfaction among healthcare professionals. Implementing accreditation is an important first step toward improving the quality and safety of PHC centers. The findings of this study showed that job satisfaction is poor in some dimensions, which need improvements in the future, and good in others which needs continuation and enhancements. | |  |
| 71 | Slimane NSB. Motivation and job satisfaction of pharmacists in four hospitals in Saudi Arabia. J Health Manag. 2017;19(1):39-72. | A constructionist research design through a mixed theory of quantitative and qualitative questionnaires- mixed cross-sectional | To determine what motivates pharmacists, measure job satisfaction, determine the influencing factors, and compare it with pharmacy managers’ and supervisors’ opinions. | Saudi Arabia | Four different hospitals: two tertiary  government hospitals and two private hospitals | four weeks | Pharmacists | 71 pharmacists, 19 managers | Convenience sampling | Minnesota Satisfaction Questionnaire style, Warr–Cook–Wall scale (Warr, Cook, & Wall, 1979) (WCW) | | Interview | Results showed salary, promotion, recognition, and job satisfaction as the most potent motivator factors. A statistically significant relation between job satisfaction and task significance, autonomy, and managers’ feedback. Managers consider salary, promotion, financial rewards, and vacation the most important motivators. Results confirmed that managers’ opinions do not reflect their department’s true motivators and job satisfaction. | | Managers need to eliminate dissatisfaction, which is determined by the hygiene factors of co-workers’ relationships, salaries, and job security. Enhance the satisfaction effect related to motivator factors of promotion, recognition, and work itself. It also confirmed previous findings of job satisfaction being a dynamic multi-dimensional phenomenon. | |  |
| 72 | Brant JM, Fink RM, Thompson C, Li YH, Rassouli M, Majima T, et al. Global survey of the roles, satisfaction, and barriers of home health care nurses on the provision of palliative care. J Pallat Med. 2019;22(8):945-60.  . | Cross-sectional | To globally examine home health care nurses' practice, satisfaction, and barriers regarding existing palliative home care provision. | 29 countries, including UAE and Saudi Arabia | Five hundred thirty-two home health care nurses | Nov 2017 - Apr2018 | Home health care nurses | 532 | convenience sample | Unique | | Survey Monkey e-mail link or paper | Nurses from developing countries performed more duties than those from high-income countries, suggesting a lack of resources in developing countries. Significant barriers to home care include personnel shortages, lack of funding and policies, poor access to end-of-life or hospice services, and decreased community awareness of services provided. Respondents identified lack of time, funding, and coverage as primary educational barriers. In-person local meetings and online courses were suggested as strategies to promote learning. | | It is imperative that home health care nurses have adequate resources to build PC capacity globally, which is so desperately needed. Nurses must be current on current evidence and practice within an evidence-based PC framework. Health care policy to increase necessary resources and the development of a multifaceted intervention to facilitate education about PC is indicated to build global capacity. | |  |
| 73 | Saleh U, O'Connor T, Al-Subhi H, Alkattan R, Al-Harbi S, Patton D. The impact of nurse managers' leadership styles on ward staff. Br J Nurs. 2018;27(4):197-203.  . | Qualitative methodology- cross-sectional | To explore the nature of leadership styles used by the nursing management team, as perceived by nurses working at the bedside | Saudi Arabia | Medical City | N/A | Nurses | 35 | A purposive, non-probability sample | Unique | | Interview | The findings showed that participants described four types of leadership styles: relational leadership, preferential leadership, communication chain leadership, and ineffectual leadership. | | The leadership style employed by nurse managers has a major impact on nurses’ satisfaction, turnover, and the quality of patient care they deliver. | |  |

**Appendix 3 (page 4 line 261 for your help)**

| **COHORT STUDY** | | |  |  |  |  |  |  |  |  |  |  |  |  |  |  |  |  |  |  |  |  |
| --- | --- | --- | --- | --- | --- | --- | --- | --- | --- | --- | --- | --- | --- | --- | --- | --- | --- | --- | --- | --- | --- | --- |
|  |  |  |  |  |  |  |  |  |  |  |  |  |  |  |  |  |  |  |  |  |  |  |
|  |  |  | (A) Are the results of the review valid? | | | | | | | | | (B) What are the results? | | | | (C) Will the results help locally? | | | |  |  |  |
|  |  |  |  |  |  |  |  |  |  |  |  |  |  |  |  |  |  |  |  |  |  |  |
|  |  |  | Question 1 | | Question 2 | |  | Question 3 | | Question 4 | | Question 5 | | Question 6 | | Question 7 | | Question 8 | | Question 9 | |  |
|  | NAME ARTICLE | LINK ARTICLE | Did the study address a clearly focused issue? | | Was the cohort recruited in an acceptable way? | | IS WORTH TO CONTINUING? | (a) Have the authors identified all important  confounding factors? | | (b) Have they taken account of the  confounding factors in the design and/or analysis? | | Do the results valid and precise | | How precise are the results? | | Can the results be applied to the local population? | | Do the results of this study fit with other  available evidence? | | What are the implications of this study for practice? | |  |
|  |  |  | Score | Description | Score | Description |  | Score | Description | Score | Description | Score | Description | Score | Description | Score | Description | Score | Description | Score | Description | TOTAL SCORE |
| 1 | International Nursing: Job Satisfaction among Critical Care Nurses in a Governmental Hospital in Saudi Arabia | [file:///C:/Users/mtok1/OneDrive/%D8%B3%D8%B7%D8%AD%20%D8%A7%D9%84%D9%85%D9%83%D8%AA%D8%A8/Included%20Articles/International%20Nursing%20Job%20Satisfaction%20among%20Critical%20Care%20Nurses%20in%20a%20Governmental%20Hospital%20in%20Saudi%20Arabia.pdf](file:///C:\Users\mtok1\OneDrive\Ø³Ø·Ø­%20Ø§ÙÙÙØªØ¨\Included%20Articles\International%20Nursing%20Job%20Satisfaction%20among%20Critical%20Care%20Nurses%20in%20a%20Governmental%20Hospital%20in%20Saudi%20Arabia.pdf) | 2 | To measure factors that affect job satisfaction among critical care nurses at King Khalid Hospital in Saudi Arabia. | 1 | convenient sampling | YES | 2 | Job satisfaction survey, demographic | 1 | Bivariate analysis | 2 | Found positive satisfaction with relationships with coworkers, nature of their jobs, communication, pay, and supervision. | 2 | Used 95% confidence interval | 0 | It focused on critical care nurses only, and it can be considered a base for future studies of nurse satisfaction in other health care settings. | 2 | Consistency | 2 | Important for decision makers | 14 |
| 2 | Job satisfaction among occupational therapists working in Riyadh, Saudi Arabia | [file:///C:/Users/mtok1/OneDrive/%D8%B3%D8%B7%D8%AD%20%D8%A7%D9%84%D9%85%D9%83%D8%AA%D8%A8/Included%20Articles/Job%20satisfaction%20among%20occupational%20therapists%20working%20in%20Riyadh,%20Saudi%20Arabia.pdf](file:///C:\Users\mtok1\OneDrive\Ø³Ø·Ø­%20Ø§ÙÙÙØªØ¨\Included%20Articles\Job%20satisfaction%20among%20occupational%20therapists%20working%20in%20Riyadh,%20Saudi%20Arabia.pdf) | 2 | To identify the level of job satisfaction of occupational therapists. | 0 | nonprobability purposive sampling | YES | 2 | A validated job satisfaction questionnaire, Major of job satisfaction questionnaire, and demographic information | 0 | SPSS, frequencies and percentages, and Chi-square test | 2 | Found positive satisfaction with coworkers, care provided to client, and autonomy. Negative satisfaction with salary compared to the work efforts, lack of reimbursement for continuing education, and lack of opportunities for career advancement. | 2 | Used 95% confidence interval | 0 | limited variety of settings, design of the study, and the sample size was small in the study. | 2 | Consistency | 2 | Important for occupational therapy managers and managers of human resources | 12 |
| 3 | Physicians' Job Satisfaction and its Correlates in a Tertiary Medical Care Center, Riyadh, Saudi Arabia | [file:///C:/Users/mtok1/OneDrive/%D8%B3%D8%B7%D8%AD%20%D8%A7%D9%84%D9%85%D9%83%D8%AA%D8%A8/Included%20Articles/Physicians%E2%80%99%20Job%20Satisfaction%20and%20its%20Correlates%20in%20a%20Tertiary%20Medical.pdf](file:///C:\Users\mtok1\OneDrive\Ø³Ø·Ø­%20Ø§ÙÙÙØªØ¨\Included%20Articles\Physiciansâ%20Job%20Satisfaction%20and%20its%20Correlates%20in%20a%20Tertiary%20Medical.pdf) | 2 | To measure the degree of job satisfaction among physicians working in a Tertiary Care Hospital and to identify background and work environment characteristics that affect overall and differential job satisfaction. | 2 | A stratified random sample | YES | 2 | A self-administered questionnaire including demographic variables | 0 | Descriptive statistics including mean standard deviation, Inferential statistics that is t-test, one-way ANOVA, and Chi-square test, and Pearson correlation (r) | 2 | Found positive satisfaction with stipend, duration of vacation leave, sick leave policy, health coverage for the employee and family, overall benefits package, involvement in academic work, and involvement in research work. Negative satisfaction with the number of children the physician has | 2 | Used 95% confidence interval | 0 | N/A | 2 | Consistency | 0 | N/A | 12 |
| 4 | Job satisfaction of nurses in a Saudi Arabian university teaching hospital: a cross-sectional study | [file:///C:/Users/mtok1/OneDrive/%D8%B3%D8%B7%D8%AD%20%D8%A7%D9%84%D9%85%D9%83%D8%AA%D8%A8/Included%20Articles/Al-dossary-2012-Job-satisfaction-of-nurses-in-a-sau.pdf](file:///C:\Users\mtok1\OneDrive\Ø³Ø·Ø­%20Ø§ÙÙÙØªØ¨\Included%20Articles\Al-dossary-2012-Job-satisfaction-of-nurses-in-a-sau.pdf) | 2 | To measure nurses' job satisfaction in Saudi Arabia in a university teaching hospital and determine the influencing factors | 0 | A systematic sampling | YES | 2 | a self-administered questionnaire, job satisfaction survey, and demographic information | 0 | Descriptive statistics, inferential statistics, t-test and one-way analysis of variance (ANOVA) | 2 | Found positive satisfaction with supervision, co-workers, and nature of work. Negative satisfaction with pay, fringe benefits, contingent rewards, and operating conditions. | 2 | Used 95% confidence interval | 0 | single university hospital, modest sample size, disparity in the number of Saudi and non-Saudi nurse, insufficient amount of information and resources regarding job satisfaction among nurses. | 2 | Consistency | 2 | Important for nursing managers and policy makers | 12 |
| 5 | The relationships between nurses' work environments and emotional exhaustion, job satisfaction, and intent to leave among nurses in Saudi Arabia | [file:///C:/Users/mtok1/OneDrive/%D8%B3%D8%B7%D8%AD%20%D8%A7%D9%84%D9%85%D9%83%D8%AA%D8%A8/Included%20Articles/A.%20A.%20Alharbi%20-%20The%20relationships%20between%20nurses%E2%80%99%20work%20environments%20and%20emotional%20exhaustion,%20job%20satisfaction,%20and%20intent%20to%20leave%20among%20nurses%20in%20Saudi%20Arabia.pdf](file:///C:\Users\mtok1\OneDrive\Ø³Ø·Ø­%20Ø§ÙÙÙØªØ¨\Included%20Articles\A.%20A.%20Alharbi%20-%20The%20relationships%20between%20nursesâ%20work%20environments%20and%20emotional%20exhaustion,%20job%20satisfaction,%20and%20intent%20to%20leave%20among%20nurses%20in%20Saudi%20Arabia.pdf) | 2 | To examine relationships between components of nurses' work environments and emotional exhaustion, job satisfaction and intent to leave among nurses in Saudi Arabia. | 1 | Convenience sample | YES | 2 | RN4cast survey, BC Nurses' Workload Impact Study, PES-NWI, Emotional Exhaustion subscale of the Maslach Burnout Inventory Human Service Scale, job satisfaction, Nurses' intent to leave, and Nurses' Demographic Characteristics | 2 | Hierarchical linear regression, bivariate test, and hierarchical logistic regression. Also, multiple regression | 2 | Found positive satisfaction with nurse participation in hospital affairs, nurse manager ability and leadership support for nurses, and staffing and resource adequacy, years of nursing experience, and receiving basic nursing education in India | 2 | Used 95% confidence interval | 0 | Findings should be interpreted with caution due to reliance on cross-sectional data. This study was also subject to monomethod and self report bias as all measures were nurse reported and collected via online survey. Another limitation was the use of nested data without the use of multilevel modeling. Using single hospital limited the generalizability. | 2 | Consistency | 2 | Important for Saudi hospital administrators and nurse leaders | 15 |
| 6 | Extensive Evaluation of the Overall Workplace Experience and Job Satisfaction of Dentists in Saudi Arabia | [file:///C:/Users/mtok1/OneDrive/%D8%B3%D8%B7%D8%AD%20%D8%A7%D9%84%D9%85%D9%83%D8%AA%D8%A8/Included%20Articles/A.%20A.%20Assiry%20-%20Extensive%20Evaluation%20of%20the%20Overall%20Workplace%20Experience%20and%20Job%20Satisfaction%20of%20Dentists%20in%20Saudi%20Arabia.pdf](file:///C:\Users\mtok1\OneDrive\Ø³Ø·Ø­%20Ø§ÙÙÙØªØ¨\Included%20Articles\A.%20A.%20Assiry%20-%20Extensive%20Evaluation%20of%20the%20Overall%20Workplace%20Experience%20and%20Job%20Satisfaction%20of%20Dentists%20in%20Saudi%20Arabia.pdf) | 2 | To determining the factors influencing the job satisfaction level among dentist and evaluating how personal and professional characteristics influence overall job satisfaction. | 1 | Convenience sample | YES | 2 | A structured self-administered questionnaire including 2 parts: information on personal characteristics and a questionnaire on job satisfaction | 2 | SPSS, means and standard deviations, multiple linear regression analysis, and binary logistic regression analysis | 2 | Found positive satisfaction with fellow workers and colleagues. Negative satisfaction with income | 2 | Used 95% confidence interval | 2 | sample size was 155 but it was specified beforehand that only completed responses would be accepted, the responses were gathered by sending reminders, but previous literature has shown that health professionals usually offer fewer questionnaire surveys, cross-sectional design and Convenience sampling was used for this study, data were obtained from different regions to ensure the generalizability of the study's findings. | 2 | Consistency | 0 | N/A | 15 |
| 7 | Healthy work environments are critical for nurse job satisfaction: implications for Oman | [file:///C:/Users/mtok1/OneDrive/%D8%B3%D8%B7%D8%AD%20%D8%A7%D9%84%D9%85%D9%83%D8%AA%D8%A8/Included%20Articles/A.%20Albashayreh%20-%20Healthy%20work%20environments%20are%20critical%20for%20nurse%20job%20satisfaction_%20implications%20for%20Oman.pdf](file:///C:\Users\mtok1\OneDrive\Ø³Ø·Ø­%20Ø§ÙÙÙØªØ¨\Included%20Articles\A.%20Albashayreh%20-%20Healthy%20work%20environments%20are%20critical%20for%20nurse%20job%20satisfaction_%20implications%20for%20Oman.pdf) | 2 | To examine the level and variability of nurse work environment and job satisfaction and explore how nurse job satisfaction is influenced by the nurses' characteristics and work environment. | 1 | convenient sample | YES | 2 | a self-administered questionnaire including 3 parts: background variables, nurse work environment (NEW), and nurse job satisfaction (NJS) | 2 | descriptive statistics, t-test and one-way ANOVA with Tukey's (HSD), Pearson product moment correlation, and hierarchical regression | 2 | Found positive satisfaction with work environment | 2 | Used 95% confidence interval | 0 | convenient sample, although we successfully recruited both local and expatriate nurses, there may have been differences between respondents' characteristics compared to the study population, limiting generalizability of our finding, didn’t consider the influence of time and length of the work shift on NJS making it difficult to precisely test the influence of these factors. | 2 | Consistency | 2 | Important for nursing and health policy | 15 |
| 8 | Job satisfaction of nurses in a regional hospital in Oman: A cross-sectional survey | [file:///C:/Users/mtok1/OneDrive/%D8%B3%D8%B7%D8%AD%20%D8%A7%D9%84%D9%85%D9%83%D8%AA%D8%A8/Included%20Articles/Job%20Satisfaction%20of%20Nurses%20in%20a%20Regional%20Hospital%20in%20Oman%20A%20Cross-Sectional%20Survey.pdf](file:///C:\Users\mtok1\OneDrive\Ø³Ø·Ø­%20Ø§ÙÙÙØªØ¨\Included%20Articles\Job%20Satisfaction%20of%20Nurses%20in%20a%20Regional%20Hospital%20in%20Oman%20A%20Cross-Sectional%20Survey.pdf) | 2 | To measure the job satisfaction of nurses working at a regional hospital in Oman and determine the factors that most significantly influence this satisfaction. | 2 | stratified random sampling | YES | 2 | McCloskey/Mueller Satisfaction Scale (MMSS) and sociodemographic | 2 | SPSS, descriptive statistics, Mult regression analysis, and one-way analysis of variance | 2 | Found positive satisfaction with your nursing peers, your amount of responsibility, your control over work conditions, salary, opportunities to write and publish, and opportunity to participate in nursing research. | 2 | Used 95% confidence interval | 0 | N/A | 2 | Consistency | 2 | Important for Ministry of Health and policy decision makers | 16 |
| 9 | Onsite Versus Remote Working: The Impact on Satisfaction, Productivity, and Performance of Medical Call Centre Workers | [file:///C:/Users/mtok1/OneDrive/%D8%B3%D8%B7%D8%AD%20%D8%A7%D9%84%D9%85%D9%83%D8%AA%D8%A8/Included%20Articles/A.%20Alfaleh%20-%20Onsite%20Versus%20Remote%20Working_%20The%20Impact%20on%20Satisfaction,%20Productivity,%20and%20Performance%20of%20Medical%20Call%20Center%20Workers.pdf](file:///C:\Users\mtok1\OneDrive\Ø³Ø·Ø­%20Ø§ÙÙÙØªØ¨\Included%20Articles\A.%20Alfaleh%20-%20Onsite%20Versus%20Remote%20Working_%20The%20Impact%20on%20Satisfaction,%20Productivity,%20and%20Performance%20of%20Medical%20Call%20Center%20Workers.pdf) | 2 | To determine the role of remote call center working on agents ' satisfaction compared to onsite working. | 2 | random sample | YES | 2 | A predesigned questionnaire consists of 6 sections: socio-demographic data, data about physician' satisfaction, an open-ended question regarding the physicians' suggestions regarding improvement of their satisfaction and performance | 0 | Welch's unpaired t-test, chi-square test, and Mann-Whitney U test were performed by using SPSS | 2 | Onsite agents were more satisfied concerning job nature, supervisor support, productivity, and performance compared to remote agent. Onesite agents were less satisfied regarding job autonomy than remote physicians. Checking again for the effect percentage | 2 | Used 95% confidence interval | 0 | N/A | 2 | Consistency | 2 | Important for organizations | 14 |
| 10 | Job satisfaction and stress among healthcare workers in public hospitals in Qatar | [file:///C:/Users/mtok1/OneDrive/%D8%B3%D8%B7%D8%AD%20%D8%A7%D9%84%D9%85%D9%83%D8%AA%D8%A8/Included%20Articles/A.%20Yehya%20-%20Job%20satisfaction%20and%20stress%20among%20healthcare%20workers%20in%20public%20hospitals%20in%20Qatar.pdf](file:///C:\Users\mtok1\OneDrive\Ø³Ø·Ø­%20Ø§ÙÙÙØªØ¨\Included%20Articles\A.%20Yehya%20-%20Job%20satisfaction%20and%20stress%20among%20healthcare%20workers%20in%20public%20hospitals%20in%20Qatar.pdf) | 2 | To assess the prevalence and predictor of job dissatisfaction among our diverse employees, most of whom were full time employees | 2 | random sample | YES | 2 | sociodemographic data followed by the National Institute for Occupational Safety and Health (NIOSH) generic Job Stress questionnaire. | 2 | Means and standards deviations, frequency and percentages, Cronbach's alpha, Pearson's correlation, SPSS, simple regression analysis, and linear regression. | 2 | Negative satisfaction with role ambiguity, conflict, skill underutilization and workload | 2 | Used 95% confidence interval | 0 | Most of the sample was females and we found that gender was associated with job-dissatisfaction. It also had more nurse than physician participants. | 2 | Consistency | 0 | N/A | 14 |
| 11 | An assessment of job satisfaction: A cross-sectional study among orthodontists of Saudi Arabia | [file:///C:/Users/mtok1/OneDrive/%D8%B3%D8%B7%D8%AD%20%D8%A7%D9%84%D9%85%D9%83%D8%AA%D8%A8/Included%20Articles/An%20assessment%20of%20job%20satisfaction%20A%20cross-sectional%20study%20among%20orthodontists%20of%20Saudi%20Arabia.pdf](file:///C:\Users\mtok1\OneDrive\Ø³Ø·Ø­%20Ø§ÙÙÙØªØ¨\Included%20Articles\An%20assessment%20of%20job%20satisfaction%20A%20cross-sectional%20study%20among%20orthodontists%20of%20Saudi%20Arabia.pdf) | 2 | To investigate the level of job satisfaction among professional orthodontist in relation to some significant intrinsic and extrinsic factors that generally affect their performance. | 2 | random sample | YES | 2 | Job satisfaction survey including significant intrinsic and extrinsic factors, and sociodemographic characteristics | 0 | Descriptive analysis, SPSS, Means, standard deviations, percentages, and t-test | 2 | Positive satisfaction with their job as orthodontists, resources, facilities, staff, workplace environment, working as a team, resources, facilities, staff, workplace environment, attitude and respect by patient Negative satisfaction with income and not having adequate time for their families, administrative duties and paperwork. | 2 | Used 95% confidence interval | 0 | N/A | 2 | Consistency | 0 | N/A | 12 |
| 12 | Analysis of occupational stress, burnout, and job satisfaction among dental practitioners | [file:///C:/Users/mtok1/OneDrive/%D8%B3%D8%B7%D8%AD%20%D8%A7%D9%84%D9%85%D9%83%D8%AA%D8%A8/Included%20Articles/Analysis%20of%20occupational%20stress,%20burnout,%20and%20job%20satisfaction%20among%20dental%20practitioners.pdf](file:///C:\Users\mtok1\OneDrive\Ø³Ø·Ø­%20Ø§ÙÙÙØªØ¨\Included%20Articles\Analysis%20of%20occupational%20stress,%20burnout,%20and%20job%20satisfaction%20among%20dental%20practitioners.pdf) | 2 | To investigate dimensions of occupational stress among dental practitioners leading to burnout and to model its causality with job satisfaction. | 1 | consecutive sample technique | YES | 2 | Included socioeconomic | 2 | Univariate and multivariate analysis | 2 | Found Occupational stress was significantly associated with burnout and job dissatisfaction in dentistry | 2 | Used 95% confidence interval | 1 | Applied to consecutive sample of dental practitioners only | 2 | Consistency | 2 | Increased stress level affects the quality of treatment | 16 |
| 13 | JOB SATISFACTION AND INTENTION TO LEAVE AMONG CRITICAL CARE NURSES IN SAUDI ARABIA | [file:///C:/Users/mtok1/OneDrive/%D8%B3%D8%B7%D8%AD%20%D8%A7%D9%84%D9%85%D9%83%D8%AA%D8%A8/Included%20Articles/JOB%20SATISFACTION%20AND%20INTENTION%20TO%20LEAVE%20AMONG%20CRITICAL%20CARE%20NURSES%20IN%20SAUDI%20ARABIA.pdf](file:///C:\Users\mtok1\OneDrive\Ø³Ø·Ø­%20Ø§ÙÙÙØªØ¨\Included%20Articles\JOB%20SATISFACTION%20AND%20INTENTION%20TO%20LEAVE%20AMONG%20CRITICAL%20CARE%20NURSES%20IN%20SAUDI%20ARABIA.pdf) | 2 | To examine relationship between registered nurses’(RN)job satisfaction and their intention to leave critical care nursing in Saudi Arabia. | 1 | convenience  sample | YES | 2 | Included socioeconomic | 2 | Bivariate and multivariable analysis | 2 | 3 dimensions of JS perceived workload, professional support, and pay and prospects for promotion were found to be associated with intention to leave. | 2 | Used 95% confidence interval | 0 | Convenience sampling from a single public hospital | 2 | Consistency | 2 | Important for critical care managers and policy makers | 15 |
| 14 | The factors influencing burnout and job satisfaction among critical care nurses: a study of Saudi critical care nurses | [file:///C:/Users/mtok1/OneDrive/%D8%B3%D8%B7%D8%AD%20%D8%A7%D9%84%D9%85%D9%83%D8%AA%D8%A8/Included%20Articles/J.%20Alharbi%20-%20The%20factors%20influencing%20burnout%20and%20job%20satisfaction%20among%20critical%20care%20nurses_%20a%20study%20of%20Saudi%20critical%20care%20nurses.pdf](file:///C:\Users\mtok1\OneDrive\Ø³Ø·Ø­%20Ø§ÙÙÙØªØ¨\Included%20Articles\J.%20Alharbi%20-%20The%20factors%20influencing%20burnout%20and%20job%20satisfaction%20among%20critical%20care%20nurses_%20a%20study%20of%20Saudi%20critical%20care%20nurses.pdf) | 2 | To explore the prevalence of burnout and job satisfaction among Saudi national critical care nurses. | 1 | convenience  sample | YES | 2 | Included socioeconomic | 1 | Bivariate analysis | 2 | dissatisfied with contingent rewards and operating conditions but satisfied with the nature of their work. | 2 | Used 95% confidence interval | 0 | Convenience sampling in three hospitals within one city. | 2 | Consistency | 2 | Important for nurse managers and policy makers | 14 |
| 15 | Effect of leadership styles on job satisfaction among critical care nurses in Aseer, Saudi Arabia | [file:///C:/Users/mtok1/OneDrive/%D8%B3%D8%B7%D8%AD%20%D8%A7%D9%84%D9%85%D9%83%D8%AA%D8%A8/Included%20Articles/Effect%20of%20leadership%20styles%20on%20job%20satisfaction%20among%20critical%20care%20nurses%20in%20Aseer,%20Saudi%20Arabia.pdf](file:///C:\Users\mtok1\OneDrive\Ø³Ø·Ø­%20Ø§ÙÙÙØªØ¨\Included%20Articles\Effect%20of%20leadership%20styles%20on%20job%20satisfaction%20among%20critical%20care%20nurses%20in%20Aseer,%20Saudi%20Arabia.pdf) | 2 | To evaluate the effect of transformational and transactional leadership styles of head nurses on the job satisfaction of staff nurses in critical care units (CCU) of a tertiary care hospital. | 1 | convenience  sample | YES | 2 | Included socioeconomic | 2 | Multiple linear regression analysis | 2 | satisfied in work environment, transformational leadership style, but dissatisfied in operating conditions, fringe benefit, contingent rewards | 2 | Used 95% confidence interval | 0 | convenience sample of nurses (CCU) from a single tertiary hospital | 2 | Consistency | 2 | Important for nurses’ leaders to be trained in TF style | 15 |
| 16 | JOB RELATED STRESSORS AND JOB SATISFACTION AMONG MULTICULTURAL NURSING WORkFORCE | [file:///C:/Users/mtok1/OneDrive/%D8%B3%D8%B7%D8%AD%20%D8%A7%D9%84%D9%85%D9%83%D8%AA%D8%A8/Included%20Articles/JOB%20RELATED%20STRESSORS%20AND%20JOB%20SATISFACTION%20AMONG%20MULTICULTURAL%20NURSING%20WORkFORCE.pdf](file:///C:\Users\mtok1\OneDrive\Ø³Ø·Ø­%20Ø§ÙÙÙØªØ¨\Included%20Articles\JOB%20RELATED%20STRESSORS%20AND%20JOB%20SATISFACTION%20AMONG%20MULTICULTURAL%20NURSING%20WORkFORCE.pdf) | \| 2 \| \| --- \| | To identify the different job-related stressors in hospital that affect nurses' job satisfaction from a multicultural perspective and examine how these two variables were correlated with each other. | 1 | convenience sample | YES | 2 | Included socioeconomic | 0 | correlational analysis | 2 | Found satisfaction positive regarding nature of work and operating condition. Dissatisfied regarding pay and fringe benefit. | 2 | Used 95% confidence interval | 0 | convenience sample in one facility, sample is not homogenous in gender. | 2 | Consistency | 2 | Administration | 13 |
| 17 | Effect of Occupational Stress on Pharmacists’ Job Satisfaction in Saudi Arabia | [file:///C:/Users/mtok1/OneDrive/%D8%B3%D8%B7%D8%AD%20%D8%A7%D9%84%D9%85%D9%83%D8%AA%D8%A8/Included%20Articles/Effect%20of%20Occupational%20Stress%20on%20Pharmacists%E2%80%99%20Job%20Satisfaction%20in%20Saudi%20Arabia.pdf](file:///C:\Users\mtok1\OneDrive\Ø³Ø·Ø­%20Ø§ÙÙÙØªØ¨\Included%20Articles\Effect%20of%20Occupational%20Stress%20on%20Pharmacistsâ%20Job%20Satisfaction%20in%20Saudi%20Arabia.pdf) | 2 | To assess the impact of work stress on job satisfaction among pharmacists in Saudi Arabia. | 1 | Convenient sample | YES | 2 | Included socioeconomic, occupational stress, job satisfaction, and expectation assessment | 2 | Univariate and multivariate regressions analysis | 2 | Found positive relationship with job satisfaction on confirmation, age, male gender. High education, married. Negative relationship on occupational stress and working in hospital. | 2 | Used 95% confidence interval | 1 | generalizable to only a similar population | 2 | Consistency | 0 | Nothing | 14 |
| 18 | Evaluating pharmacists' motivation and job satisfaction factors in Saudi Hospitals | [file:///C:/Users/mtok1/OneDrive/%D8%B3%D8%B7%D8%AD%20%D8%A7%D9%84%D9%85%D9%83%D8%AA%D8%A8/Included%20Articles/Evaluating%20pharmacists'%20motivation%20and%20job%20satisfaction%20factors%20in%20Saudi%20Hospitals.pdf](file:///C:\Users\mtok1\OneDrive\Ø³Ø·Ø­%20Ø§ÙÙÙØªØ¨\Included%20Articles\Evaluating%20pharmacists'%20motivation%20and%20job%20satisfaction%20factors%20in%20Saudi%20Hospitals.pdf) | 2 | To assess the level of job satisfaction and factors that motivate pharmacists in four different hospitals in Saudi Arabia | 0 |  | YES | 2 | Including demographic, professional information, characteristics | 0 | N/A | 2 | Found satisfaction on the relationship between workers, with human resource support, dissatisfied with the incentives | 2 | Used 95% confidence interval | 0 | N/A | 2 | consistency | 2 | Important for pharmacy manager | 12 |
| 19 | Expatriate health professionals in the Saudi Arabia private sector | [file:///C:/Users/mtok1/OneDrive/%D8%B3%D8%B7%D8%AD%20%D8%A7%D9%84%D9%85%D9%83%D8%AA%D8%A8/Included%20Articles/ExpatriateHealthProfessionalsintheSaudiPrivateSectorJobsatisfactionandturnoverintention.pdf](file:///C:\Users\mtok1\OneDrive\Ø³Ø·Ø­%20Ø§ÙÙÙØªØ¨\Included%20Articles\ExpatriateHealthProfessionalsintheSaudiPrivateSectorJobsatisfactionandturnoverintention.pdf) | 2 | identify the stressors that affect job satisfaction among different EHPs, and the relationship between job satisfaction and turnover intention among EHPs in Saudi private hospitals. | 1 | convenience sample | YES | 2 | Including demographic, Herzberg's Motivation-Hygiene Theory and our study's conceptual framework. | 2 | multiple stepwise regressions | 2 | Found positive satisfaction on working conditions, supervision, salary, interpersonal relationships, and hospital policy and administration, job security, cultural unrest. | 2 | Used 95% confidence interval | 0 | Applied convenience sample can affect generalisability of the findings due to sampling bias. | 2 | Consistency | 2 | Important for administrators, researchers, and policy makers. | 15 |
| 20 | The impact of leadership styles on nurses' satisfaction and intention to stay among Saudi nurses | [file:///C:/Users/mtok1/OneDrive/%D8%B3%D8%B7%D8%AD%20%D8%A7%D9%84%D9%85%D9%83%D8%AA%D8%A8/Included%20Articles/R.%20F.%20Abualrub%20-%20The%20impact%20of%20leadership%20styles%20on%20nurses%E2%80%99%20satisfaction%20and%20intention%20to%20stay%20among%20Saudi%20nurses.pdf](file:///C:\Users\mtok1\OneDrive\Ø³Ø·Ø­%20Ø§ÙÙÙØªØ¨\Included%20Articles\R.%20F.%20Abualrub%20-%20The%20impact%20of%20leadership%20styles%20on%20nursesâ%20satisfaction%20and%20intention%20to%20stay%20among%20Saudi%20nurses.pdf) | 2 | To examine the impact of leadership styles of nurse managers on Saudi nurses' job satisfaction and their intention to stay at work. | 1 | convenience sample | YES | 2 | Multifactor leadership questionnaire, job satisfaction survey, the McCain's Intent to stay Scale, and the demographic form | 2 | multiple regression | 2 | Found more satisfied with nature of work and lest satisfied with fringe benefit. Positive between transformational leadership style and nurses job satisfaction, weak positive between nurses' job satisfaction and nurses' intention to leave, but negative with transactional leadership style and job satisfaction. | 2 | Used 95% confidence interval | 0 | Using convenience sample from 6 public hospitals, and the collected data relied on the recall of participants which might create a reporting bias. | 2 | Consistency | 2 | Important for nurse managers | 15 |
| 21 | Job Satisfaction of UA.E. Dental Practitioners | [file:///C:/Users/mtok1/OneDrive/%D8%B3%D8%B7%D8%AD%20%D8%A7%D9%84%D9%85%D9%83%D8%AA%D8%A8/Included%20Articles/F.%20S.%20Al-Buainain%20-%20Job%20Satisfaction%20of%20U.A.E.%20Dental%20Practitioners.pdf](file:///C:\Users\mtok1\OneDrive\Ø³Ø·Ø­%20Ø§ÙÙÙØªØ¨\Included%20Articles\F.%20S.%20Al-Buainain%20-%20Job%20Satisfaction%20of%20U.A.E.%20Dental%20Practitioners.pdf) | 2 | To assess the overall job satisfaction among working dentists in the UAE and to assess the effect of sociodemographic factors and work environment factors on their level of satisfaction. | 0 |  | YES | 2 | A survey modified from the ADA version of job satisfaction includes 4 sections in addition to demographic factors questions. | 2 | multiple regression analysis | 2 | Found dentists were more satisfied with their relationship with the patients, other colleagues, and staff. They were least satisfied with the opportunity for part time work followed by their perception of benefit packages related to insurance, retirement, and bonuses. | 2 | Used 95% confidence interval | 0 | limitation of this study includes the relatively small sample size. | 2 | Consistency | 2 | Important to policy makers | 14 |
| 22 | Determinants of rehabilitation services staffs' job satisfaction (by Effort Reward Imbalance) and variations in teaching, profit making and nonprofit hospitals | [file:///C:/Users/mtok1/OneDrive/%D8%B3%D8%B7%D8%AD%20%D8%A7%D9%84%D9%85%D9%83%D8%AA%D8%A8/Included%20Articles/I.%20Devreux%20-%20Determinants%20of%20Rehabilitation%20Services%20Staffs'%20Job%20Satisfaction%20(By%20Effort%20Reward%20Imbalance)%20and%20Variations%20in%20Teaching,%20Profit%20Making%20and%20Non%20Profit%20Hospitals.pdf](file:///C:\Users\mtok1\OneDrive\Ø³Ø·Ø­%20Ø§ÙÙÙØªØ¨\Included%20Articles\I.%20Devreux%20-%20Determinants%20of%20Rehabilitation%20Services%20Staffs'%20Job%20Satisfaction%20(By%20Effort%20Reward%20Imbalance)%20and%20Variations%20in%20Teaching,%20Profit%20Making%20and%20Non%20Profit%20Hospitals.pdf) | 2 | To identify components of job satisfaction based on the effort and reward imbalance model and compare the job satisfaction level of employees working in the different types of healthcare facilities in the Jeddah area. | 2 | random sample | YES | 2 | Including demographic, effort and reward imbalance, practice of work, job satisfaction | 0 | Descriptive statistic and correlations analysis | 2 | Found positive satisfaction in different types of hospitals, such as salary, perception of the salary, yearly performance appraisal and effective commitment score, patient/staff, feeling emotionally attached to the organization, doctors respect, involvement in quality improvement or educational lectures and transportation modalities. | 2 | Used 95% confidence interval | 0 | N/A | 2 | Consistency | 0 | N/A | 12 |
| 23 | Measurement of rehabilitation services staffs' job satisfaction using the Effort Reward Imbalance Model in Saudi Arabia | [file:///C:/Users/mtok1/OneDrive/%D8%B3%D8%B7%D8%AD%20%D8%A7%D9%84%D9%85%D9%83%D8%AA%D8%A8/Included%20Articles/I.%20Devreux%20-%20Measurement%20of%20Rehabilitation%20Services%20Staffs'%20Job%20Satisfaction%20Using%20the%20Effort%20Reward%20Imbalance%20Model%20in%20Saudi%20Arabia.pdf](file:///C:\Users\mtok1\OneDrive\Ø³Ø·Ø­%20Ø§ÙÙÙØªØ¨\Included%20Articles\I.%20Devreux%20-%20Measurement%20of%20Rehabilitation%20Services%20Staffs'%20Job%20Satisfaction%20Using%20the%20Effort%20Reward%20Imbalance%20Model%20in%20Saudi%20Arabia.pdf) | 2 | To evaluate the level of work satisfaction of staff working in rehabilitation services based on the Effort-Reward Imbalance Model. | 2 | random sample | YES | 2 | including sociodemographic, evaluating ERI ratio. | 0 | Sperman correlation analysis | 2 | Found higher effort reward imbalance ratio in Filipino nationals, respiratory therapists, personnel working exclusively with inpatients, having split duties, and person has Master or PhD degree. | 2 | Used 95% confidence interval | 0 | N/A | 2 | Consistency | 2 | Important to human resource management | 14 |
| 24 | Interprofessional collaboration as a mediator in the relationship between nurse work environment, patient safety outcomes and job satisfaction among nurses | [file:///C:/Users/mtok1/OneDrive/%D8%B3%D8%B7%D8%AD%20%D8%A7%D9%84%D9%85%D9%83%D8%AA%D8%A8/Included%20Articles/Interprofessional%20collaboration%20as%20a%20mediator%20in%20the%20relationship%20between%20nurse%20work%20environment,%20patient%20safety%20outcomes%20and%20job%20satisfaction%20among%20nurses.pdf](file:///C:\Users\mtok1\OneDrive\Ø³Ø·Ø­%20Ø§ÙÙÙØªØ¨\Included%20Articles\Interprofessional%20collaboration%20as%20a%20mediator%20in%20the%20relationship%20between%20nurse%20work%20environment,%20patient%20safety%20outcomes%20and%20job%20satisfaction%20among%20nurses.pdf) | 2 | To assess the contribution of the work environment to patient safety outcomes and job satisfaction, with interprofessional collaboration as a mediating factor. | 0 | Not mentioned | YES | 2 | including sociodemographic, practice environment scale of the nursing work index, assessment for collaborative environment tool, and two single-item measures for nursing care quality and job satisfaction. | 2 | multiple regression analysis | 2 | Found positive correlated with Nurse work environment and interprofessional collaboration | 2 | Used 95% confidence interval | 1 | using cross sectional, utilization of nurse self-report approach to measure are quality, inclusion of nurses from other Governorates in the country may yield a more generalizable finding. | 2 | Consistency | 0 | N/A | 13 |
| 25 | Nursing Work Environment, Turnover Intention, Job Burnout, and Quality of Care: The Moderating Role of Job Satisfaction | [file:///C:/Users/mtok1/OneDrive/%D8%B3%D8%B7%D8%AD%20%D8%A7%D9%84%D9%85%D9%83%D8%AA%D8%A8/Included%20Articles/Nursing%20Work%20Environment,%20Turnover%20Intention,%20Job%20Burnout,%20and%20Quality%20of%20Care%20The%20Moderating%20Role%20of%20Job%20Satisfaction.pdf](file:///C:\Users\mtok1\OneDrive\Ø³Ø·Ø­%20Ø§ÙÙÙØªØ¨\Included%20Articles\Nursing%20Work%20Environment,%20Turnover%20Intention,%20Job%20Burnout,%20and%20Quality%20of%20Care%20The%20Moderating%20Role%20of%20Job%20Satisfaction.pdf) | 2 | To assess predictors of the turnover intention, burnout, and perceived quality of care among nurses working in Oman and to examine the potential moderating role of job satisfaction on the relationship between work environment and nurse turnover intention. | 1 | convenience sample | YES | 2 | structured survey included demographics, Practice Environment Scale of the Nursing Work Index (PES-NWI), emotional exhaustion (EE) subscale of the Maslach Burnout Inventory (MBI), and Job Satisfaction Scale. | 2 | Logistic regression analysis, descriptive and bivariate statistics, multivariate logistic regression model | 2 | Found negative relationship between nurses' job satisfaction and their turnover intention | 2 | Used 95% confidence interval | 0 | using cross sectional design to find the causal relationship between work environment and turnover intention, burnout, and perceived quality of care isn't possible. Small sample size and low response rate in one site. Also, response rate is lower than previous studies. The sample constitute around 5% of the total nurses in the country. using convenience sample in one hospital may introduce of bias. the fact that the various questionnaire used had somewhat dissimilar foci. using of emotional exhaustion as a subscale from the burnout instrument because reliability cannot be directly related to the reliability of the total instrument. The fact that the various questionnaires used had somewhat dissimilar foci. | 2 | Consistency | 0 | N/A | 13 |
| 26 | Job Motivation and Satisfaction Among Female Pharmacists Working in Private Pharmacy Professional Sectors in Saudi Arabia | [file:///C:/Users/mtok1/OneDrive/%D8%B3%D8%B7%D8%AD%20%D8%A7%D9%84%D9%85%D9%83%D8%AA%D8%A8/Included%20Articles/Job%20Motivation%20and%20Satisfaction%20Among%20Female%20Pharmacists%20Working%20in%20Private%20Pharmacy%20Professional%20Sectors%20in%20Saudi%20Arabia.pdf](file:///C:\Users\mtok1\OneDrive\Ø³Ø·Ø­%20Ø§ÙÙÙØªØ¨\Included%20Articles\Job%20Motivation%20and%20Satisfaction%20Among%20Female%20Pharmacists%20Working%20in%20Private%20Pharmacy%20Professional%20Sectors%20in%20Saudi%20Arabia.pdf) | 2 | To investigate the perceived motivational factors and assessed the level of job satisfaction of female pharmacists working in private pharmaceutical sectors. | 1 | convenience sample | YES | 2 | self administered online questionnaire. It consisted of 3 sections: related to sociodemographic, workplace, and job characteristics, related to job motivation, and related to participants' job satisfaction. | 2 | univariable and multiple linear regression | 2 | Found negative satisfaction for non-Saudi, pert time pharmacists who never expected promotions. female pharmacists derive the highest satisfaction from their colleagues, having attention paid to their suggestions, having opportunities to use their abilities, enjoying good relationships with their managers and co-workers, and given a good amount of responsibility. full time workers. | 2 | Used 95% confidence interval | 0 | convenience sampling, selecting only available participants. Most of e participant were young females working in private sector without including senior level female pharmacists who work in other settings. Lack of similar research in order to make the comparison. | 2 | Consistency | 2 | Important for pharmaceutical firms' leaders | 15 |
| 27 | Job Satisfaction among Nurses Working at Primary Health Centre in Ras Al Khaimah, United States Emirates | [file:///C:/Users/mtok1/OneDrive/%D8%B3%D8%B7%D8%AD%20%D8%A7%D9%84%D9%85%D9%83%D8%AA%D8%A8/Included%20Articles/Job%20Satisfaction%20among%20Nurses%20Working%20at%20Primary%20Health%20Center%20in%20Ras%20Al%20Khaimah,%20United%20States%20Emirates.pdf](file:///C:\Users\mtok1\OneDrive\Ø³Ø·Ø­%20Ø§ÙÙÙØªØ¨\Included%20Articles\Job%20Satisfaction%20among%20Nurses%20Working%20at%20Primary%20Health%20Center%20in%20Ras%20Al%20Khaimah,%20United%20States%20Emirates.pdf) | 2 | identifying the level of job satisfaction among nurses and factors influencing the satisfaction levels among nurses in primary health centre in Ras Al Khaimah. | 1 | convenience sample | YES | 2 | 4 sections of questionnaire-demographic variable, measure of job satisfaction, extrinsic and intrinsic factors | 0 | descriptive and inferential statistics | 2 | found positive satisfaction with respect from management, feedback given, and group cohesion. Negative satisfaction with not being paid fairly, recognition of senior nurses, disruptions in social life due to working hours, workload. | 2 | Used 95% confidence interval | 0 | N/A | 0 | N/A | 2 | Important for policy makers and practice leaders | 11 |
| 28 | Job satisfaction among physiotherapists in Saudi Arabia: does the leadership style matter? | [file:///C:/Users/mtok1/OneDrive/%D8%B3%D8%B7%D8%AD%20%D8%A7%D9%84%D9%85%D9%83%D8%AA%D8%A8/Included%20Articles/Job%20satisfaction%20among%20physiotherapists%20in%20Saudi%20Arabia%20does%20the%20leadership%20style%20matter.pdf](file:///C:\Users\mtok1\OneDrive\Ø³Ø·Ø­%20Ø§ÙÙÙØªØ¨\Included%20Articles\Job%20satisfaction%20among%20physiotherapists%20in%20Saudi%20Arabia%20does%20the%20leadership%20style%20matter.pdf) | 2 | examine the job satisfaction and influential factors among physiotherapists working in private and government hospitals of Saudi Arabia with a focus on leadership style. | 0 | Not mentioned | YES | 2 | self reported questionnaire, job satisfaction survey (JSS), multifactor leadership questionnaire(MLQ), and questionnaire including demographic and work related variable. | 1 | descriptive statistic, chi square, and spearman correlation analysis. | 1 | Job satisfaction was scored as ambivalent. “slightly agreed" for immediate supervision, co-workers, and the nature of work. However, "slightly disagreed" in pay, promotion, fringe benefits, contingent reward, operation conditions, and communication | 2 | Used 95% confidence interval | 0 | small sample size, response rate was low with respect to the number of email sent, and the length of the survey was large that could produce low response as it could be difficult for therapists to complete it within their busy schedules. | 2 | Consistency | 0 | N/A | 10 |
| 29 | Predictors of global job satisfaction among Saudi physiotherapists: a descriptive study | [file:///C:/Users/mtok1/OneDrive/%D8%B3%D8%B7%D8%AD%20%D8%A7%D9%84%D9%85%D9%83%D8%AA%D8%A8/Included%20Articles/Predictors%20of%20global%20job%20satisfaction%20among%20Saudi%20physiotherapists%20a%20descriptive%20study.pdf](file:///C:\Users\mtok1\OneDrive\Ø³Ø·Ø­%20Ø§ÙÙÙØªØ¨\Included%20Articles\Predictors%20of%20global%20job%20satisfaction%20among%20Saudi%20physiotherapists%20a%20descriptive%20study.pdf) | 2 | investigate the level of job satisfaction and to identify predictors of job satisfaction among Saudi PTs. | 1 | stratified sample | YES | 2 | purpose-designed job satisfaction questionnaire. Final survey consisted of general demographic data and 32 items across 8 domains. | 1 | Linear regressions analysis and statistical analysis | 2 | Found positive satisfaction with professional development and teamwork. Negative satisfaction with supervisory/management relationship and working environment. | 2 | Used 95% confidence interval | 0 | it possible occur sample bias because PTs who were either satisfied or dissatisfied with their job may have chosen to participate, cross sectional design used in the study, we did not attempt to control the day of the week on which the survey was completed, this may have affected the results, as job satisfaction has been found to be higher on Friday than in the middle of the week. The authors didn't take into consideration the individual work environment. Personal factors such as accessibility to work, marital status, and socioeconomic statue would have affected their job satisfaction. | 2 | Consistency | 2 | Important for managers and directors of PT department. | 14 |
| 30 | Job Satisfaction Among Radiologic Technologists at Hospitals in Saudi Arabia's Southern Region: A Cross-sectional Study | [file:///C:/Users/mtok1/OneDrive/%D8%B3%D8%B7%D8%AD%20%D8%A7%D9%84%D9%85%D9%83%D8%AA%D8%A8/Included%20Articles/Job%20Satisfaction%20Among%20Radiologic%20Technologists%20at%20Hospitals%20in%20Saudi%20Arabia's%20Southern%20Region%20A%20Cross-sectional%20Study.pdf](file:///C:\Users\mtok1\OneDrive\Ø³Ø·Ø­%20Ø§ÙÙÙØªØ¨\Included%20Articles\Job%20Satisfaction%20Among%20Radiologic%20Technologists%20at%20Hospitals%20in%20Saudi%20Arabia's%20Southern%20Region%20A%20Cross-sectional%20Study.pdf) | 2 | To determine how satisfied with their work are the radiology technologists in the hospitals of Aseer province. | 0 | Not mentioned | YES | 2 | Minnesota Satisfaction Questionnaire (MSQ) and general demographic data. | 0 | chi-square test and SPSS | 2 | Found positive satisfaction with sense of accomplishment they received from the job, ability to do good things for other people in the society, and values within the society they live in. Negative satisfaction with work policies at their institution, ability to improve their skills and qualification at work, ability to use their judgment at work, and nature of the relationship between them and their supervisors. | 2 | Used 95% confidence interval | 0 | N/A | 0 | unconsistency | 0 | N/A | 8 |
| 31 | Unique expatriate factors associated with job dissatisfaction among nurses | [file:///C:/Users/mtok1/OneDrive/%D8%B3%D8%B7%D8%AD%20%D8%A7%D9%84%D9%85%D9%83%D8%AA%D8%A8/Included%20Articles/Unique%20expatriate%20factors%20associated%20with%20job%20dissatisfaction%20among%20nurses.pdf](file:///C:\Users\mtok1\OneDrive\Ø³Ø·Ø­%20Ø§ÙÙÙØªØ¨\Included%20Articles\Unique%20expatriate%20factors%20associated%20with%20job%20dissatisfaction%20among%20nurses.pdf) | 2 | To assess the over all job dissatisfaction among the expatriate nurses and to identify its significant correlates. | 1 | non random sample | YES | 2 | we used the strengthening the reporting of observational studied in epidemiology (STROBE) checklist as a guideline. Survey included questions on demography, job dissatisfaction, job characteristics, job duration as an expatriate, communication issues with patients and doctors, fear of litigation, and job insecurity. | 1 | hierarchical logistic regression | 2 | Found negative satisfaction with job insecurity, shorter job duration, and patient communication problems | 2 | Used 95% confidence interval | 0 | we didn't collect data on a few of the known correlates of job dissatisfaction, such as turnover intention, organizational commitment and burnout, which were identified in earlier studies, non-random sample because the survey was administered online and participation was voluntary, nurses employed in governmental hospitals/clinics only, and the cross-sectional study design prevented us from examining the temporality of the association. | 2 | Consistency | 0 | N/A | 12 |
| 32 | The effect of gender on transformational leadership and job satisfaction among Saudi nurses | [file:///C:/Users/mtok1/OneDrive/%D8%B3%D8%B7%D8%AD%20%D8%A7%D9%84%D9%85%D9%83%D8%AA%D8%A8/Included%20Articles/Alghamdi%20-%20The%20effect%20of%20gender%20on%20transformational%20leadership%20and%20job%20satisfaction.pdf](file:///C:\Users\mtok1\OneDrive\Ø³Ø·Ø­%20Ø§ÙÙÙØªØ¨\Included%20Articles\Alghamdi%20-%20The%20effect%20of%20gender%20on%20transformational%20leadership%20and%20job%20satisfaction.pdf) | 2 | To compare nurses' job satisfaction and perceptions of transformational leadership style of their manager among four different nurse/manager gender dyads in Saudi Arabia. | 1 | convenience sample | YES | 2 | Job Satisfaction Survey (JSS) and Multifactor Leadership Questionnaire (MLQ) | 0 | Descriptive analysis | 2 | Found positive satisfaction when the manager for subordinates is male | 2 | Used 95% confidence interval | 0 | The samplewas self-selected and thus does not represent all Saudi nurses, the instruments to measure job satisfaction and perceived TF leadership behaviours were developed with samples of United States nurses whose gender roles are defined differently than in Saudi Arabia, there are many more factors that influence job satisfaction other than leadership style of the manager, the statistical differences detected in this study may not translate into clinically important findings, they were not considered some factors , such as age of the nurse and manager. | 2 | consistency | 0 | N/A | 11 |
| 33 | The demographic predictors of job satisfaction among the nurses of a major public hospital in KSA | [file:///C:/Users/mtok1/AppData/Local/Microsoft/Windows/INetCache/IE/JO3WXS49/The%20demographic%20predictors%20of%20job%20satisfaction%20among%20the%20nurses%20of%20a%20major%20public%20hospital%20in%20KSA[1].pdf](file:///C:\Users\mtok1\AppData\Local\Microsoft\Windows\INetCache\IE\JO3WXS49\The%20demographic%20predictors%20of%20job%20satisfaction%20among%20the%20nurses%20of%20a%20major%20public%20hospital%20in%20KSA%5b1%5d.pdf) | 2 | To ascertain the level of job satisfaction and impact of key demographic factors on job satisfaction among Saudi and non- Saudi nurses who were working in a public hospital in Dammam. | 2 | systematic random sampling | YES | 2 | self-administered assessment including demographic characteristics and Minnesota Satisfaction Questionnaire (MSQ) | 2 | Linear regressions analysis | 2 | Found positive satisfaction with Demographic factors, such as sex (male), non-Saudi nationality, and older age. Negative satisfaction with compensation, recognition, responsibilities, working conditions, and higher salary | 2 | Used 95% confidence interval | 0 | The sample consisted of the nurses who worked at the target public hospital in Dammam, KSA, therefore this limits the generalisability of the present findings. | 2 | Consistency | 2 | Important for decision makers and health care authorities | 16 |
| 34 | Quality of life, job satisfaction and their related factors among nurses working in king Abdulaziz University Hospital, Jeddah, Saudi Arabia | [file:///C:/Users/mtok1/AppData/Local/Microsoft/Windows/INetCache/IE/AU42TPSJ/N._K._Ibrahim_-_Quality_of_life,_job_satisfaction_and_their_related_factors_among_nurses_working_in_king_Abdulaziz_University_Hospital,_Jeddah,_Saudi_Arabia[1].pdf](file:///C:\Users\mtok1\AppData\Local\Microsoft\Windows\INetCache\IE\AU42TPSJ\N._K._Ibrahim_-_Quality_of_life,_job_satisfaction_and_their_related_factors_among_nurses_working_in_king_Abdulaziz_University_Hospital,_Jeddah,_Saudi_Arabia%5b1%5d.pdf) | 2 | To assess Quality of Life, job satisfaction and their related factors among nurses working in King Abdulaziz University Hospital, Jeddah, Saudi Arabia. | 2 | stratified random sampling | YES | 2 | Self administered include personal and socio-demographic data, occurrence of major life events during past 6 months, world health organization QOL assessment instrument (WHOQOL-BREF), and Job Descriptive Index/Job in General (JDI/JIG) | 0 | Descriptive statistic | 2 | Found positive satisfaction with coworkers, supervision, work on present job, and payment, income level, increasing the years of experience more than 9 years, nonsurgical departments, working in a day shift. Negative satisfaction with opportunity of promotion, Saudi nurses, working in surgical departments, worked in alternating and night shifts. | 2 | Used 95% confidence interval | 0 | N/A | 2 | Consistency | 0 | N/A | 12 |
| 35 | Physician job satisfaction in Saudi Arabia:  insights from a tertiary hospital survey | [file:///C:/Users/mtok1/OneDrive/%D8%B3%D8%B7%D8%AD%20%D8%A7%D9%84%D9%85%D9%83%D8%AA%D8%A8/Included%20Articles/Physician%20job%20satisfaction%20in%20Saudi%20Arabia%20insights%20from%20a%20tertiary%20hospital%20survey.pdf](file:///C:\Users\mtok1\OneDrive\Ø³Ø·Ø­%20Ø§ÙÙÙØªØ¨\Included%20Articles\Physician%20job%20satisfaction%20in%20Saudi%20Arabia%20insights%20from%20a%20tertiary%20hospital%20survey.pdf) | 2 | To determine the level of job satisfaction Saudi and non-Saudi physicians of different specialities working in Riyadh, Saudi Arabia and to explore factors associated with job dissatisfaction. | 0 | Not mentioned | YES | 2 | structured questionnaire was used to collect data on a wide range of socio-demographic, practice environment characteristics and level and consequences of job satisfaction from practicing physicians | 2 | Univariate and multiple logistic regression | 2 | Found negative satisfaction in univariate analysis income satisfaction, family life affected by specialization, positive depression symptoms on the screening test. In the multiple logistic regression model, income satisfaction the only factor associated with dissatisfaction. | 2 | Used 95% confidence interval | 2 | Few previous studies conducted in Saudi Arabia focused on primary care physician, large portion of data in these studies were obtained from non-Saudi physicians, didn’t examine the inpatient load, job satisfaction is not a single domain, measuring satisfaction with one question does not provide accurate results, the study was limited to a single tertiary institution. our study addressed the limitation of the other local studies, and the findings are generalizable to the larger Saudi physician community. | 2 | Consistency | 0 | N/A | 14 |
| 36 | Level and determinants of job satisfaction among Saudi physicians working in primary health-care facilities in Western Region, KSA | [file:///C:/Users/mtok1/AppData/Local/Microsoft/Windows/INetCache/IE/AU42TPSJ/Level_and_determinants_of_job_satisfaction_among_Saudi_physicians_working_in_primary_health-care_facilities_in_Western_Region,_KSA[1].pdf](file:///C:\Users\mtok1\AppData\Local\Microsoft\Windows\INetCache\IE\AU42TPSJ\Level_and_determinants_of_job_satisfaction_among_Saudi_physicians_working_in_primary_health-care_facilities_in_Western_Region,_KSA%5b1%5d.pdf) | 2 | To assess the level of job satisfaction and factors contributing to dissatisfaction of Saudi physicians in primary health care (PHC) centres in Jeddah. | 0 | Not mentioned | YES | 2 | A self administered of informed consent, demographic data, and job satisfaction survey (JSS) questionnaire | 0 | Descriptive statistic | 2 | Found positive satisfaction with nature of work. Negative satisfaction with promotions, contingent rewards, fringe benefits, and operating conditions. | 2 | Used 95% confidence interval | 0 | N/A | 2 | Consistency | 2 | Important to policy makers | 12 |
| 37 | Job Satisfaction of Staff Working with Individuals with Intellectual Disabilities in Two Different Settings | [file:///C:/Users/mtok1/OneDrive/%D8%B3%D8%B7%D8%AD%20%D8%A7%D9%84%D9%85%D9%83%D8%AA%D8%A8/Included%20Articles/Job%20satisfaction%20of%20staff%20working%20with%20individuals%20with%20intellectual%20disabilities%20in%20two%20different%20settings.pdf](file:///C:\Users\mtok1\OneDrive\Ø³Ø·Ø­%20Ø§ÙÙÙØªØ¨\Included%20Articles\Job%20satisfaction%20of%20staff%20working%20with%20individuals%20with%20intellectual%20disabilities%20in%20two%20different%20settings.pdf) | 2 | To identify the level of job satisfaction of healthcare staff working with individuals with intellectual disability in two settings in Bahrain. | 0 | Not mentioned | NO | 0 | job satisfaction questionnaire of the Balanced Score Card for Bahrain (BSC4B) and personal characteristics | 0 | several quantitative analyses | 2 | Found positive satisfaction with responsibility, management, appreciation, development, and salary and benefits. | 2 | Used 95% confidence interval | 0 | The survey was subjected to the bias and prejudices of the respondents, carried out in a short span of time, and could not be generalized to other organizations. | 0 | N/A | 0 | N/A | 6 |
| 38 | Psychosocial and occupational factors associated with low back pain among nurses in Saudi Arabia | [file:///C:/Users/mtok1/OneDrive/%D8%B3%D8%B7%D8%AD%20%D8%A7%D9%84%D9%85%D9%83%D8%AA%D8%A8/Included%20Articles/Psychosocial%20and%20occupational%20factors%20associated%20with%20low%20back%20pain%20among%20nurses%20in%20Saudi%20Arabia.pdf](file:///C:\Users\mtok1\OneDrive\Ø³Ø·Ø­%20Ø§ÙÙÙØªØ¨\Included%20Articles\Psychosocial%20and%20occupational%20factors%20associated%20with%20low%20back%20pain%20among%20nurses%20in%20Saudi%20Arabia.pdf) | 2 | To assessed psychosocial and occupational factors associated with LBP among nurses as a step to take preventive action to alter these modifiable risk factors for better health as a fundamental right for all. | 1 | convenience sample | YES | 2 | A self- reported questionnaire examined the prevalence of LBP and question on treatment, questionnaire related working conditions, demographic characteristics, and other question related to perceived stress at work, job satisfaction, plans to change jobs because of LBP, and negative life events during the past year. | 2 | Univariate and multivariate logistic regression analyses | 2 | Found negative satisfaction: lack of job satisfaction was independently and significantly associated with LBP. | 2 | Used 95% confidence interval | 0 | Lack of standardized measures for LBP, using cross sectional study design, and data were collected using a self-reported questionnaire which is inherently biased by the person's feeling at the time they filed it out. | 2 | Consistency | 0 | N/A | 13 |
| 39 | Assessment of job satisfaction, lifestyle behaviours, and occupational burnout symptoms during the COVID-19 pandemic among radiologic technologists in Saudi Arabia | [file:///C:/Users/mtok1/OneDrive/%D8%B3%D8%B7%D8%AD%20%D8%A7%D9%84%D9%85%D9%83%D8%AA%D8%A8/Included%20Articles/Assessment%20of%20job%20satisfaction,%20lifestyle%20behaviors,%20and%20occupational%20burnout%20symptoms%20during%20the%20COVID-19%20pandemic%20among%20radiologic%20technologists%20in%20Saudi%20Arabia.pdf](file:///C:\Users\mtok1\OneDrive\Ø³Ø·Ø­%20Ø§ÙÙÙØªØ¨\Included%20Articles\Assessment%20of%20job%20satisfaction,%20lifestyle%20behaviors,%20and%20occupational%20burnout%20symptoms%20during%20the%20COVID-19%20pandemic%20among%20radiologic%20technologists%20in%20Saudi%20Arabia.pdf) | 2 | To assess the extent of job satisfaction, burnout symptoms, lifestyle behaviours, and associated factors among RTs in Saudi Arabia. | 1 | convenience sample | YES | 2 | A self-administered questionnaire included four parts: socio-demographic information, answers to the Minnesota Satisfaction Questionnaire (MSQ), quality of their healthy lifestyle behaviours, and frequency of occupational burnout symptoms | 0 | descriptive and inferential statistics. | 2 | Found positive satisfaction with activity, independence, social status, moral values, security, social services, authority, creativity, and accomplishment. Negative satisfaction with compensation and advancement. | 2 | Used 95% confidence interval | 0 | small sample size, convenience sampling method, there was no baseline from before the pandemic against which to compare the interrelationships between job satisfaction, lifestyle behaviours, and burnout symptoms. | 0 | inconsistency | 2 | Important to policy makers | 11 |
| 40 | Relationship between interprofessional teamwork and nurses' intent to leave work: The mediating role of job satisfaction and burnout | [file:///C:/Users/mtok1/AppData/Local/Microsoft/Windows/INetCache/IE/1G79AB7T/Relationship_between_interprofessional_teamwork_and_nurses'_intent_to_leave_work_The_mediating_role_of_job_satisfaction_and_burnout[1].pdf](file:///C:\Users\mtok1\AppData\Local\Microsoft\Windows\INetCache\IE\1G79AB7T\Relationship_between_interprofessional_teamwork_and_nurses'_intent_to_leave_work_The_mediating_role_of_job_satisfaction_and_burnout%5b1%5d.pdf) | 2 | To examine the direct and indirect effects of interprofessional teamwork on nurses' intentions to leave their jobs via the intermediary roles of job satisfaction and burnout. | 1 | convenience sample | YES | 2 | A structured survey questionnaire for nurse demographics and work characteristics, interprofessional teamwork, job satisfaction, job burnout, and intention to leave | 1 | Logistic regression, descriptive statistic, sequential mediation model | 2 | Found positive satisfaction with interprofessional teamwork. Negative satisfaction with nurses' intention to leave and job burnout | 2 | Used 95% confidence interval | 0 | using cross sectional study. Although the relationship among the study variables was statically significant, other factors, such as workload stress may account for turnover intention. | 2 | Consistency | 2 | Important to policy makers and nurse manager | 14 |
| 41 | Job Satisfaction of Radiographers in Saudi Arabia | [file:///C:/Users/mtok1/OneDrive/%D8%B3%D8%B7%D8%AD%20%D8%A7%D9%84%D9%85%D9%83%D8%AA%D8%A8/Included%20Articles/Job%20satisfaction%20of%20Radiographers%20in%20Saudi%20Arabia.pdf](file:///C:\Users\mtok1\OneDrive\Ø³Ø·Ø­%20Ø§ÙÙÙØªØ¨\Included%20Articles\Job%20satisfaction%20of%20Radiographers%20in%20Saudi%20Arabia.pdf) | 2 | To evaluate job satisfaction among radiographers in Saudi Arabia and investigate factors contributing to their scores. | 1 | snowball sampling | YES | 2 | electronic questionnaire through multiple channels including specialized social media groups for radiologic technologists in Saudi Arabia on Twitter and Telegram. Also, Participants also were encouraged to share the survey with their peers as a method of snowball sampling. | 0 | descriptive and inferential statistics | 2 | Found positive satisfaction with nature of work, contingent rewards, and coworkers. Negative satisfaction with promotions, pay, operating conditions, and supervision | 2 | Used 95% confidence interval | 0 | It did not evaluate radiographers at other sectors besides hospitals, such as radiographer in the academia. The survey tool used for this study does not evaluate radiographer-specific job characteristics, such as equipment status and functioning. The possible effect of nonresponse bias in the survey results also must be acknowledged. The interventional radiology department was slightly underrepresented in the sample. | 0 | inconsistency | 2 | Important for governmental policymakers | 11 |
| 42 | The association between nationality and nurse job satisfaction in Saudi Arabian hospitals | [file:///C:/Users/mtok1/OneDrive/%D8%B3%D8%B7%D8%AD%20%D8%A7%D9%84%D9%85%D9%83%D8%AA%D8%A8/Included%20Articles/The%20association%20between%20nationality%20and%20nurse%20job%20satisfaction%20in%20Saudi%20Arabian%20hospitals.pdf](file:///C:\Users\mtok1\OneDrive\Ø³Ø·Ø­%20Ø§ÙÙÙØªØ¨\Included%20Articles\The%20association%20between%20nationality%20and%20nurse%20job%20satisfaction%20in%20Saudi%20Arabian%20hospitals.pdf) | 2 | To examine whether there is an association between nationality and nurse job satisfaction. | 0 | non-probability sampling | YES | 2 | Job satisfaction was measured using McCloskey/Mueller Satisfaction Scale. Additional questions for collecting key information around nationality, gender, age, years of nursing experience, the type of hospital and monthly salary. | 2 | Multiple linear regression analysis | 2 | Found positive association between age, years of experience, and monthly salary. expatriates were less satisfied than Saudi nurses about extrinsic rewards and family–work balance. Saudi nurses were less satisfied than expatriate nurses about their professional opportunities, praise and recognition, and co-worker relationships. | 2 | Used 95% confidence interval | 0 | 2 potential limitations may impact the study findings. First, sing non-probability sampling techniques. Second, related to the MMSS and its reliability. | 0 | inconsistency | 2 | Important for policy makers | 12 |
| 43 | Linking relational coordination to nurses’ job satisfaction, affective commitment and turnover intention in Saudi Arabia | [file:///C:/Users/mtok1/OneDrive/%D8%B3%D8%B7%D8%AD%20%D8%A7%D9%84%D9%85%D9%83%D8%AA%D8%A8/Included%20Articles/R.%20Falatah%20-%20Linking%20relational%20coordination%20to%20nurses%E2%80%99%20job%20satisfaction,%20affective%20commitment%20and%20turnover%20intention%20in%20Saudi%20Arabia.pdf](file:///C:\Users\mtok1\OneDrive\Ø³Ø·Ø­%20Ø§ÙÙÙØªØ¨\Included%20Articles\R.%20Falatah%20-%20Linking%20relational%20coordination%20to%20nursesâ%20job%20satisfaction,%20affective%20commitment%20and%20turnover%20intention%20in%20Saudi%20Arabia.pdf) | 2 | To examine the association between relational coordination, job satisfaction, affective commitment and turnover intention. | 1 | convenience sample | YES | 2 | A self reported survey included relational coordination, job satisfaction, affective commitment, turnover intention, and demographics | 0 | Data were analysed using SPSS and the hypothesis were tested using Hayes' (2013) PROCESS macro for mediation. | 2 | Found positive satisfaction with relational coordination (strong) | 2 | Used 95% confidence interval | 0 | Using cross sectional which limits the extent to which inferences regarding causality can be made, study assessed only nurses' perceptions of relational coordination, convenience sample was used which present the possibility of self-selection bias and may not presenting the nursing population in general, the data were obtained through self-reported surveys, the potential for common method bias cannot be entirely ruled out, and sample was predominately female. | 2 | Consistency | 0 | N/A | 11 |
| 44 | Linking nurses' job security to job satisfaction and turnover intention during reform and privatization: A cross-sectional survey | [file:///C:/Users/mtok1/AppData/Local/Microsoft/Windows/INetCache/IE/AU42TPSJ/R._Falatah_-_Linking_nurses%E2%80%99_job_security_to_job_satisfaction_and_turnover_intention_during_reform_and_privatization__A_cross%E2%80%90sectional_survey[1].pdf](file:///C:\Users\mtok1\AppData\Local\Microsoft\Windows\INetCache\IE\AU42TPSJ\R._Falatah_-_Linking_nursesâ_job_security_to_job_satisfaction_and_turnover_intention_during_reform_and_privatization__A_crossâsectional_survey%5b1%5d.pdf) | 2 | To examine the link between job security, job satisfaction and turnover intention during the reform and privatization of a health care system. | 1 | convenience sample | YES | 2 | A self reported survey included 4 parts: demographic data, job satisfaction, turnover intention, and job security | 2 | SPSS, univariate, bivariate, multivariate analysis, and multiple regression models. | 2 | Found positive satisfaction with job security (moderate). | 2 | Used 95% confidence interval | 0 | using cross sectional studies that limit its inferences of causality, convenient sampling might have introduced self-selection bias and compromised the generalizability of the study findings, using self-reported survey, although the participants were anonymous, self-reporting might increase reporting error due to social desirability. | 2 | Consistency | 2 | Important for nurse manager and leaders | 15 |
| 45 | A comparative study on job satisfaction between registered nurses and other qualified healthcare professionals | [file:///C:/Users/mtok1/OneDrive/%D8%B3%D8%B7%D8%AD%20%D8%A7%D9%84%D9%85%D9%83%D8%AA%D8%A8/Included%20Articles/M.%20Parveen%20-%20A%20comparative%20study%20on%20job%20satisfaction%20between%20registered%20nurses%20and%20other%20qualified%20healthcare%20professionals.pdf](file:///C:\Users\mtok1\OneDrive\Ø³Ø·Ø­%20Ø§ÙÙÙØªØ¨\Included%20Articles\M.%20Parveen%20-%20A%20comparative%20study%20on%20job%20satisfaction%20between%20registered%20nurses%20and%20other%20qualified%20healthcare%20professionals.pdf) | 2 | To investigate the level of job satisfaction among RNs and QHPs and to explore the relationship of their nationalities and types of healthcare setting with job satisfaction. | 1 | conveniently distributed | YES | 2 | A self-administered questionnaire included 2 parts: job satisfaction and demographic | 2 | Multivariate analysis of variance | 2 | Found negative satisfaction with personal growth and professional support. RNs were not satisfied with their salary package. | 2 | Used 95% confidence interval | 0 | N/A | 0 | inconsistency | 2 | Important for managers and leaders | 13 |
| 46 | Factors that affect the job satisfaction of Saudi Arabian nurses | [file:///C:/Users/mtok1/OneDrive/%D8%B3%D8%B7%D8%AD%20%D8%A7%D9%84%D9%85%D9%83%D8%AA%D8%A8/Included%20Articles/J%20Nursing%20Management%20-%202015%20-%20Alotaibi%20-%20Factors%20that%20affect%20the%20job%20satisfaction%20of%20Saudi%20Arabian%20nurses.pdf](file:///C:\Users\mtok1\OneDrive\Ø³Ø·Ø­%20Ø§ÙÙÙØªØ¨\Included%20Articles\J%20Nursing%20Management%20-%202015%20-%20Alotaibi%20-%20Factors%20that%20affect%20the%20job%20satisfaction%20of%20Saudi%20Arabian%20nurses.pdf) | 2 | To determine the levels of job satisfaction and to collect information about the factors that affect job satisfaction of Saudi nurses. | 2 | random sample | YES | 2 | an existing validated survey (Muller & McCloskey) was used to collect the predominantly quantitative data. First part related to demographic questions. Second part related to satisfaction. Additional questions were added related to job satisfaction relevant to the Saudi culture | 0 | content analysis | 2 | Found positive satisfaction with influence of religion. Negative satisfaction with educational opportunity, work environment in relation to heavy workload and long hours, and favouritism | 2 | Used 95% confidence interval | 0 | N/A | 2 | Consistency | 2 | Important for nursing management | 14 |
| 47 | Physician satisfaction with electronic medical records in a major Saudi Government hospital | [file:///C:/Users/mtok1/OneDrive/%D8%B3%D8%B7%D8%AD%20%D8%A7%D9%84%D9%85%D9%83%D8%AA%D8%A8/Included%20Articles/Physician%20satisfaction%20with%20electronic%20medical%20records%20in%20a%20major%20Saudi%20Government%20hospital%20_%20Elsevier%20Enhanced%20Reader.pdf](file:///C:\Users\mtok1\OneDrive\Ø³Ø·Ø­%20Ø§ÙÙÙØªØ¨\Included%20Articles\Physician%20satisfaction%20with%20electronic%20medical%20records%20in%20a%20major%20Saudi%20Government%20hospital%20_%20Elsevier%20Enhanced%20Reader.pdf) | 2 | To measure physician satisfaction with a recently introduced electronic medical record (EMR) system and to determine which of the individual attributes of EMR were related to physician satisfaction. | 0 | Not mentioned | YES | 2 | a self-administered survey based on the Delone and Mclean model. Including sociodemographic characteristics and satisfaction with the system. | 2 | Pearson's correlation coefficient and multiple linear regression analysis | 2 | Found negative satisfaction with the EMRs system | 2 | Used 95% confidence interval | 0 | It was performed in one Governmental hospital with one EMR system and only 52% of the eligible physicians participated in the survey. | 2 | Consistency | 2 | Important for hospital management and stakeholders | 14 |
| 48 | Prevalence of stress, burnout, and job satisfaction among mental healthcare professionals in Jeddah, Saudi Arabia | [file:///C:/Users/mtok1/OneDrive/%D8%B3%D8%B7%D8%AD%20%D8%A7%D9%84%D9%85%D9%83%D8%AA%D8%A8/Included%20Articles/Prevalence%20of%20stress,%20burnout,%20and%20job%20satisfaction%20among%20mental%20healthcare%20professionals%20in%20Jeddah,%20Saudi%20Arabia.pdf](file:///C:\Users\mtok1\OneDrive\Ø³Ø·Ø­%20Ø§ÙÙÙØªØ¨\Included%20Articles\Prevalence%20of%20stress,%20burnout,%20and%20job%20satisfaction%20among%20mental%20healthcare%20professionals%20in%20Jeddah,%20Saudi%20Arabia.pdf) | 2 | To measure the prevalence of stress, burnout, and to assess job satisfaction among different mental healthcare professionals in Jeddah City, Saudi Arabia. | 2 | All psychiatrists, psychiatric residents, psychologists, social workers, and psychiatric nurses. Random sample | YES | 2 | A self-administered questionnaire including Sociodemographic characteristics and levels of stress, burnout, and job satisfaction were assessed using the Perceived Stress Scale-14 (PSS-14), Maslach Burnout Inventory (MBI), and Job Satisfaction Scale (JSS). | 0 | Descriptive statistics | 2 | Found positive satisfaction with supervision, nature of work, monthly income above 20000. Negative satisfaction with operating conditions | 2 | Used 95% confidence interval | 0 | Excluded non-English speakers because the JSS had no validated Arabic version, the study was conducted in one city and in cross-sectional design which limits its generalizability to all settings in S.A., and some professions were underrepresented in our sample | 2 | Consistency | 0 | N/A | 12 |
| 49 | The impact of administrative empowerment on the job satisfaction of employees | [file:///C:/Users/mtok1/OneDrive/%D8%B3%D8%B7%D8%AD%20%D8%A7%D9%84%D9%85%D9%83%D8%AA%D8%A8/Included%20Articles/S.%20A.%20Al%20Naggar%20-%20The%20impact%20of%20administrative%20empowerment%20on%20the%20job%20satisfaction%20of%20employees.pdf](file:///C:\Users\mtok1\OneDrive\Ø³Ø·Ø­%20Ø§ÙÙÙØªØ¨\Included%20Articles\S.%20A.%20Al%20Naggar%20-%20The%20impact%20of%20administrative%20empowerment%20on%20the%20job%20satisfaction%20of%20employees.pdf) | 1 | To investigate the impact of administrative empowerment on workers of the Health Directorate in the Northern Border Region in the Kingdom of Saudi Arabia. | 2 | stratified random sample | NO | 0 | A self-administered questionnaire included 33 phrases | 0 | Descriptive statistics | 0 | None | 2 | Used 95% confidence interval | 0 | we conclude that the first main hypothesis and the sub-hypotheses are rejected, which states that “there is no significant effect of the dimensions….... etc | 2 | Consistency | 1 | Important for management | 8 |
| 50 | Physicians' job satisfaction, ethics and burnout in Makkah, Saudi Arabia | [file:///C:/Users/mtok1/OneDrive/%D8%B3%D8%B7%D8%AD%20%D8%A7%D9%84%D9%85%D9%83%D8%AA%D8%A8/Included%20Articles/Physicians%E2%80%99%20job%20satisfaction,%20ethics%20and%20burnout%20in%20Makkah,%20Saudi%20Arabia.pdf](file:///C:\Users\mtok1\OneDrive\Ø³Ø·Ø­%20Ø§ÙÙÙØªØ¨\Included%20Articles\Physiciansâ%20job%20satisfaction,%20ethics%20and%20burnout%20in%20Makkah,%20Saudi%20Arabia.pdf) | 2 | To determine the rates of physicians' job satisfaction, commitment to professional morals, and burnout at Makkah, Saudi Arabia's general hospitals. | 2 |  | YES | 2 | A self-administered questionnaire included sociodemographic information, detailed questions about burnout, satisfaction and ethics | 1 | bivariate statistical analysis | 0 | None | 2 | Used 95% confidence interval | 0 | small sample size, possibility of being underpowered to identify smaller average differences in analysis as well as large percentage of male participants. Based on external validity; this study was conducted in S.A. where medical practice might be slightly different from other countries. Cultural norms vary between populations. | 2 | Consistency | 0 | N/A | 11 |
| 51 | Burnout and job satisfaction among psychiatrists in the Mental Health Service, Hamad Medical Corporation, Qatar | [file:///C:/Users/mtok1/OneDrive/%D8%B3%D8%B7%D8%AD%20%D8%A7%D9%84%D9%85%D9%83%D8%AA%D8%A8/Included%20Articles/Burnout%20and%20job%20satisfaction%20among%20psychiatrists%20in%20the%20Mental%20Health%20Service,%20Hamad%20Medical%20Corporation,%20Qatar.pdf](file:///C:\Users\mtok1\OneDrive\Ø³Ø·Ø­%20Ø§ÙÙÙØªØ¨\Included%20Articles\Burnout%20and%20job%20satisfaction%20among%20psychiatrists%20in%20the%20Mental%20Health%20Service,%20Hamad%20Medical%20Corporation,%20Qatar.pdf) | 2 | To determine the prevalence of burnout and level of job satisfaction among psychiatrists working in the Mental Health Service, Hamad Medical Corporation, Qatar, and examine correlations among socio-demographic variables, burnout, and job satisfaction. | 2 | random sample | YES | 2 | self reporting questionnaire, socio-demographic questionnaire, the Maslach Burnout Inventory (MBI), and Job Descriptive Index (JDI) | 2 | Descriptive statistics, T-test or one-way analysis of variance, Chi-square tests, Correlation analyses and linear regression, logistic regression, Pearson's correlation, and multiple regression analysis | 2 | Found positive satisfaction with job in general, work on the present job, co-workers, pay, and supervision. Negative satisfaction with opportunities for promotion | 2 | Used 95% confidence interval | 0 | cross sectional study could only highlight observational and possible associations but cannot demonstrate causality, the survey relied on self-reporting, JDI which was based on business sector, may not fully capsulate the mental health sector's issues. JDI does not appear to capture organizational factors, such as management or leadership factors. unable to compare the respondent's socio-demographic data with non-respondents. | 2 | Consistency | 0 | N/A | 14 |
| 52 | Nurses’ job satisfaction during the mass gathering of the Hajj 2018 in Saudi Arabia | [file:///C:/Users/mtok1/OneDrive/%D8%B3%D8%B7%D8%AD%20%D8%A7%D9%84%D9%85%D9%83%D8%AA%D8%A8/Included%20Articles/Nurses%E2%80%99%20job%20satisfaction%20during%20the%20mass%20gathering%20of%20the%20Hajj%202018%20in%20Saudi%20Arabia.pdf](file:///C:\Users\mtok1\OneDrive\Ø³Ø·Ø­%20Ø§ÙÙÙØªØ¨\Included%20Articles\Nursesâ%20job%20satisfaction%20during%20the%20mass%20gathering%20of%20the%20Hajj%202018%20in%20Saudi%20Arabia.pdf) | 2 | To explore nurses' job satisfaction when providing health care during the pilgrimage of Hajj in Saudi Arabia | 1 | convenience sample | YES | 2 | structured self-administered Nursing Job Satisfaction Questionnaire comprised 24 item scale that measured 6 subscales which represented 6 domains: supervision, nature of work, retention, communication, workload, and co-workers | 2 | descriptive and inferential statistics, and multiple linear regression analysis | 2 | Found positive satisfaction with 6 domains (nature of work, retention, communication, workload, co-workers, supervision). Negative satisfaction with ‘multiple policies and procedures that were perceived as complicating nursing work’, ‘incompetence of other people they work with’ and ‘too much burden at work’. | 2 | Used 95% confidence interval | 2 | small sample size, the sample was recruited from limited number of healthcare facilities during a specific period, which means results should be generalized to other regions of Saudi Arabia and beyond with caution. | 2 | Consistency | 2 | Important for MOH and policymakers | 17 |
| 53 | Job satisfaction amongst surgical healthcare professionals during Hajj and Non-Hajj periods: An analytical multi-center cross-sectional study in the holy city of Makkah, Saudi Arabia | [file:///C:/Users/mtok1/OneDrive/%D8%B3%D8%B7%D8%AD%20%D8%A7%D9%84%D9%85%D9%83%D8%AA%D8%A8/Included%20Articles/Job%20satisfaction%20amongst%20surgical%20healthcare%20professionals%20during%20Hajj%20and%20Non-Hajj%20periods%20An%20analytical%20multi-center%20cross-sectional%20study%20in%20the%20holy%20city%20of%20Makkah,%20Saudi%20Arabia.pdf](file:///C:\Users\mtok1\OneDrive\Ø³Ø·Ø­%20Ø§ÙÙÙØªØ¨\Included%20Articles\Job%20satisfaction%20amongst%20surgical%20healthcare%20professionals%20during%20Hajj%20and%20Non-Hajj%20periods%20An%20analytical%20multi-center%20cross-sectional%20study%20in%20the%20holy%20city%20of%20Makkah,%20Saudi%20Arabia.pdf) | 2 | To assess the job satisfaction of surgical healthcare professionals working during Hajj, and to determine the potential predictors of overall job satisfaction in Hajj and non-Hajj periods. | 2 | All surgeons who were employed during Hajj were interviewed regarding their job satisfaction in-Hajj versus non-Hajj periods (random sample) | YES | 2 | Questionnaire had 2 parts: First part explored demographic data, job designation and the healthcare centre. Second part explored the core of the study and consisted of a short version of Warr-Cook-Wall (WCW) job satisfaction scale | 2 | Descriptive analysis, stepwise multiple linear regression, and regression analysis | 2 | Found positive satisfaction with amount of variety in work during hajj period. Negative satisfaction with physical working condition, hours of work, and attention paid to suggestions during hajj period | 2 | Used 95% confidence interval | 0 | N/A | 2 | Consistency | 0 | N/A | 14 |
| 54 | Job Satisfaction among Primary Health Care Workers in Buraidah, Qassim, Saudi Arabia | [file:///C:/Users/mtok1/OneDrive/%D8%B3%D8%B7%D8%AD%20%D8%A7%D9%84%D9%85%D9%83%D8%AA%D8%A8/Included%20Articles/E.%20Aljumail%20-%20Job%20Satisfaction%20among%20Primary%20Health%20Care%20Workers%20in%20Buraidah,%20Qassim,%20Saudi%20Arabia.pdf](file:///C:\Users\mtok1\OneDrive\Ø³Ø·Ø­%20Ø§ÙÙÙØªØ¨\Included%20Articles\E.%20Aljumail%20-%20Job%20Satisfaction%20among%20Primary%20Health%20Care%20Workers%20in%20Buraidah,%20Qassim,%20Saudi%20Arabia.pdf) | 2 | To assess the job satisfaction among primary health care (PHC) workers in Buraidah, Saudi Arabia. | 2 | random sample | YES | 2 | self-administered questionnaire that consists of 3 parts: informed consent and explanation of the study's purpose and information about the researcher, demographic data, and job satisfaction survey (JSS). | 0 | Descriptive statistics, Chi square and ANOVA | 2 | Found positive satisfaction with nature of work, co-workers and supervision. Negative satisfaction with operating conditions. | 2 | Used 95% confidence interval | 0 | the study was done during the COVID-19 pandemic, and we faced difficulties in obtaining data due to change in work conditions. Furthermore, the pandemic may have altered and affected the result of job satisfaction. Finally, the study was done in single city, Buraidah, due time and resource limitations which may affect the generalizability of our results to the whole region. Nevertheless, our results are important as we conducted the study in a regional capital while job conditions and social circumstances may be poor in smaller cities and rural areas, and we may expect even lower satisfaction in those areas | 2 | Consistency | 2 | Important for policymakers | 14 |
| 55 | Factors affecting job satisfaction among medical laboratory technologists in University Hospital, Oman: An exploratory study | [file:///C:/Users/mtok1/OneDrive/%D8%B3%D8%B7%D8%AD%20%D8%A7%D9%84%D9%85%D9%83%D8%AA%D8%A8/Included%20Articles/S.%20Alrawahi%20-%20Factors%20affecting%20job%20satisfaction%20among%20medical%20laboratory%20technologists%20in%20University%20Hospital,%20Oman_%20An%20exploratory%20study.pdf](file:///C:\Users\mtok1\OneDrive\Ø³Ø·Ø­%20Ø§ÙÙÙØªØ¨\Included%20Articles\S.%20Alrawahi%20-%20Factors%20affecting%20job%20satisfaction%20among%20medical%20laboratory%20technologists%20in%20University%20Hospital,%20Oman_%20An%20exploratory%20study.pdf) | 1 | This study explores the factors that impact upon job satisfaction among medical laboratory technologists in University Hospital, Oman. | 2 | random sample | YES | 1 | Exploratory study, general question: can you please describe your situation at work in this hospital? Including socio demographic variables | 0 | An exploratory study | 2 | Found positive satisfaction with salary, the co-worker relationship, and job security for non-Omanis. Negative satisfaction with workload, promotion, health and safety in the laboratory, relationship with the leaders, professional status (recognition and appreciation), and hospital policies such as appraisal. | 0 | An exploratory study | 0 | The research was performed in one Hospital in Oman, and the insight won have cannot necessarily be generalised. | 2 | Consistency | 0 | N/A | 8 |
| 56 | Perception, attitude, and satisfaction of paediatric physicians and nurses towards clinical practice guidelines at a university teaching hospital | [file:///C:/Users/mtok1/OneDrive/%D8%B3%D8%B7%D8%AD%20%D8%A7%D9%84%D9%85%D9%83%D8%AA%D8%A8/Included%20Articles/Perception,%20attitude,%20and%20satisfaction%20of%20paediatric%20physicians%20and%20nurses%20towards%20clinical%20practice%20guidelines%20at%20a%20university%20teaching%20hospital.pdf](file:///C:\Users\mtok1\OneDrive\Ø³Ø·Ø­%20Ø§ÙÙÙØªØ¨\Included%20Articles\Perception,%20attitude,%20and%20satisfaction%20of%20paediatric%20physicians%20and%20nurses%20towards%20clinical%20practice%20guidelines%20at%20a%20university%20teaching%20hospital.pdf) | 2 | To explore perception, attitude, and satisfaction of paediatric clinicians, trainees, and nurses at King Khalid University Hospital towards clinical practice guidelines (CPGs) including the locally adapted diabetic ketoacidosis CPG (DKA-CPG). | 1 | convenience sample | YES | 2 | A self-administered questionnaire included 3 sections: demographic data, the perception and attitude towards CPGs, and the perception and attitude towards the DKA-CPG. | 0 | SPSS, Kolmogorov-Smirnov test, and inferential statistics, Mann-Whitney U test | 2 | Found positive satisfaction with clinical practice guidelines | 2 | Used 95% confidence interval | 0 | it is not possible to generalize the findings to other hospitals at other healthcare sectors as the logistics, and the infrastructures vary between different Institutions. We conducted the survey after period of implementation of the DKA- CPG and that may have influenced the attitude and perception of the participants | 2 | Consistency | 0 | N/A | 11 |
| 57 | Job satisfaction among Saudi healthcare workers and its impact on the quality of health services | [file:///C:/Users/mtok1/OneDrive/%D8%B3%D8%B7%D8%AD%20%D8%A7%D9%84%D9%85%D9%83%D8%AA%D8%A8/Included%20Articles/Job%20satisfaction%20among%20Saudi%20healthcare%20workers%20and%20its%20impact%20on%20the%20quality%20of%20health%20services.pdf](file:///C:\Users\mtok1\OneDrive\Ø³Ø·Ø­%20Ø§ÙÙÙØªØ¨\Included%20Articles\Job%20satisfaction%20among%20Saudi%20healthcare%20workers%20and%20its%20impact%20on%20the%20quality%20of%20health%20services.pdf) | 2 | To assess the impact of job satisfaction on the quality of health services | 2 | random sample | YES | 2 | A self-designed questionnaire included demographic data, factors that affected the job satisfaction of the participants and the factors that impacted the quality of the provided care in the hospitals | 0 | Descriptive correlation analysis | 2 | Found positive satisfaction with working hours, public sector, working experience length, quality of care provided. Negative satisfaction with stress management, work environment, pay benefit, patient satisfaction | 2 | Used 95% confidence interval | 0 | N/A | 2 | Consistency | 2 | Important for organizations' management | 14 |
| 58 | Predictors and level of job satisfaction among the dental workforce in national guard health affairs | [file:///C:/Users/mtok1/OneDrive/%D8%B3%D8%B7%D8%AD%20%D8%A7%D9%84%D9%85%D9%83%D8%AA%D8%A8/Included%20Articles/Predictors%20and%20level%20of%20job%20satisfaction%20among%20the%20dental%20workforce%20in%20national%20guard%20health%20affairs.pdf](file:///C:\Users\mtok1\OneDrive\Ø³Ø·Ø­%20Ø§ÙÙÙØªØ¨\Included%20Articles\Predictors%20and%20level%20of%20job%20satisfaction%20among%20the%20dental%20workforce%20in%20national%20guard%20health%20affairs.pdf) | 2 | To assess the level of job satisfaction among the dental workforce in the National Guard Health Affairs (NGHA) in Saudi Arabia and to explore any predictors that have impact on the level of satisfaction. | 2 | A cluster random sampling | YES | 2 | A self-reported questionnaire that was previously published. It comprised: scoiodemographic, satisfaction statement of29 questions related to 4 domains: professional and personal life, income and job security, quality of service, and prestige and self-perception | 0 | one-way analysis of variance | 2 | Found positive satisfaction with the quality of service they provided, prestige and self-perception, professional and personal life, and income and job security | 2 | Used 95% confidence interval | 1 | generalizability of the results of this survey to all dental health-care workers in the NGHA should be taken with precaution. In addition, generalizability of the results to all Saudi dentists and dental auxiliaries should be considered with uncertainty. It is possible that dentists and dental auxiliaries from other geographical areas may respond differently to the survey instrument used in this study. | 2 | Consistency | 0 | N/A | 13 |
| 59 | Job satisfaction and organizational commitment of paramedics - A case study of Saudi Arabia | [file:///C:/Users/mtok1/OneDrive/%D8%B3%D8%B7%D8%AD%20%D8%A7%D9%84%D9%85%D9%83%D8%AA%D8%A8/Included%20Articles/Job%20satisfaction%20and%20organizational%20commitment%20of%20paramedics%20-%20A%20case%20study%20of%20Saudi%20Arabia.pdf](file:///C:\Users\mtok1\OneDrive\Ø³Ø·Ø­%20Ø§ÙÙÙØªØ¨\Included%20Articles\Job%20satisfaction%20and%20organizational%20commitment%20of%20paramedics%20-%20A%20case%20study%20of%20Saudi%20Arabia.pdf) | 2 | To look further into the association among the dimensions of job satisfaction (JS) and organizational commitment (OC) vis-a-vis paramedical staff in Saudi Arabia. | 1 | convenience sampling | YES | 2 | Organizational Commitment Questionnaire (OCQ) and Job Descriptive Index (JDI) including 3 demographic variables | 2 | Multiple correlation and regression techniques were employed to test the hypotheses, descriptive and inferential statistical tools were applied | 2 | Found positive satisfaction with with their jobs, pay, supervision, promotion, and co-workers, and organizational commitment. Negative satisfaction with work itself | 2 | Used 95% confidence interval | 0 | The current study is restricted to only one city and private hospitals, most of the paramedics are contingent workers in a sense that almost all expatriates are hired on contract basis | 2 | Consistency | 2 | Important for hospital management and administrators | 15 |
| 60 | The application of Herzberg's two-factor theory of motivation to job satisfaction in clinical laboratories in Omani hospitals | [file:///C:/Users/mtok1/OneDrive/%D8%B3%D8%B7%D8%AD%20%D8%A7%D9%84%D9%85%D9%83%D8%AA%D8%A8/Included%20Articles/The%20application%20of%20Herzberg's%20two-factor%20theory%20of%20motivation%20to%20job%20satisfaction%20in%20clinical%20laboratories%20in%20Omani%20hospitals.pdf](file:///C:\Users\mtok1\OneDrive\Ø³Ø·Ø­%20Ø§ÙÙÙØªØ¨\Included%20Articles\The%20application%20of%20Herzberg's%20two-factor%20theory%20of%20motivation%20to%20job%20satisfaction%20in%20clinical%20laboratories%20in%20Omani%20hospitals.pdf) | 2 | To explore what motivational elements are associated with job satisfaction among medical laboratory professionals (MLPs) in Oman. | 2 | (FGD) | YES | 2 | (FGDs) were used for data collection including sociodemographic variables | 0 | Directed content analysis, and frequencies of statement | 2 | Found positive satisfaction with relationships with co-workers, relationship with leaders, and professional development. Negative satisfaction with health and safety, heavy workload, salary, promotion, recognition and organizational policies | 2 | Used 95% confidence interval | 0 | The findings cannot be generalized to the whole populations of MLPs, although the number of participants was large, and the participants were well representative of the laboratory staff of the three hospitals involved | 2 | Consistency | 2 | Important for hospital managers and administrations | 14 |
| 61 | Factors affecting job satisfaction and their correlation with educational standards among dental assistants | [file:///C:/Users/mtok1/OneDrive/%D8%B3%D8%B7%D8%AD%20%D8%A7%D9%84%D9%85%D9%83%D8%AA%D8%A8/Included%20Articles/Al-jazairy-2014-Factors-affecting-job-satisfaction-.pdf](file:///C:\Users\mtok1\OneDrive\Ø³Ø·Ø­%20Ø§ÙÙÙØªØ¨\Included%20Articles\Al-jazairy-2014-Factors-affecting-job-satisfaction-.pdf) | 2 | To assess the level and distribution of job satisfaction among dental assistant working in various private and public hospitals, clinics, polyclinics and dental schools in the kingdom of Saudi Arabia | 2 | random sample | YES | 2 | A self-administered questionnaire including: Dental Assistant Satisfaction Survey (DASS), demographic details, general prospects of the profession indicating overall job satisfaction, and work environment factors. | 2 | Multinomial logistic regression and covariance | 2 | Found positive satisfaction with quality of service, general prospect of the profession | 2 | used 95% confidence interval | 1 | cross-sectional design and non-respondent bias, It is possible that dental assistants from other geographical areas may respond differently to the survey instrument used in this study, the reliability of the scale used to measure the quality of service, the perceptions of income, prestige and self-respect were relatively low, and because the study population only included dental assistants from Riyadh, Saudi Arabia, the findings obtained from these self-reported data can only be generalized within this population | 2 | Consistency | 2 | Important for policy makers and healthcare system | 17 |
| 62 | The relationship between practice environment, job satisfaction and intention to leave in critical care nurses | [file:///C:/Users/mtok1/OneDrive/%D8%B3%D8%B7%D8%AD%20%D8%A7%D9%84%D9%85%D9%83%D8%AA%D8%A8/Included%20Articles/The%20relationship%20between%20practice%20environment,%20job%20satisfaction%20and%20intention%20to%20leave%20in%20critical%20care%20nurses.pdf](file:///C:\Users\mtok1\OneDrive\Ø³Ø·Ø­%20Ø§ÙÙÙØªØ¨\Included%20Articles\The%20relationship%20between%20practice%20environment,%20job%20satisfaction%20and%20intention%20to%20leave%20in%20critical%20care%20nurses.pdf) | 2 | To examine the relationship between perception of nursing practice environment (NPE), job satisfaction and intention to leave (ITL) among critical care nurses working in the state of Hail in KSA. | 2 | random sample | YES | 2 | A self-report questionnaire included: demographic data, the Practice Environment Scale of the Nursing Work Index (PES-NWI), nursing Workplace Satisfaction Questionnaire (NWSQ), and turnover Intention Scale (TIS-6) | 2 | Descriptive statistics, spearman's rho bivariate correlations, and multiple linear regression | 2 | Found positive satisfaction with nurses practice environment. Negative satisfaction with intention to leave and working experience | 2 | Used 95% confidence interval | 0 | Using self report questionnaire that inherently carries the risk of response-bias, the single site setting limits the generalizability of the results, and subgroup analysis was not conducted. | 2 | Consistency | 2 | Important for critical care and hospital leaders | 16 |
| 63 | Job satisfaction, work commitment and intention to leave among pharmacists: a cross-sectional study | [file:///C:/Users/mtok1/OneDrive/%D8%B3%D8%B7%D8%AD%20%D8%A7%D9%84%D9%85%D9%83%D8%AA%D8%A8/Included%20Articles/Job%20satisfaction,%20work%20commitment%20and%20intention%20to%20leave%20among%20pharmacists%20a%20cross-sectional%20study.pdf](file:///C:\Users\mtok1\OneDrive\Ø³Ø·Ø­%20Ø§ÙÙÙØªØ¨\Included%20Articles\Job%20satisfaction,%20work%20commitment%20and%20intention%20to%20leave%20among%20pharmacists%20a%20cross-sectional%20study.pdf) | 2 | To assess job satisfaction, work commitment and intention to leave among pharmacists working in different healthcare settings in Saudi Arabia. | 2 | random sample | YES | 2 | A self-administered questionnaire included 8 sections: sociodemographic characteristics, current job features, job satisfaction and work commitment, overall satisfaction with their current job, intention to leave their current job, overall patient safety at their workplace and opinions on how to improve job satisfaction and work commitment among pharmacists working in Saudi Arabia | 2 | Multiple logistic regression analysis, descriptive ana analytical statistics | 2 | Found positive satisfaction with their current job. Negative satisfaction with intention to leave | 2 | Used 95% confidence interval | 1 | Because of the study design used, there is a chance that the association identified may have been misinterpreted, limited duration and timing of data collection prevented us from getting more responses, it covers only pharmacists in Riyadh, our results were based on the self-reported perceptions of the study's participants and are therefore subject to bias | 2 | Consistency | 2 | Important for policymakers and health planners | 17 |
| 64 | The Relationship between Job Stress and Job Satisfaction among Saudi Nurses: A Cross-Sectional Study | [file:///C:/Users/mtok1/OneDrive/%D8%B3%D8%B7%D8%AD%20%D8%A7%D9%84%D9%85%D9%83%D8%AA%D8%A8/Included%20Articles/O.%20G.%20Baker%20-%20The%20Relationship%20between%20Job%20Stress%20and%20Job%20Satisfaction%20among%20Saudi%20Nurses_%20A%20Cross-Sectional%20Study.pdf](file:///C:\Users\mtok1\OneDrive\Ø³Ø·Ø­%20Ø§ÙÙÙØªØ¨\Included%20Articles\O.%20G.%20Baker%20-%20The%20Relationship%20between%20Job%20Stress%20and%20Job%20Satisfaction%20among%20Saudi%20Nurses_%20A%20Cross-Sectional%20Study.pdf) | 2 | To examine the relationship between work-related stress and job satisfaction among Saudi nurses working at a public hospital. | 1 | convenience sampling | YES | 2 | Expanded Nursing Stress Scale (ENSS) and Job Satisfaction Scale (JSS), demographic characteristics | 0 | Descriptive statistics, SPSS, Pearson's correlation test | 2 | Found positive satisfaction with nature of work. Negative satisfaction with the benefits provided to nurses | 2 | Used 95% confidence interval | 0 | The research involves certain limitation in the form time and financial resources, cross-sectional study design limits the study results as the cause of stress cannot be determined, it conducted on a single institute, which limits its result generalization, and small sample size. | 2 | inconsistency | 2 | Important for nurses' managers | 13 |
| 65 | JOB SATISFACTION AND ORGANIZATIONAL COMMITMENT OF DOCTORS: A CASE STUDY OF SAUDI ARABIA | [file:///C:/Users/mtok1/OneDrive/%D8%B3%D8%B7%D8%AD%20%D8%A7%D9%84%D9%85%D9%83%D8%AA%D8%A8/Included%20Articles/JOB%20SATISFACTION%20AND%20ORGANIZATIONAL%20COMMITMENT%20OF%20DOCTORS%20A%20CASE%20STUDY%20OF%20SAUDI%20ARABIA.pdf](file:///C:\Users\mtok1\OneDrive\Ø³Ø·Ø­%20Ø§ÙÙÙØªØ¨\Included%20Articles\JOB%20SATISFACTION%20AND%20ORGANIZATIONAL%20COMMITMENT%20OF%20DOCTORS%20A%20CASE%20STUDY%20OF%20SAUDI%20ARABIA.pdf) | 2 | The present study focuses on the job satisfaction (JS) and organizational commitment (OC) vis-a-vis doctors in Saudi Arabia. | 1 | Convenience sampling | YES | 2 | A self-administered questionnaire included: organizational commitment questionnaire (OCQ), job descriptive index (JDI), and sociodemographic characteristics | 0 | Descriptive and inferential statistical tools using correlation and regression | 2 | Found positive satisfaction with organizational commitment, work, pay, promotion, supervision, co-workers, and age | 2 | Used 95% confidence interval | 0 | The current study is restricted to only six cities, in Saudi Arabia, and only public hospitals are explored so the generalizability of the current study may to some extent is constrained, Gender disparity is witnessed as only 39% of the respondents were female doctors, as the doctors are contingent workers in a sense that almost all expatriate are hired on contract basis as such their organizational commitment raises a big question mark, and further research on varied frame works with the same study variables can be carried out using some sociodemographic variables, turnover intention and different leadership traits with moderation and mediation effects before a generalization is made | 2 | Consistency somewhat | 2 | Important for healthcare organization and healthcare managers | 13 |
| 66 | Quality of work life: The determinants of job satisfaction and job retention among RNs and OHPs | [file:///C:/Users/mtok1/OneDrive/%D8%B3%D8%B7%D8%AD%20%D8%A7%D9%84%D9%85%D9%83%D8%AA%D8%A8/Included%20Articles/Quality%20of%20work%20life%20The%20determinants%20of%20job%20satisfaction%20and%20job%20retention%20among%20RNs%20and%20OHPs.pdf](file:///C:\Users\mtok1\OneDrive\Ø³Ø·Ø­%20Ø§ÙÙÙØªØ¨\Included%20Articles\Quality%20of%20work%20life%20The%20determinants%20of%20job%20satisfaction%20and%20job%20retention%20among%20RNs%20and%20OHPs.pdf) | 2 | To examine the effects of demographic characteristics on the QWL dimensions and satisfaction, and the relationships between the QWL dimensions and satisfaction, and between satisfaction and retention among RNs and OHPs. | 0 | Not mentioned | YES | 2 | survey questionnaire included: QWL, satisfaction, retention, and sociodemographic | 2 | Multivariate analysis of variance and structural equation modelling | 2 | Found positive satisfaction with work life/home life, work design, work context, and work world | 2 | Used 95% confidence interval | 0 | N/A | 2 | Consistency | 0 | N/A | 12 |
| 67 | Leadership styles and job satisfaction among healthcare providers in primary health care centres | [file:///C:/Users/mtok1/OneDrive/%D8%B3%D8%B7%D8%AD%20%D8%A7%D9%84%D9%85%D9%83%D8%AA%D8%A8/Included%20Articles/Leadership%20styles%20and%20job%20satisfaction%20among%20healthcare%20providers%20in%20primary%20health%20care%20centers.pdf](file:///C:\Users\mtok1\OneDrive\Ø³Ø·Ø­%20Ø§ÙÙÙØªØ¨\Included%20Articles\Leadership%20styles%20and%20job%20satisfaction%20among%20healthcare%20providers%20in%20primary%20health%20care%20centers.pdf) | 2 | To explore the relationship between managers' leadership styles and job satisfaction among healthcare workers in primary healthcare centres (PHCCs) in Aseer Region, Saudi Arabia. | 2 | Cluster sample technique and systematic random sampling technique | Yes | 2 | Survey instruments included a brief socio-demographic survey questionnaire, the "Multifactor Leadership Questionnaire, Form 6-S", and the validated Arabic version of "Job Satisfaction Survey Questionnaire" | 0 | Descriptive statistics, Pearson's correlation, and Structural Equation Modelling | 2 | Found positive satisfaction with laissez-faire leadership style, TA leadership, and TF leadership | 2 | Used 95% confidence interval | 0 | The causal relationship between factors affecting satisfaction cannot be confirmed as the study design is cross-sectional, which is good for hypothesis generation, rather than hypothesis testing | 2 | Consistency | 2 | Important for mangers of PHCCs | 14 |
| 68 | Job satisfaction in PHC Kuwait | [file:///C:/Users/mtok1/OneDrive/%D8%B3%D8%B7%D8%AD%20%D8%A7%D9%84%D9%85%D9%83%D8%AA%D8%A8/Included%20Articles/Job%20satisfaction%20in%20PHC%20Kuwait.pdf](file:///C:\Users\mtok1\OneDrive\Ø³Ø·Ø­%20Ø§ÙÙÙØªØ¨\Included%20Articles\Job%20satisfaction%20in%20PHC%20Kuwait.pdf) | 2 | To assess employee satisfaction in Kuwait regarding their opinion of their job. | 2 | random sample | YES | 1 | A self-administered structure questionnaire adapted from a tool used by Canadian accreditation with minor changes to the wording | 0 | SPSS | 2 | Found positive satisfaction with job nature, training and development, your co-worker, your supervisor, your head of the clinic, safety and health, your overall experience, your overall opinion (quality grade and safety grade) | 2 | Used 95% confidence interval | 2 | One limitation of the present study was lack of willingness among health care staff to participate in this study and the lack of reviewing job satisfaction with working experience and salary income | 0 | N/A | 2 | Important for the managers in the primary health care centres and policy makers | 13 |
| 69 | Motivation and Job Satisfaction of Pharmacists in Four Hospitals in Saudi Arabia | [file:///C:/Users/mtok1/OneDrive/%D8%B3%D8%B7%D8%AD%20%D8%A7%D9%84%D9%85%D9%83%D8%AA%D8%A8/Included%20Articles/Motivation%20and%20Job%20Satisfaction%20of%20Pharmacists%20in%20Four%20Hospitals%20in%20Saudi%20Arabia.pdf](file:///C:\Users\mtok1\OneDrive\Ø³Ø·Ø­%20Ø§ÙÙÙØªØ¨\Included%20Articles\Motivation%20and%20Job%20Satisfaction%20of%20Pharmacists%20in%20Four%20Hospitals%20in%20Saudi%20Arabia.pdf) | 2 | To find out what strongly motivate pharmacists, measure job satisfaction, determine the influencing factors, and to compare it with pharmacy managers’ and supervisors’ opinion. | 0 | Not mentioned | YES | 2 | quantitative anonymous questionnaire for Managers/ Supervisors and qualitative semi-longitudinal self-completion questionnaire  for pharmacists including sociodemographic, ‘Job Satisfaction Questionnaire for Managers/Supervisors’, ‘Job Satisfaction and Job Attractiveness’ | 0 | descriptive statistics and SPSS | 2 | Found positive satisfaction with salary, job security, promotion, vacation, motivational talks, recognition, financial reward, job satisfaction, and job attractiveness | 2 | Used 95% confidence interval | 0 | Sample was heterogenic, data collection was from two large government hospitals, one small private hospital and one large private hospital. The response rate from each hospital was not identified; all results collected were analysed with no comparison between private and government sectors. Not all factors from Hertzberg’s two-factor motivation theory were looked at; the study focused on three motivators and three hygiene factors only. It is a one-year longitudinal study, Occupational stress was not included in this study. | 2 | Consistency | 0 | N/A | 10 |
| 70 | Global Survey of the Roles, Satisfaction, and Barriers of Home Health Care Nurses on the Provision of Palliative Care | [file:///C:/Users/mtok1/OneDrive/%D8%B3%D8%B7%D8%AD%20%D8%A7%D9%84%D9%85%D9%83%D8%AA%D8%A8/Maybe%20articles/Global%20Survey%20of%20the%20Roles,%20Satisfaction,%20and%20Barriers%20of%20Home%20Health%20Care%20Nurses%20on%20the%20Provision%20of%20Palliative%20Care.pdf](file:///C:\Users\mtok1\OneDrive\Ø³Ø·Ø­%20Ø§ÙÙÙØªØ¨\Maybe%20articles\Global%20Survey%20of%20the%20Roles,%20Satisfaction,%20and%20Barriers%20of%20Home%20Health%20Care%20Nurses%20on%20the%20Provision%20of%20Palliative%20Care.pdf) | 2 | To globally examine home health care nurses' practice, satisfaction, and barriers, regarding existing palliative home care provision. | 1 | convenience sample | YES | 2 | seven sections, and includes quantitative and open-ended questions addressing nurse demo-graphics, patient population information, home health care duties, satisfaction with and barriers to provision of home health care delivery and PC, community resources available, barriers to educational opportunities, and desired learning methods. Three sections include Duties, Barriers, and Satisfaction. | 0 | descriptive analysis, an exploratory factor analysis, item-response theory, and mean and standard deviations | 0 | N/A | 2 | Used 95% confidence interval | 0 | Data were collected using a convenience sample; probability sampling methods were not incorporated. Most nurses practiced in urban settings; those working in more remote areas were not surveyed. Some questions (specific countries) had missing data due to translation issues, resulting in non-random missing information. Other data points were misinterpreted. WHO low-income countries and level 1 and 2PCD countries were not well represented. To protect anonymity, researchers collapsed PCD levels 1 and 2, making interpretations more challenging. | 0 | N/A | 0 | N/A | 7 |

| **SISTEMATIC REVIEW** | | |  |  |  |  |  |  |  |  |  |  |  |  |  |  |  |  |  |  |  |  |  |  |  |  |  |  |
| --- | --- | --- | --- | --- | --- | --- | --- | --- | --- | --- | --- | --- | --- | --- | --- | --- | --- | --- | --- | --- | --- | --- | --- | --- | --- | --- | --- | --- |
|  |  |  |  |  |  |  |  |  |  |  |  |  |  |  |  |  |  |  |  |  |  |  |  |  |  |  |  |  |
|  |  |  | (A) Are the results of the review valid? | | | | | | | | | |  | (B) What are the results? | | |  | (C) Will the results help locally? | | | | | | | | |  |  |
|  |  |  |  |  |  |  |  |  |  |  |  |  |  |  |  |  |  |  |  |  |  |  |  |  |  |  |  |  |
|  |  |  | Question 1 | | Question 2 | |  | Question 3 | | Question 4 | | Question 5 | | Question 6 | | Question 7 | | Question 8 | | Question 9 | | Question 10 | | Question 11 | | Question 12 | |  |
|  | NAME ARTICLE | LINK ARTICLE | Did the review address a clearly focused question? | | Did the authors look for the right type of papers? | | IS WORTH TO CONTINUING? | Do you think all the important, relevant studies were included? | | Did the review’s authors do enough to assess the quality of the included studies? | | If the results of the review have been combined,  was it reasonable to do so? | | What are the overall results of the review? | | How precise are the results? | | Can the results be applied to the local population? | | Were all important outcomes considered? | | Are the benefits worth the harms and costs? | |  | |  | |  |
|  |  |  | Score | Description | Score | Description |  | Score | Description | Score | Description | Score | Description | Score | Description | Score | Description | Score | Description | Score | Description | Score | Description | Score | Description | Score | Description | TOTAL SCORE |
| 71 | Burnout and job satisfaction among critical care nurses in Saudi Arabia and their contributing factors: A scoping review | [file:///C:/Users/mtok1/OneDrive/%D8%B3%D8%B7%D8%AD%20%D8%A7%D9%84%D9%85%D9%83%D8%AA%D8%A8/Included%20Articles/Burnout%20and%20job%20satisfaction%20among%20critical%20care%20nurses%20in%20Saudi%20Arabia%20and%20their%20contributing%20factors%20A%20scoping%20review.pdf](file:///C:\Users\mtok1\OneDrive\Ø³Ø·Ø­%20Ø§ÙÙÙØªØ¨\Included%20Articles\Burnout%20and%20job%20satisfaction%20among%20critical%20care%20nurses%20in%20Saudi%20Arabia%20and%20their%20contributing%20factors%20A%20scoping%20review.pdf) | 2 | assess the degree of well-being of nurses working in critical care settings in Saudi Arabia by evaluating their levels of burnout and job satisfaction, as well as the factors that contribute to them. | 2 |  | YES | 2 |  | 2 |  | 2 |  | 2 |  | 2 |  | 2 |  | 2 |  | 2 |  | 0 |  | 0 |  | 20 |
| 72 | The Impact of Transformational Leadership on Job Satisfaction and Organisational Commitment Among Hospital Staff: A Systematic Review | [file:///C:/Users/mtok1/OneDrive/%D8%B3%D8%B7%D8%AD%20%D8%A7%D9%84%D9%85%D9%83%D8%AA%D8%A8/Included%20Articles/The%20Impact%20of%20Transformational%20Leadership%20on%20Job%20Satisfaction%20and%20Organisational%20Commitment%20Among%20Hospital%20Staff%20A%20Systematic%20Review.pdf](file:///C:\Users\mtok1\OneDrive\Ø³Ø·Ø­%20Ø§ÙÙÙØªØ¨\Included%20Articles\The%20Impact%20of%20Transformational%20Leadership%20on%20Job%20Satisfaction%20and%20Organisational%20Commitment%20Among%20Hospital%20Staff%20A%20Systematic%20Review.pdf) | 2 | To examine the impact of transformational leadership on job satisfaction and organisational commitment among hospital staff. | 2 |  | YES | 2 |  | 2 |  | 2 |  | 2 |  | 2 |  | 0 | Due to the time shortage, the authors of the assessed studies have not been communicated to justify the evaluation and avoid unwanted disadvantages, Qualitative studies were not involved due to the volume of quantitative studies, this review was limited by the potential of reporting bias that may exist as the researcher included only the results for job satisfaction and organisational commitment and ignored other factors related to the studies. | 2 |  | 2 |  | 0 |  | 0 |  | 18 |

|  | **QUALITATIVE RESEARCH** | | | | |  | |  | |  | | |  |  |  | | |  | |  | |  | |  | | |  | |  | | |  | |  | |  | |  | |  | |  | |  | |  | | |  | | |  |
| --- | --- | --- | --- | --- | --- | --- | --- | --- | --- | --- | --- | --- | --- | --- | --- | --- | --- | --- | --- | --- | --- | --- | --- | --- | --- | --- | --- | --- | --- | --- | --- | --- | --- | --- | --- | --- | --- | --- | --- | --- | --- | --- | --- | --- | --- | --- | --- | --- | --- | --- | --- | --- |
|  |  |  |  | (A) Screening Questions | | | | | | | | | | (B) Detailed questions | | | | | | | | | | | | | | | | | | | | | | | | | | | | | | | | | |  | | | | |
|  |  |  |  |  |  | |  | |  | | |  | |  | |  |  | |  | |  | |  | | |  | |  | | |  | |  | |  | |  | |  | |  | |  | |  | |  | | |  |  |  |
|  |  |  |  | Question 1 | | | Question 2 | | | |  | | | Question 3 | | | Question 4 | | | | Question 5 | | | | Question 6 | | | | | Question 7 | | | | | Question 8 | | | | Question 9 | | | | Question 10 | | | |  | | | |  |  |
|  |  | NAME ARTICLE | LINK ARTICLE | Was there a clear statement of the aims  of the research? | | | Is a qualitative methodology appropriate? | | | | IS WORTH TO CONTINUING? | | | Was the research design appropriate to  address the aims of the research? | | | Was the recruitment strategy appropriate to the aims of the research? | | | | Was the data collected in a way that addressed  the research issue? | | | | Has the relationship between researcher and participants been adequately considered? | | | | | Have ethical issues been taken into consideration? | | | | | Was the data analysis sufficiently rigorous? | | | | Is there a clear statement of findings? | | | | How valuable is the research? | | | |  | | | |  |  |
|  |  |  |  | Score | Description | | Score | | Description | | |  | | Score | | Description | Score | | Description | | Score | | Description | | | Score | | Description | | | Score | | Description | | Score | | Description | | Score | | Description | | Score | | Description | | TOTAL SCORE | | |  |  |  |
|  | 73 | Home and expatriate nurses' perceptions of job satisfaction: Qualitative findings | [file:///C:/Users/mtok1/OneDrive/%D8%B3%D8%B7%D8%AD%20%D8%A7%D9%84%D9%85%D9%83%D8%AA%D8%A8/Included%20Articles/Home%20and%20expatriate%20nurses%E2%80%99%20perceptions%20of%20job%20satisfaction_%20Qualitative%20findings.pdf](file:///C:\Users\mtok1\OneDrive\Ø³Ø·Ø­%20Ø§ÙÙÙØªØ¨\Included%20Articles\Home%20and%20expatriate%20nursesâ%20perceptions%20of%20job%20satisfaction_%20Qualitative%20findings.pdf) | 2 | To increase understanding of the factors influencing job satisfaction of overseas nurses working in Saudi Arabia comparing the perceptions of home and expatriate nurses. | | 2 | | YES | | | YES | | 2 | | YES | 2 | | 26 participants from different nationalities | | 2 | |  | | | 2 | | no direct relation between participant and authors | | | 2 | | Before each interview, explained the protocol and intentions to the participant, participants were informed of the nature of the study and its objectives. | | 2 | | The researches taken measures, such as peer and member checking, stringent data analysis and interpretation procedures to enhance interpretative rigor and trustworthiness | | 2 | | Five themes were identified that differentiated the perceptions of expatriates regarding their job satisfaction from those of the home nurses: separation from family, language and communication, fairness of remuneration, moving into the future and professionalism. | | 2 | | Increase understanding the perceptions of home and expatriate nurses working in S.A. based on Job satisfaction and add to wider existing literature on the experiences of expatriate nurses | | 20 | | |  |  |  |
|  | 74 | FACTORS INFLUENCING JOB SATISFACTION among RECENTLY QUALIFIED RESIDENT DOCTORS: A QUALITATIVE STUDY | [file:///C:/Users/mtok1/OneDrive/%D8%B3%D8%B7%D8%AD%20%D8%A7%D9%84%D9%85%D9%83%D8%AA%D8%A8/Included%20Articles/H.%20Almansour%20-%20Factors%20Influencing%20Job%20Satisfaction%20Among%20Recently%20Qualified%20Resident%20Doctors_%20A%20qualitative%20study.pdf](file:///C:\Users\mtok1\OneDrive\Ø³Ø·Ø­%20Ø§ÙÙÙØªØ¨\Included%20Articles\H.%20Almansour%20-%20Factors%20Influencing%20Job%20Satisfaction%20Among%20Recently%20Qualified%20Resident%20Doctors_%20A%20qualitative%20study.pdf) | 2 | To explore the most crucial factors that influence job satisfaction among recently qualified resident doctors in Saudi Arabia. | | 2 | | YES | | | YES | | 2 | | YES | 2 | | 25 qualified resident doctors from various area of specialization | | 2 | |  | | | 2 | |  | | | 2 | | The ethical approval for this study was granted by the ethics committee, university of Ha'il | | 2 | |  | | 2 | | The following seven themes emerged from the data: education and development, recognition and respect, professionalism, workload, healthcare facilities, patient adherence, and salary. | | 2 | | enhanced knowledge regarding factors affecting recently qualified resident doctors | | 20 | | |  |  |  |
|  | 75 | The impact of nurse managers' leadership styles on ward staff | [file:///C:/Users/mtok1/OneDrive/%D8%B3%D8%B7%D8%AD%20%D8%A7%D9%84%D9%85%D9%83%D8%AA%D8%A8/Maybe%20articles/The%20impact%20of%20nurse%20managers'%20leadership%20styles%20on%20ward%20staff%202.pdf](file:///C:\Users\mtok1\OneDrive\Ø³Ø·Ø­%20Ø§ÙÙÙØªØ¨\Maybe%20articles\The%20impact%20of%20nurse%20managers'%20leadership%20styles%20on%20ward%20staff%202.pdf) | 2 | To explore the nature of leadership styles used by the nursing management team, as perceived by nurses working at the bedside | | 2 | | YES | | | YES | | 2 | | YES | 2 | | 35 nurses working in different specialties of a medical city in Saudi Arabia | | 2 | |  | | | 2 | |  | | | 2 | | YES | | 2 | |  | | 2 | | participants described four types of leadership styles: relational leadership, preferential leadership, communication chain leadership, and ineffectual leadership | | 2 | | This study has added to the research on the nature and effects of leadership styles in nursing. | | 20 | | |  |  |  |
